# Supplementary material for: Heterogeneous somatostatin-expressing neuron population in mouse ventral tegmental area
Source: eLife. 2020 Aug 4;9:e59328. doi: 10.7554/eLife.59328 (PMC7440918; doi:10.7554/eLife.59328)

Neurobiotin

Location

Traced morphology

Sholl curve

1
ADP

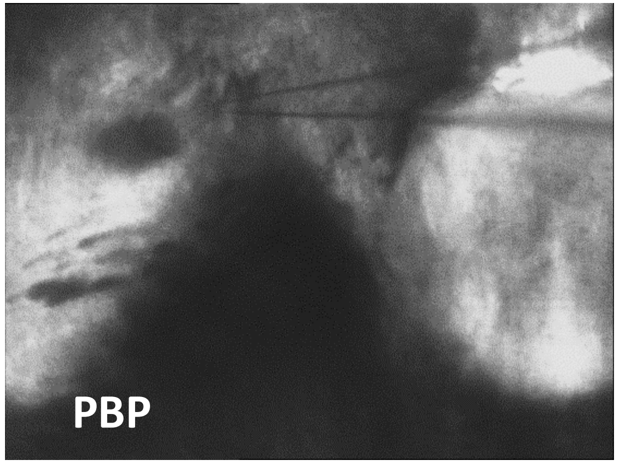

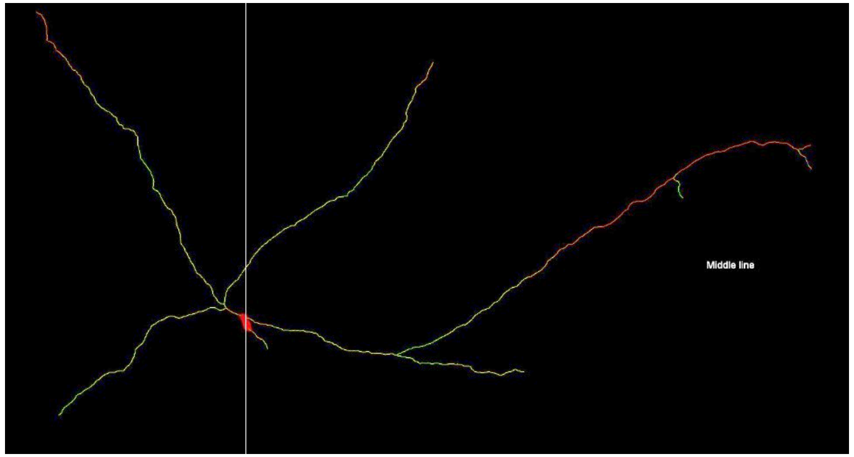

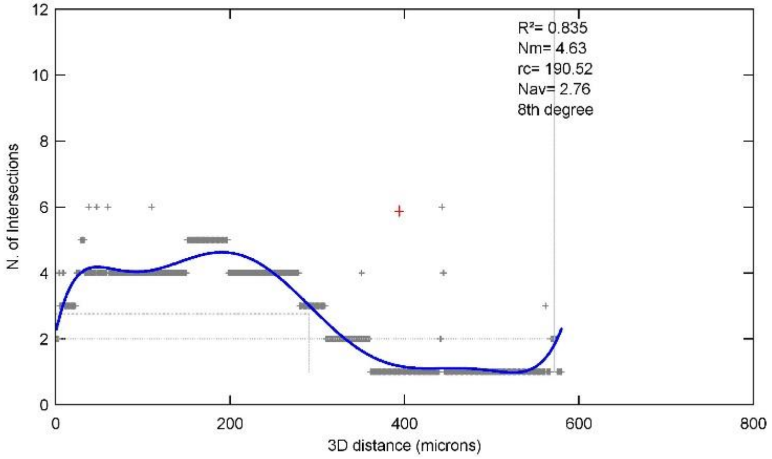

2
ADP

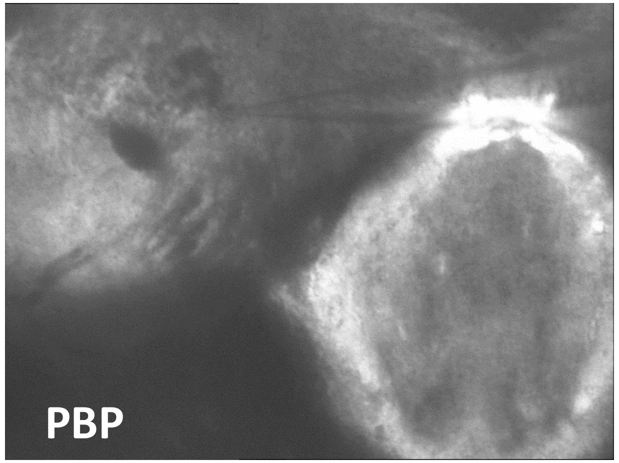

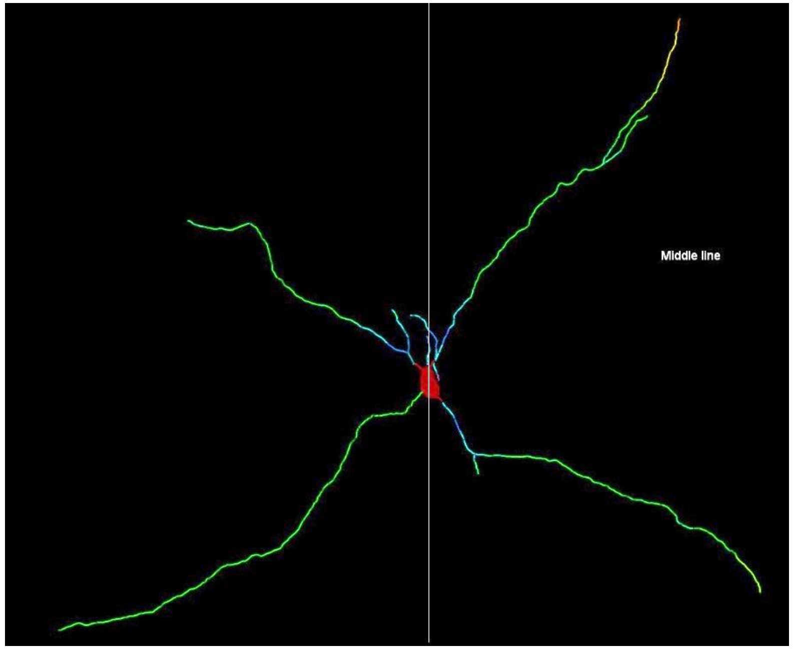

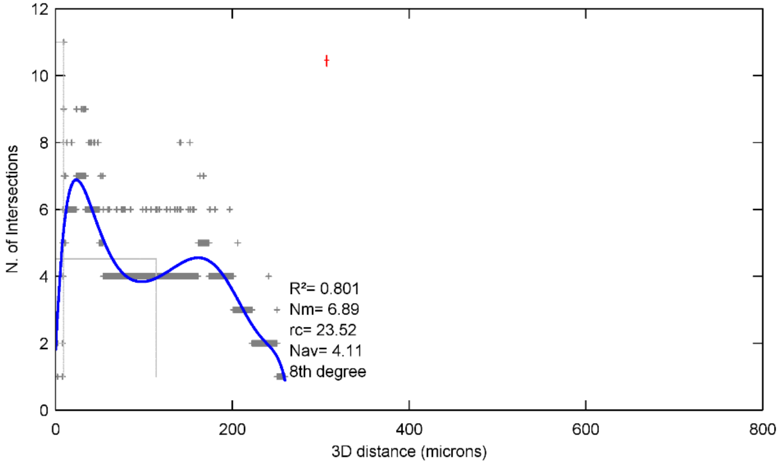

Neurobiotin

3
ADP

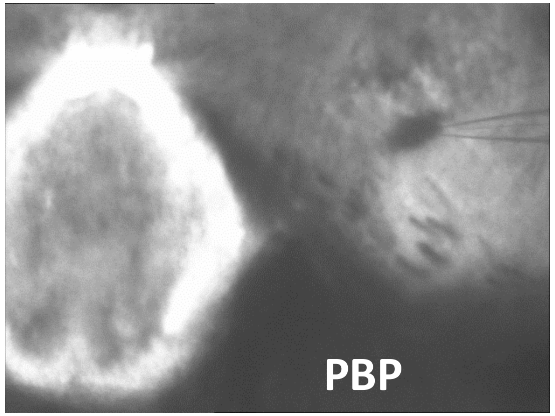

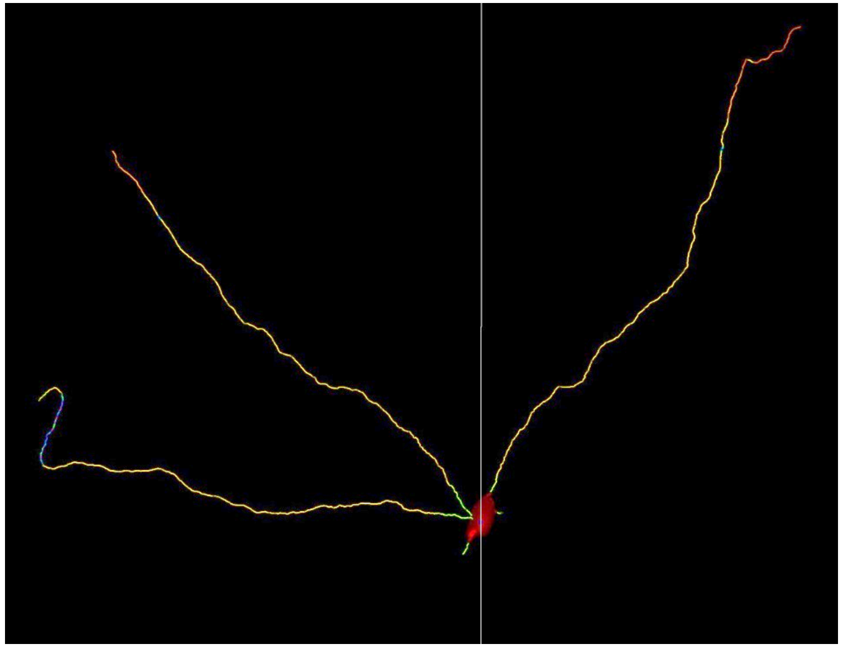

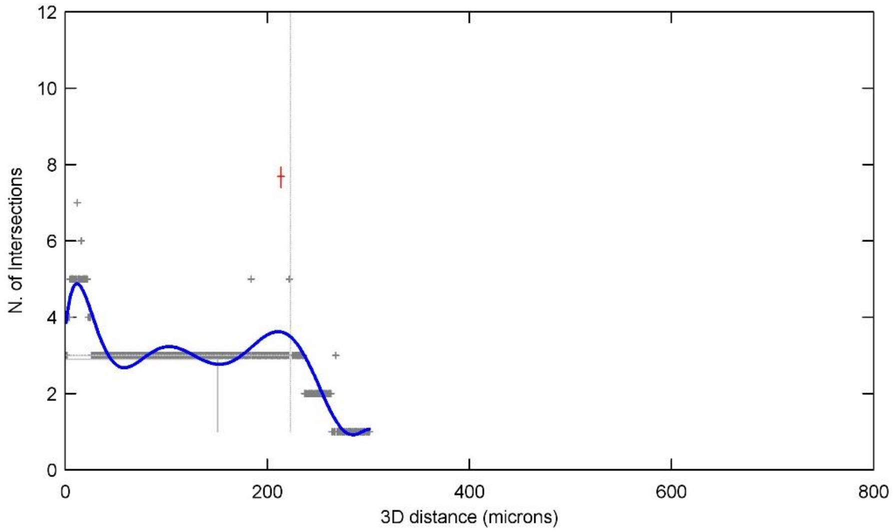

4
ADP

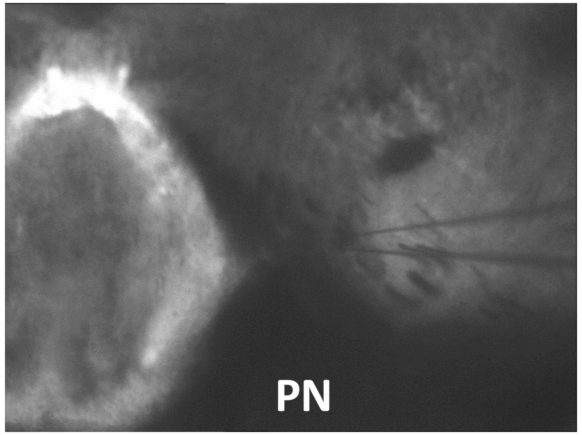

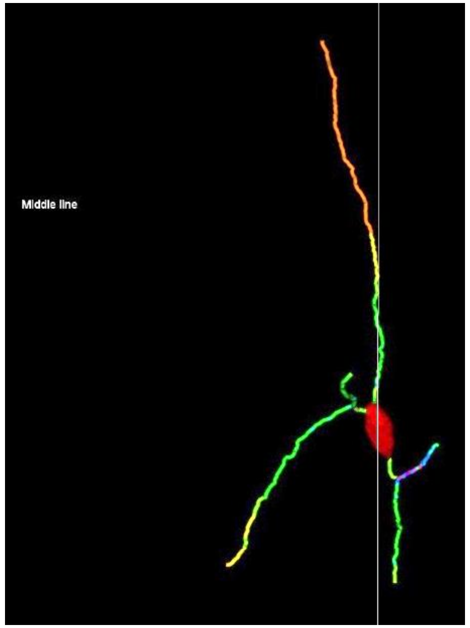

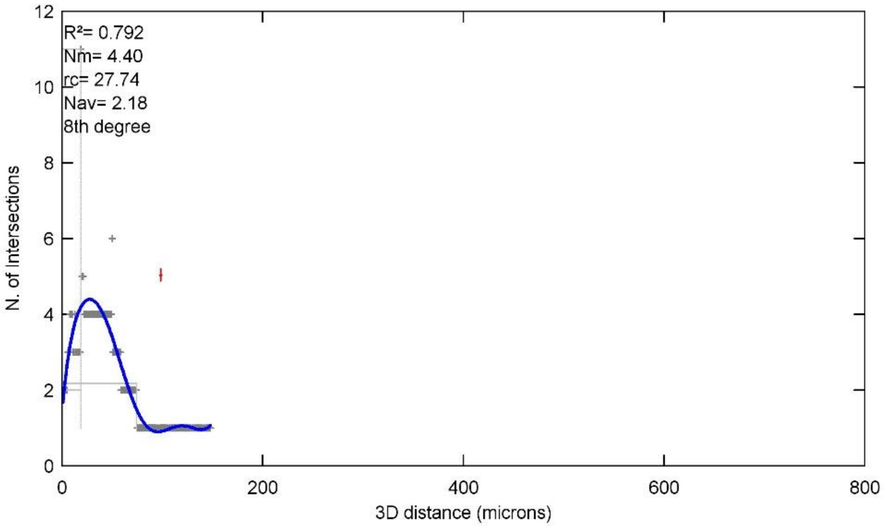

Neurobiotin

5
ADP

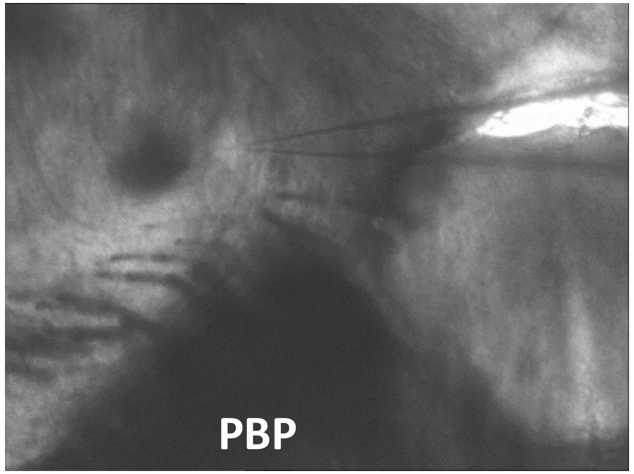

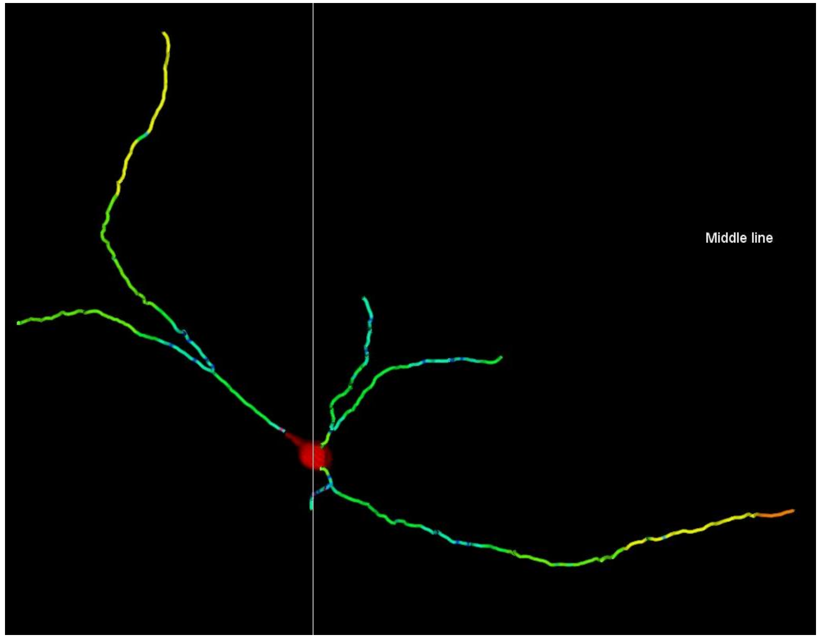

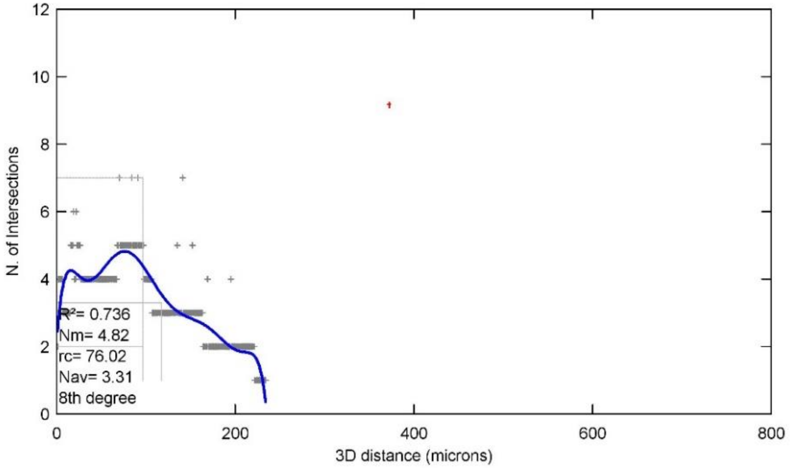

6
ADP

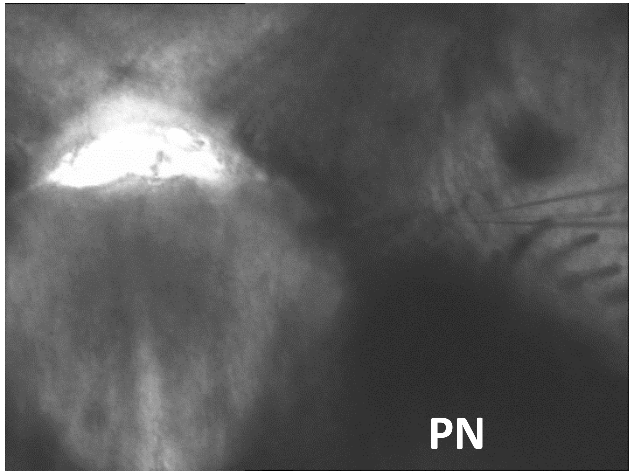

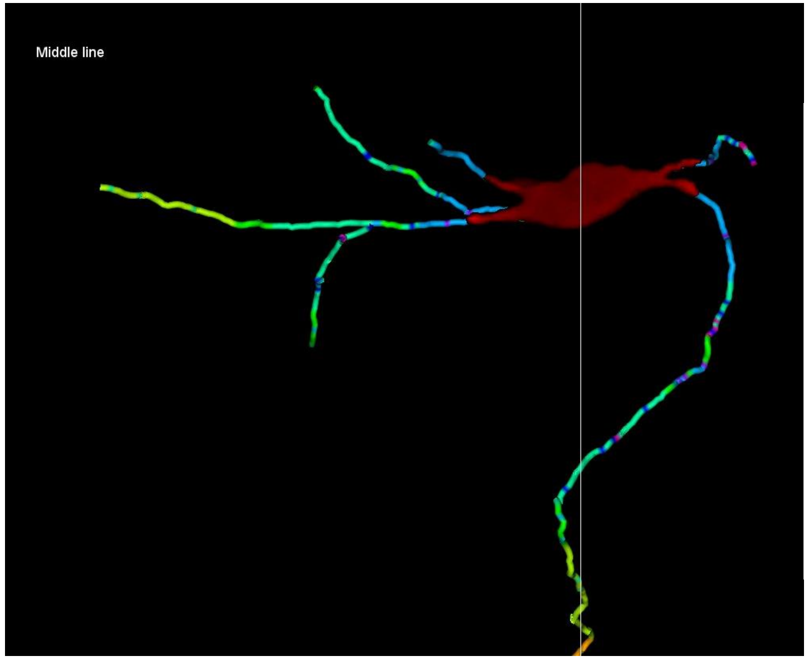

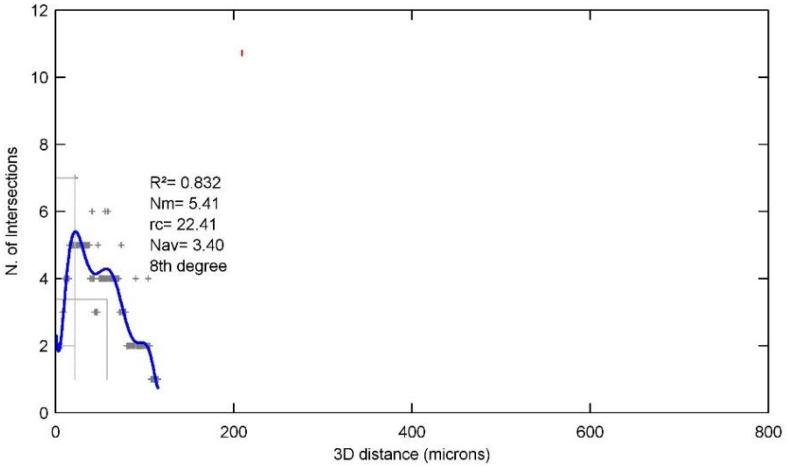

Neurobiotin

7
ADP

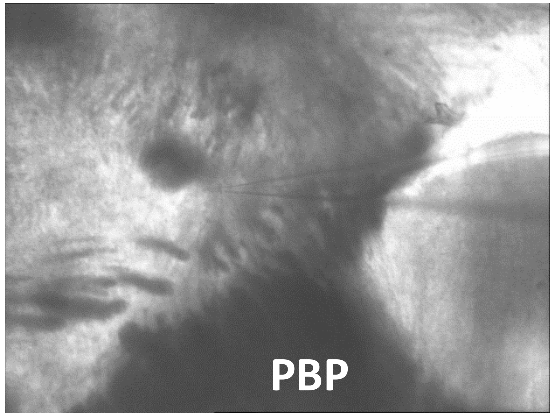

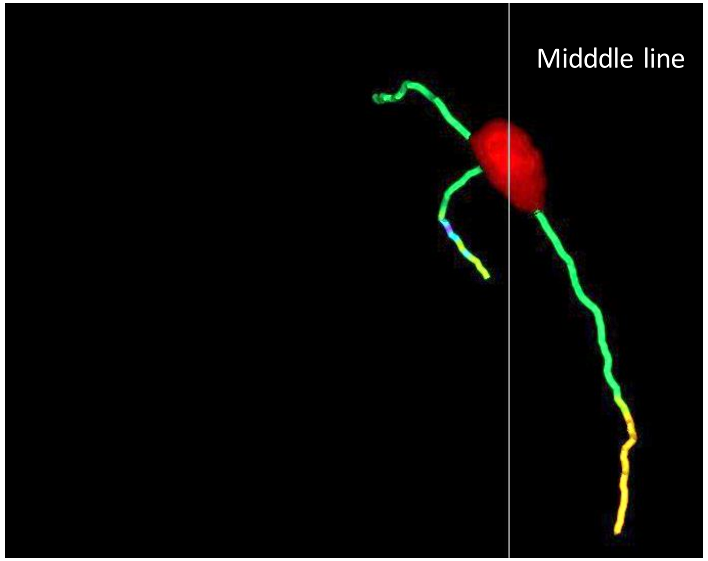

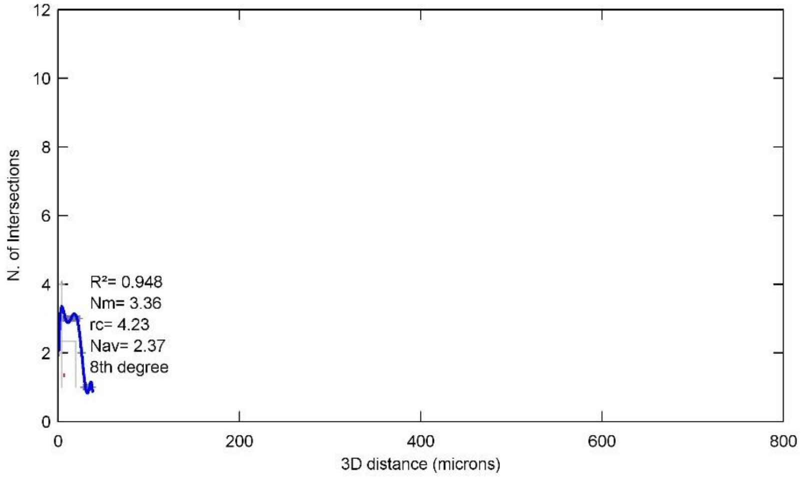

8
ADP

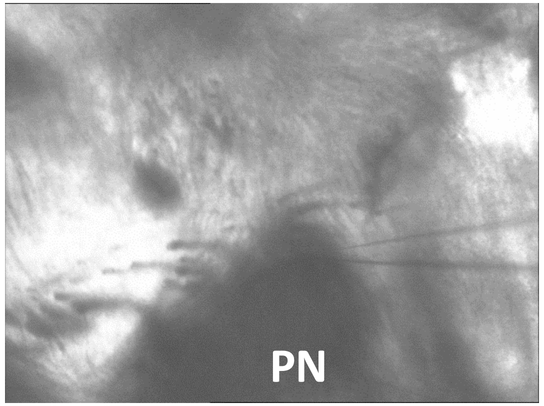

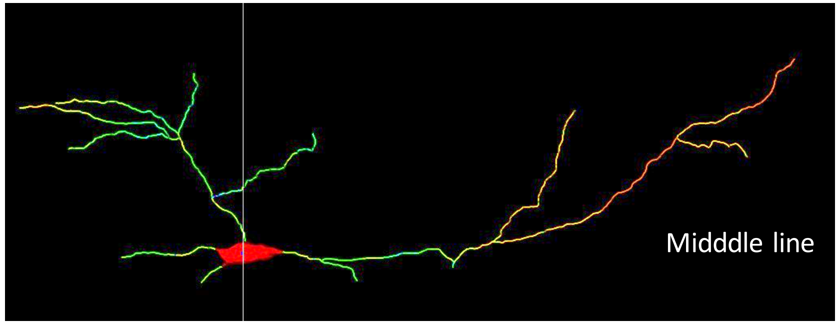

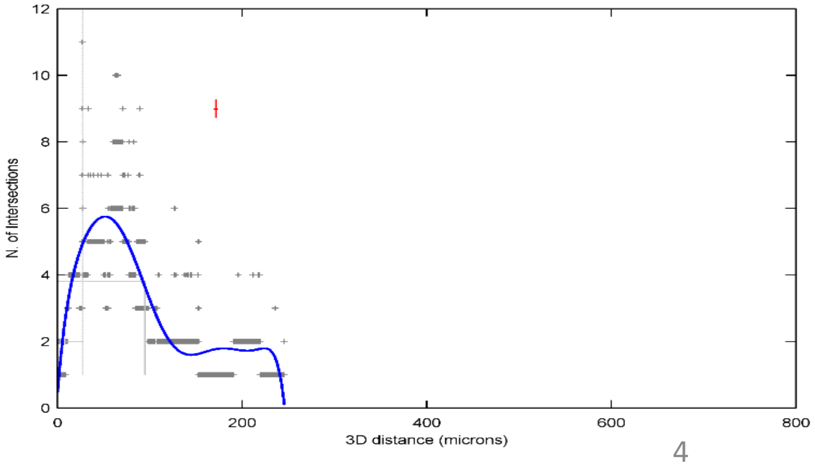

Neurobiotin

9
ADP

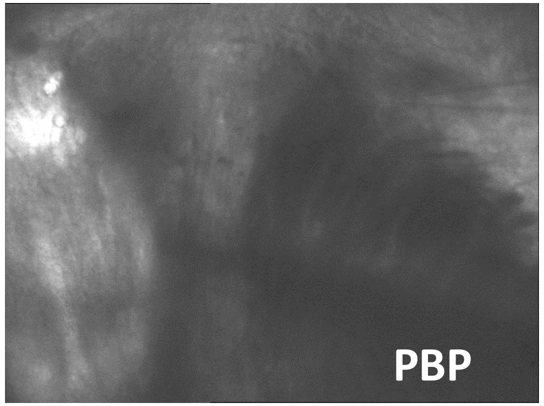

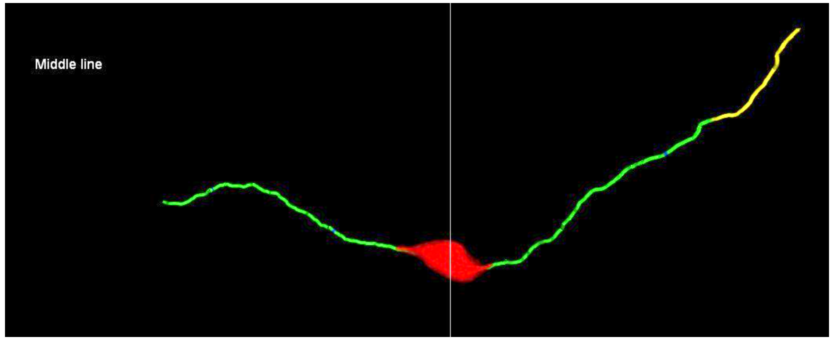

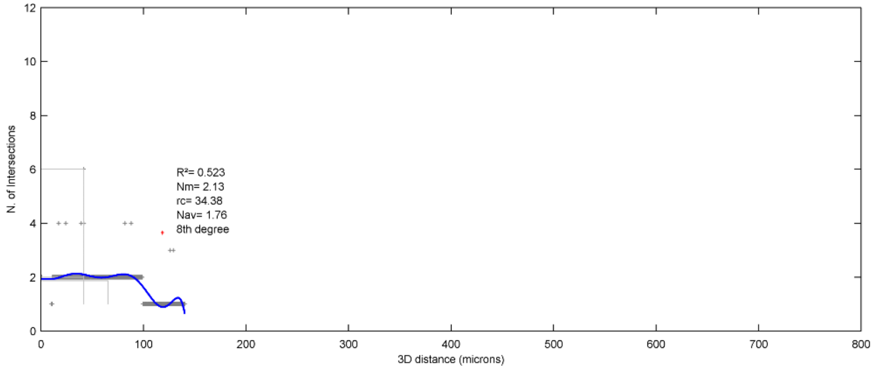

Biocytin

10
ADP

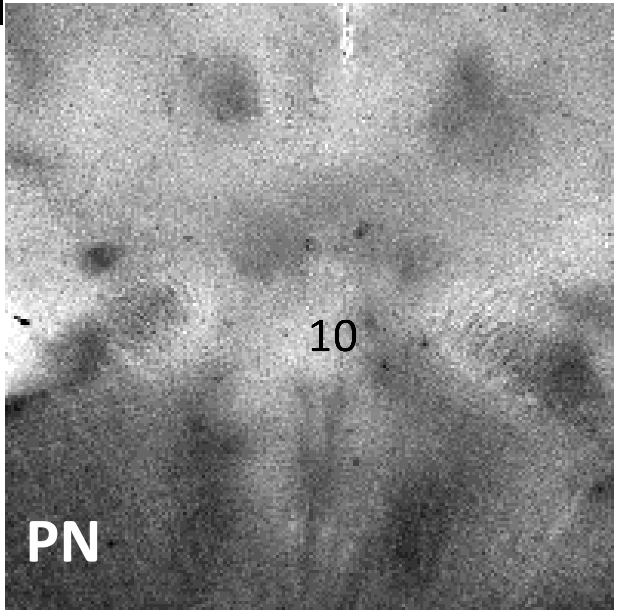

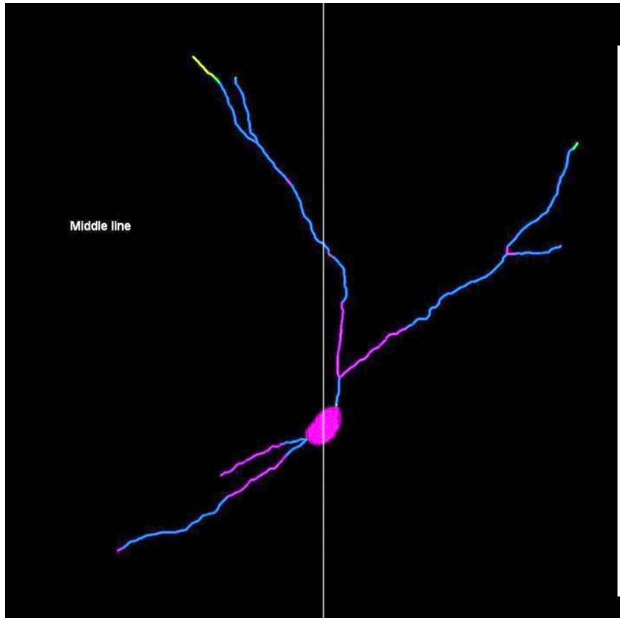

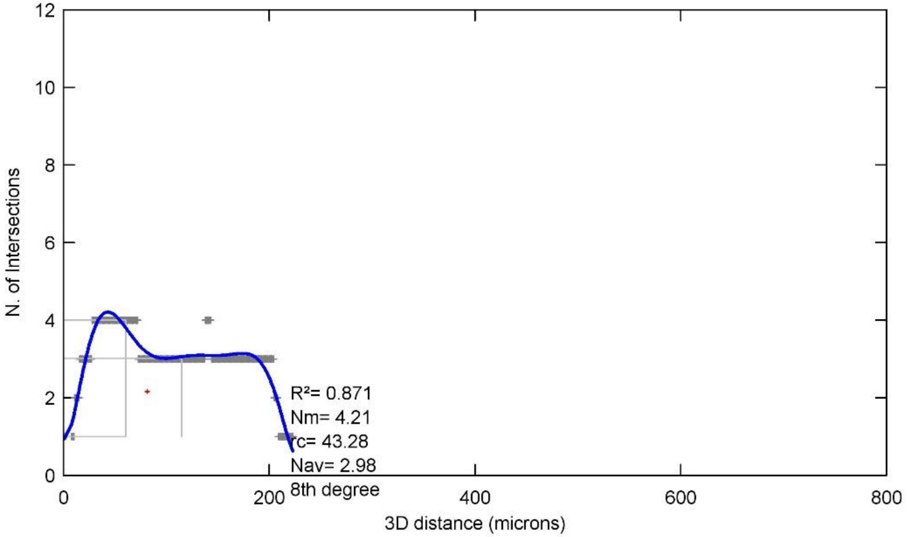

11
ADP

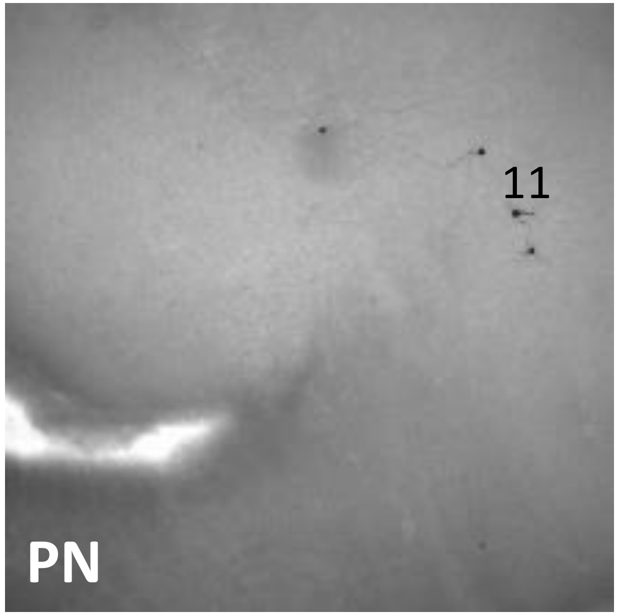

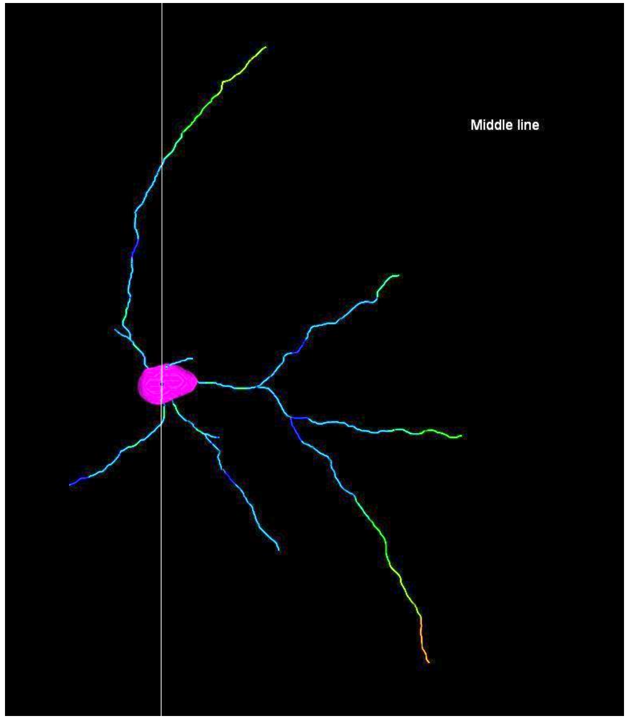

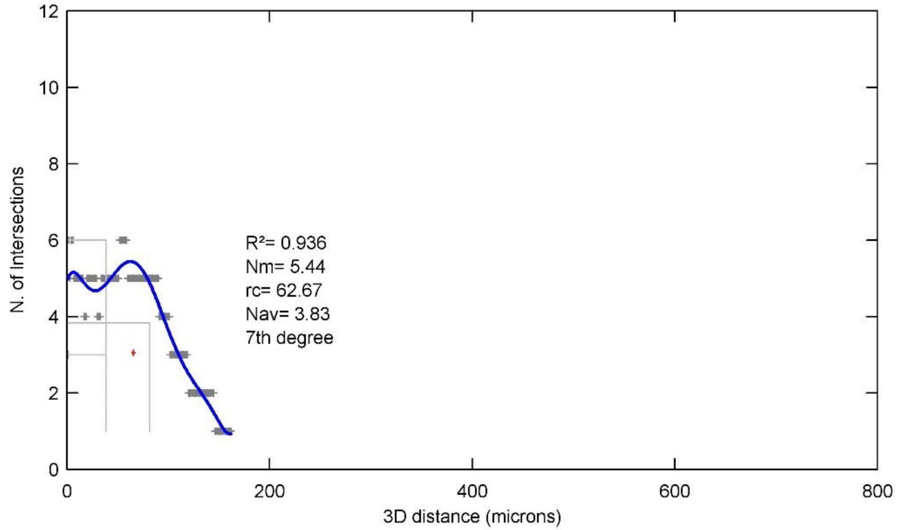

Biocytin

12
ADP

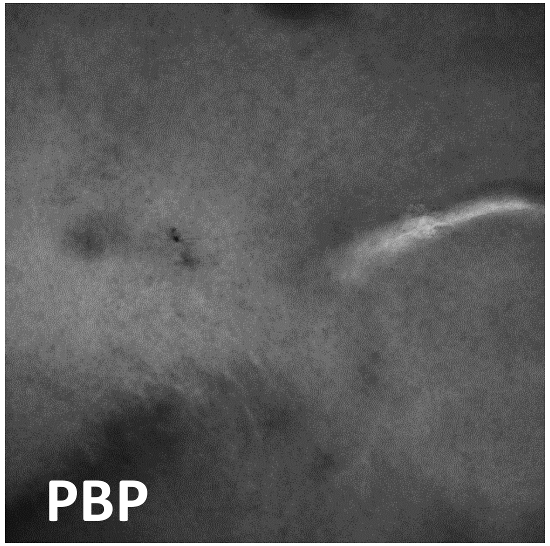

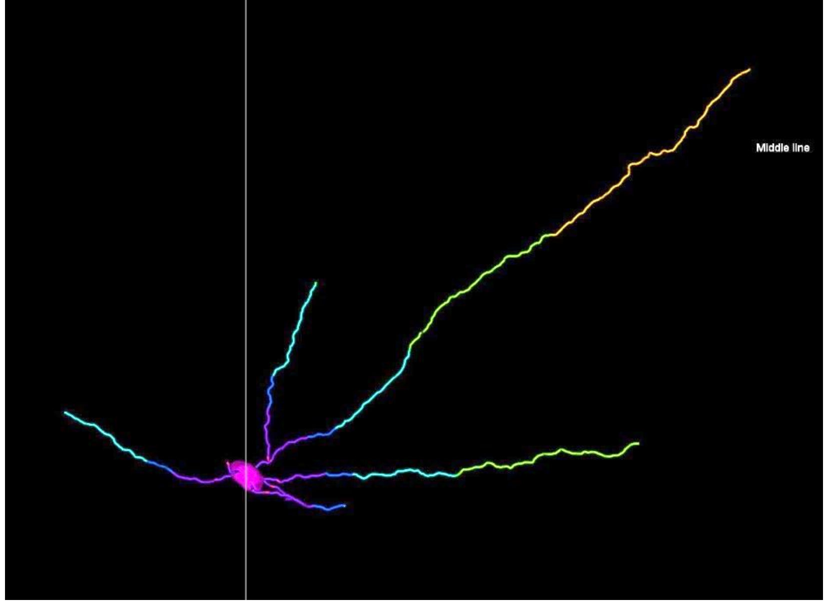

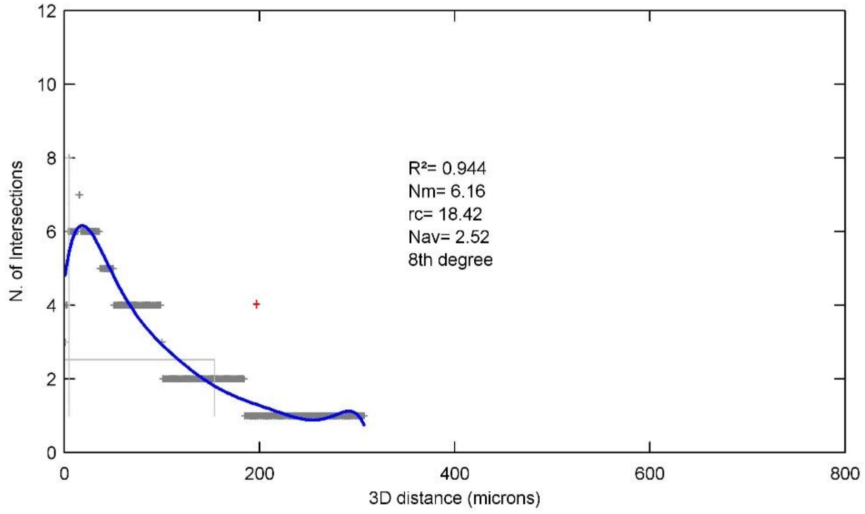

13
ADP

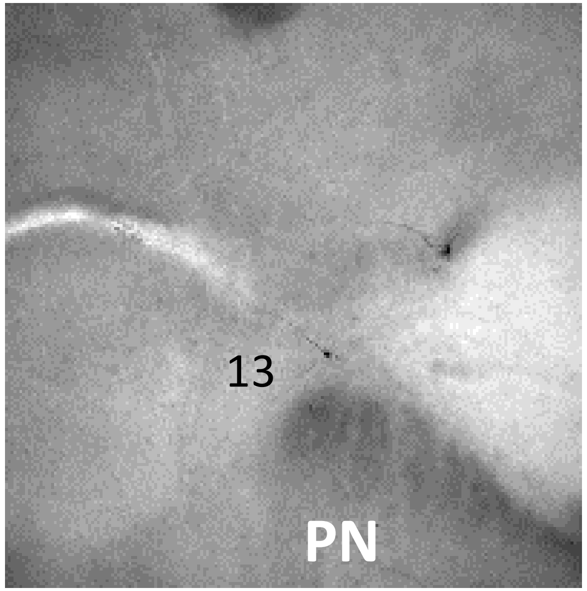

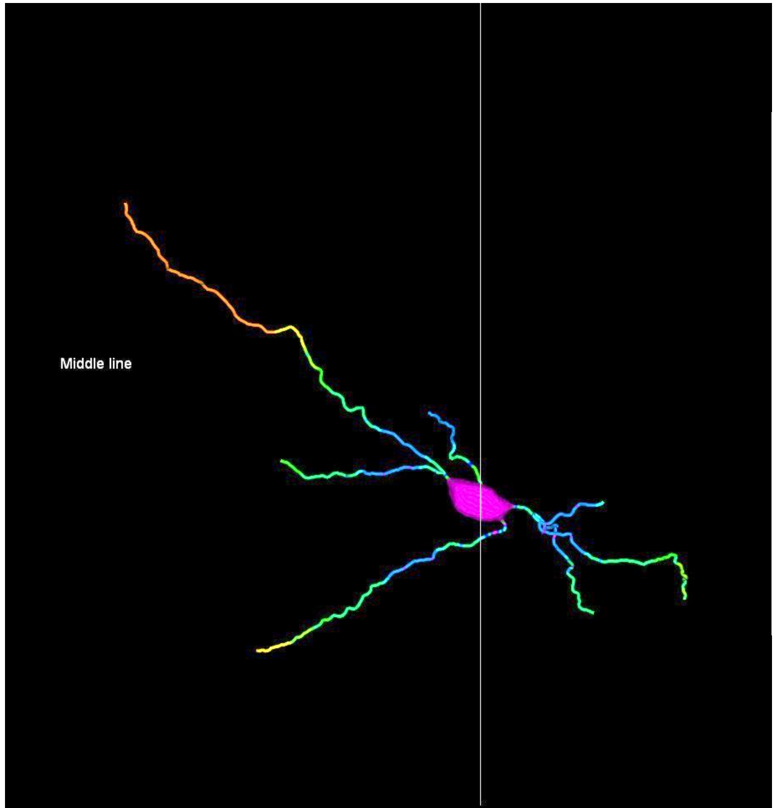

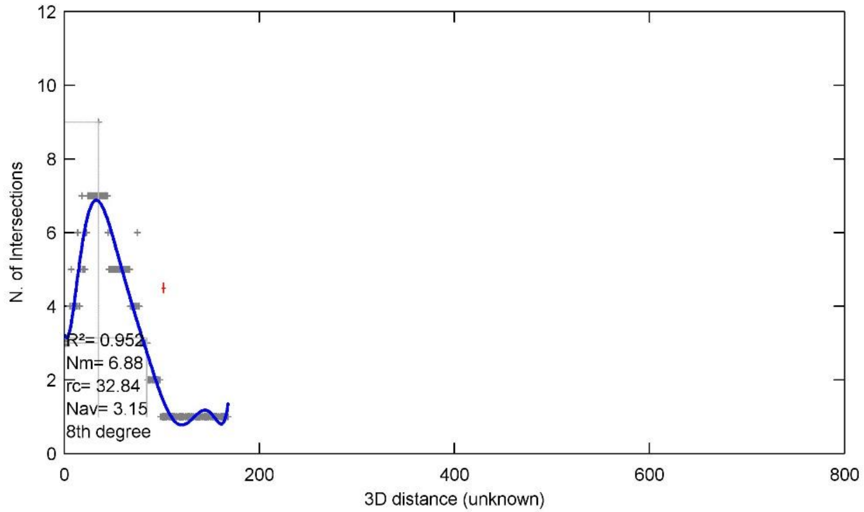

Biocytin

14
ADP

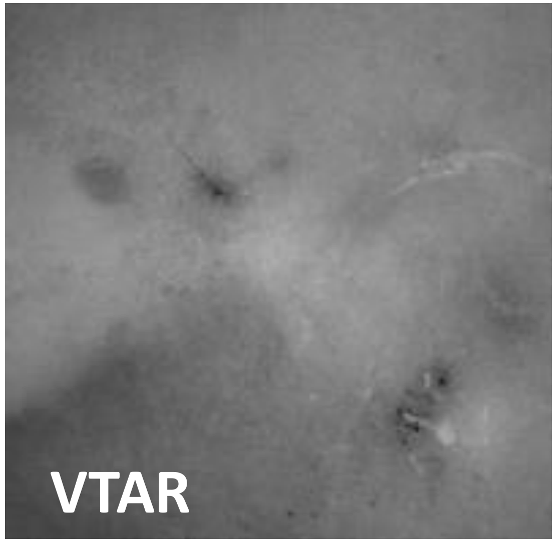

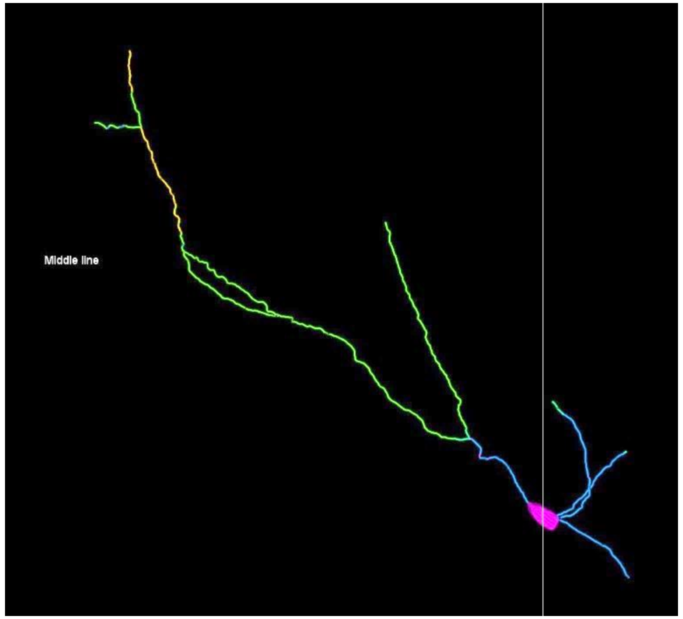

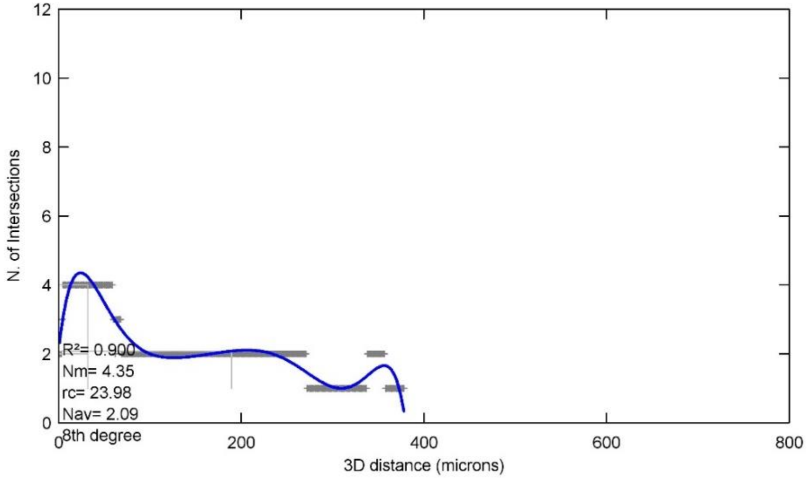

15
ADP

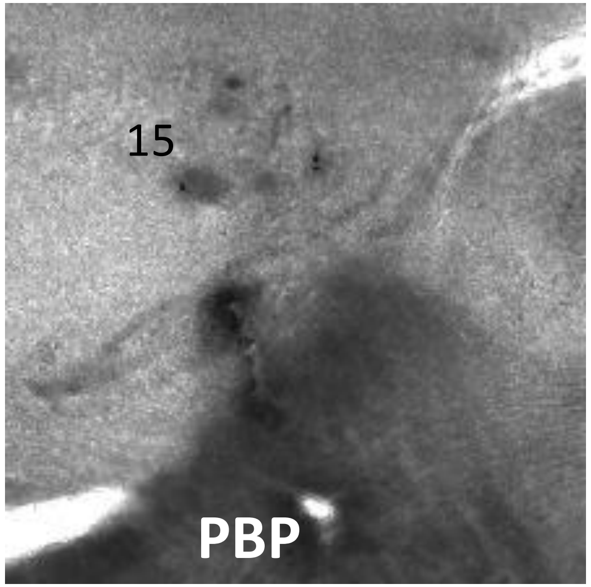

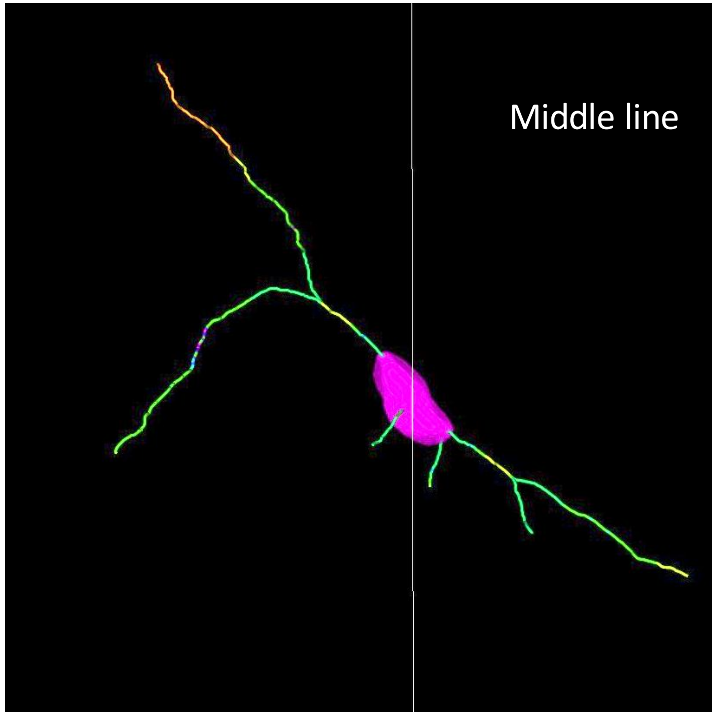

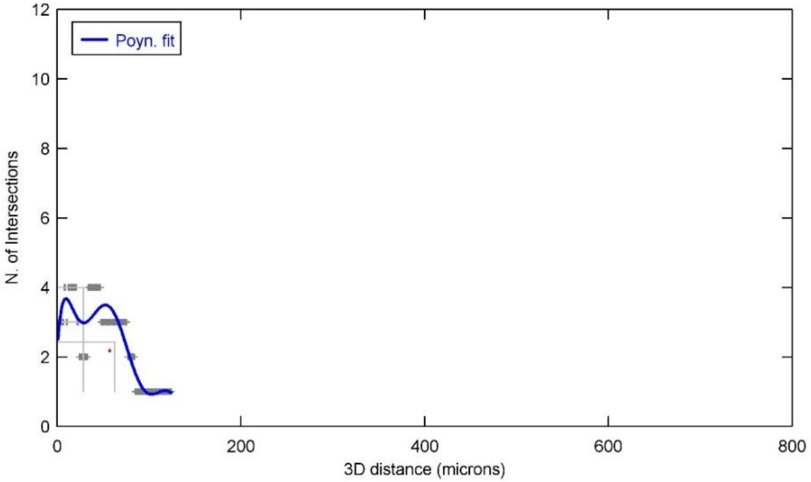

Biocytin

16
ADP

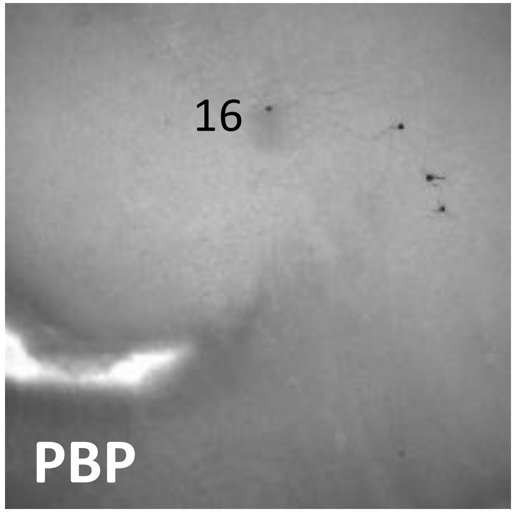

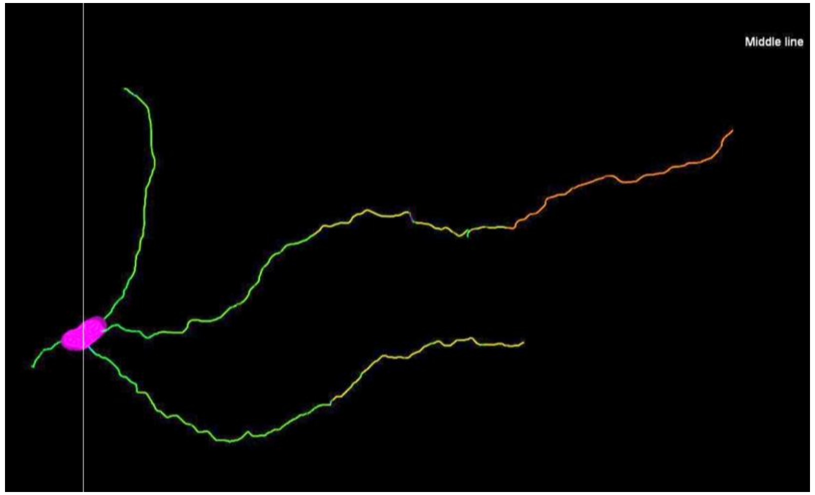

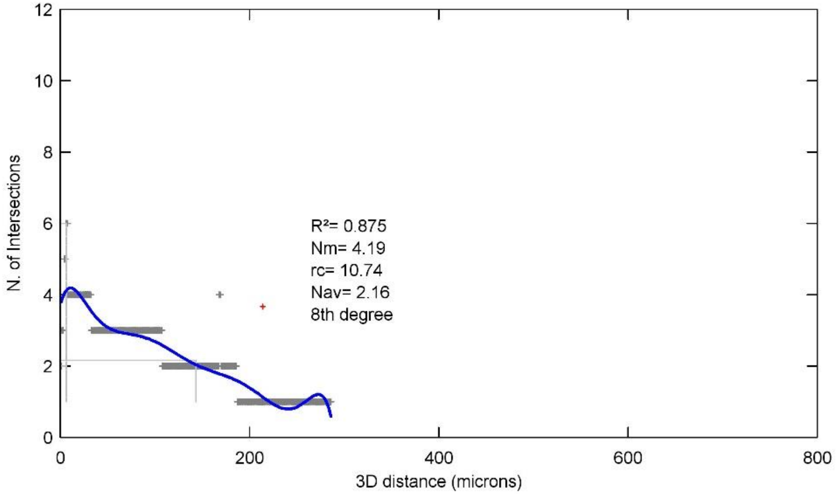

17
ADP

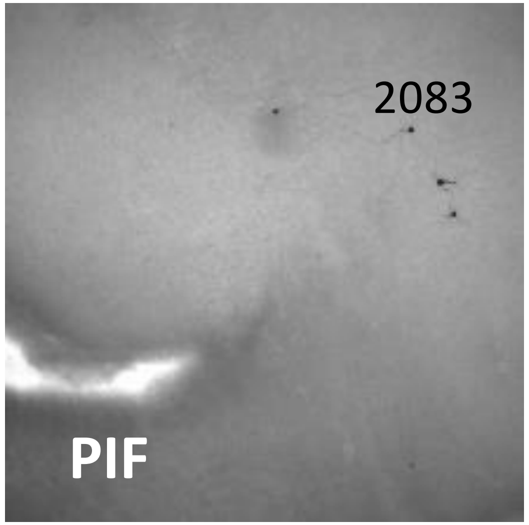

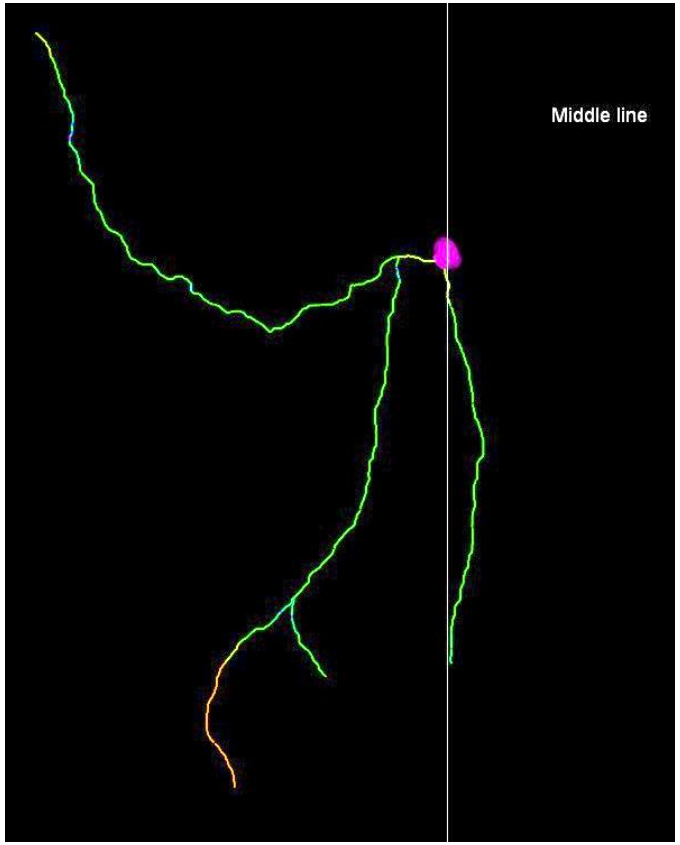

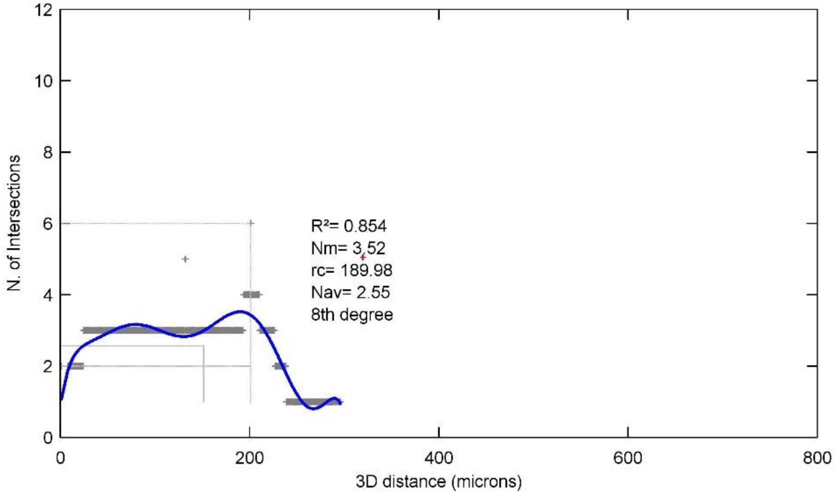

Supplement: Figure 5—source data 2. — This zip archive contains morphological images of all traced neurons grouped according their electrophysiological profiles. Each subtype’s folder contains a PDF file (with the list of neurons, their original location within the VTA, images of the traced morphology and individual Sholl curves) and two subfolders: ‘3D_gif’ – with *.gif files of the listed neurons; and ‘WaveFront_3D_obj’ – with corresponding *.obj files. *.gif files can be opened by any image viewer. *.obj files save information about the 3D model of the neurons and can be opened/reused with any 3D viewer or graphic software. [file elife-59328-fig5-data2.zip › Morphology_Source/ADP/ADP_list.pdf]
