# Supplementary material for: Heterogeneous somatostatin-expressing neuron population in mouse ventral tegmental area
Source: eLife. 2020 Aug 4;9:e59328. doi: 10.7554/eLife.59328 (PMC7440918; doi:10.7554/eLife.59328)

Neurobiotin

Location | **Traced morphology** | **Sholl curve**

1
Delayed

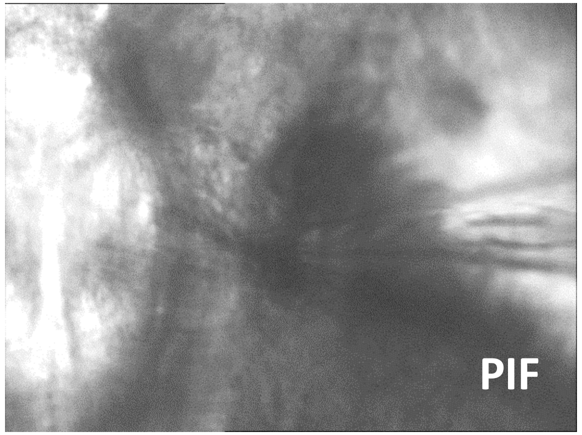

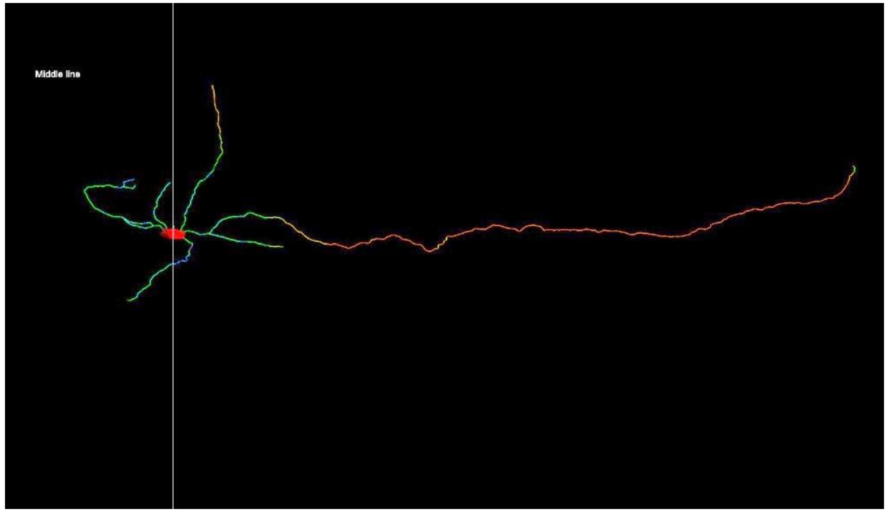

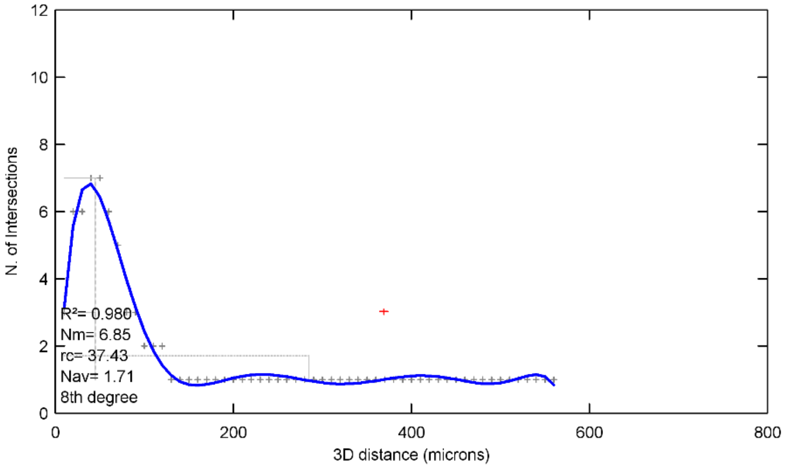

2
Delayed

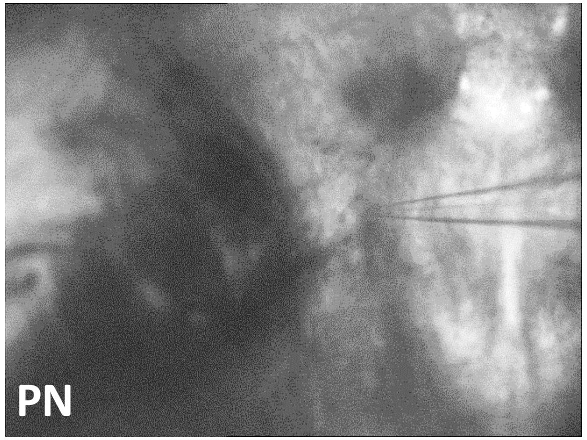

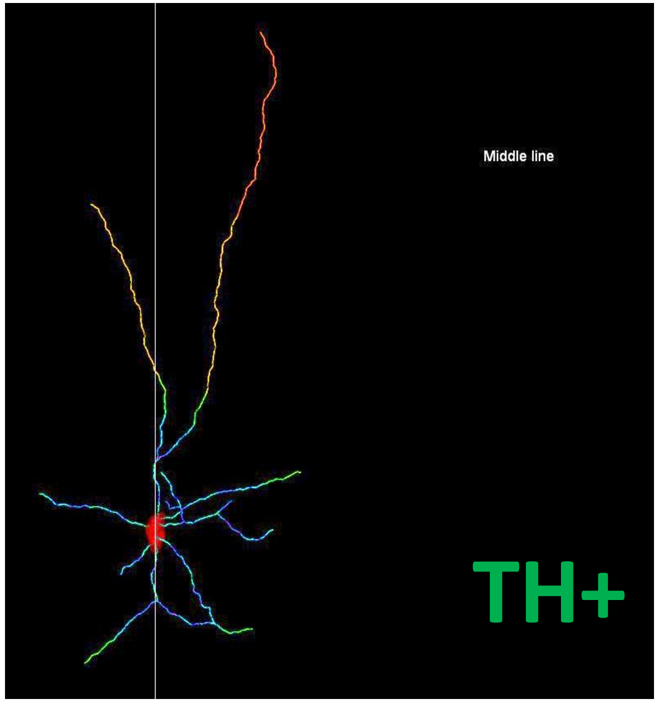

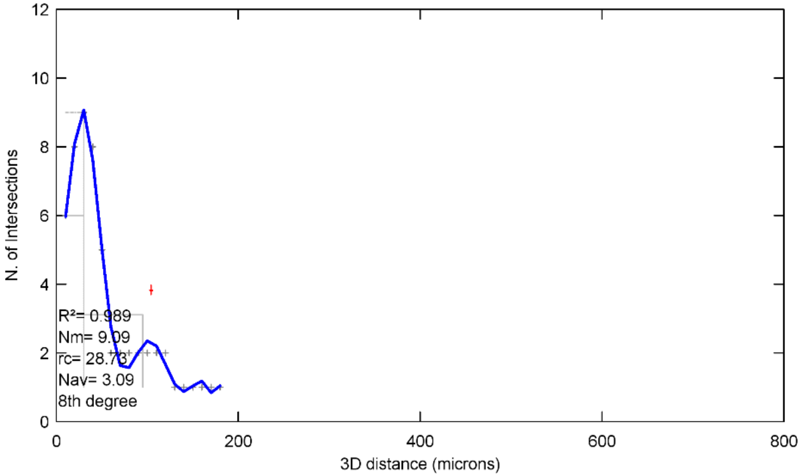

Neurobiotin

3
Delayed

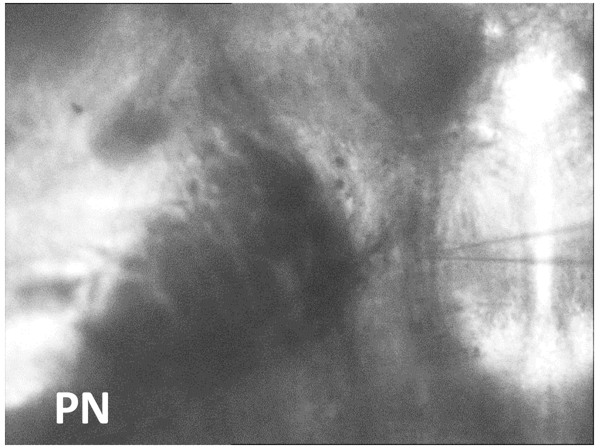

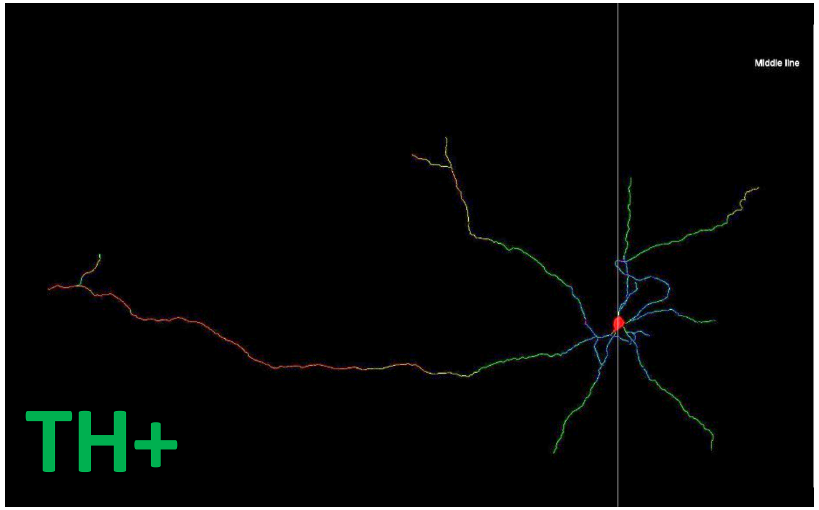

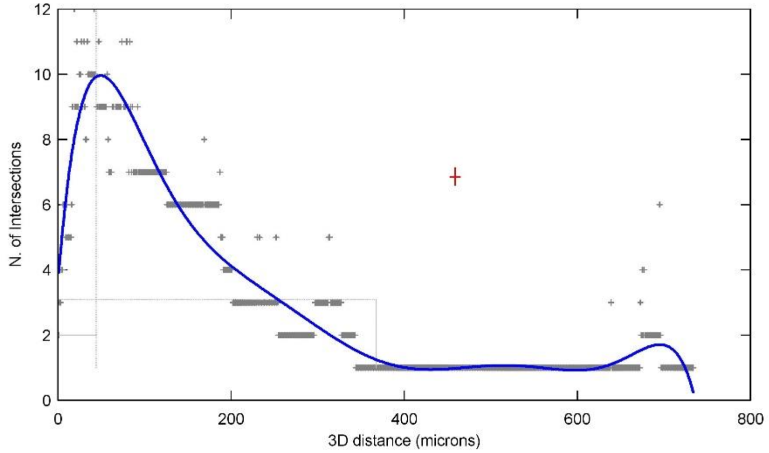

4
Delayed

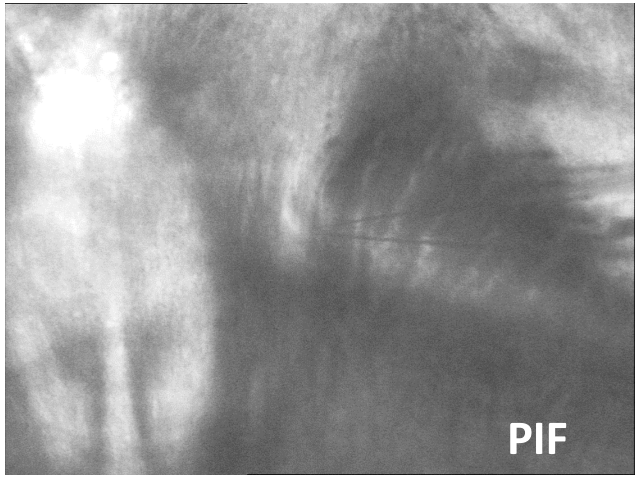

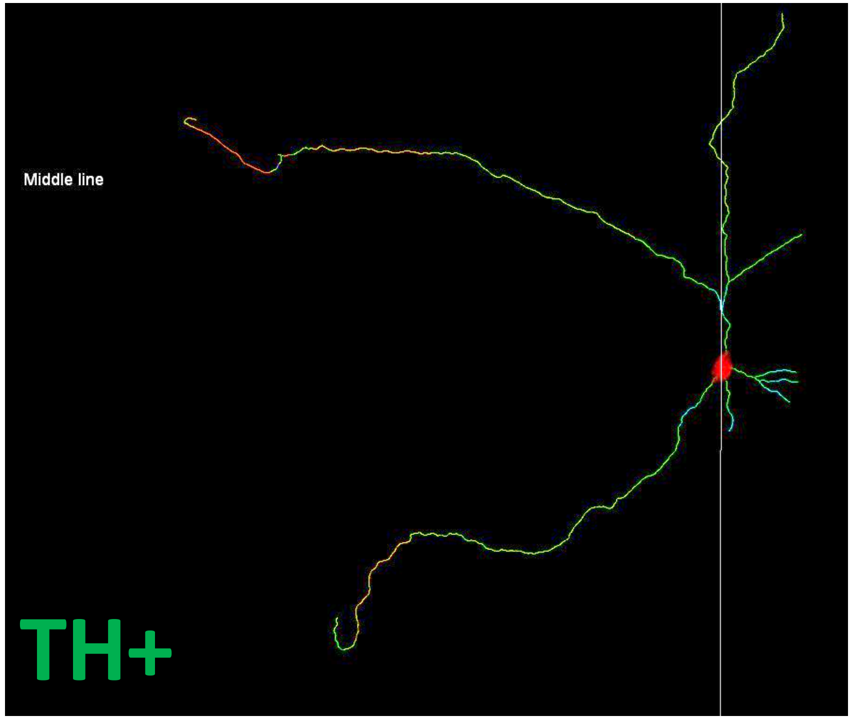

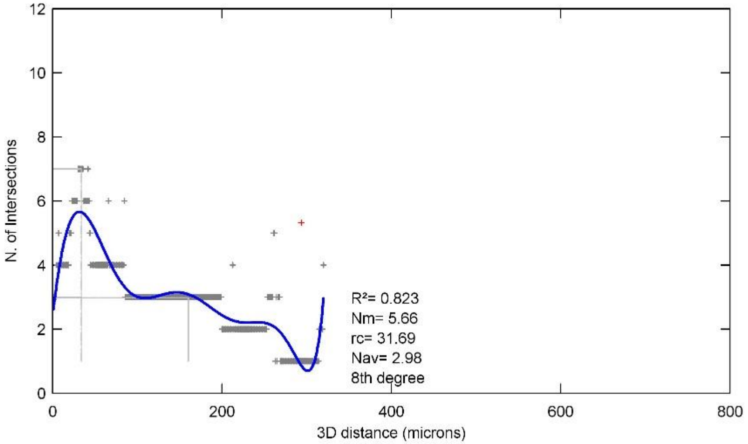

Neurobiotin

5
Delayed

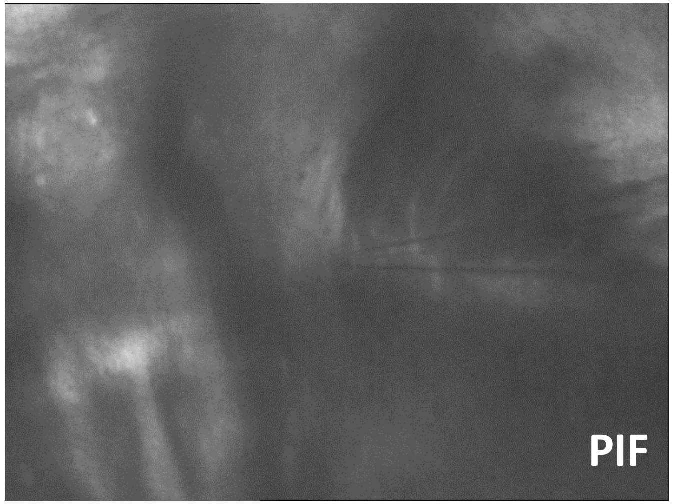

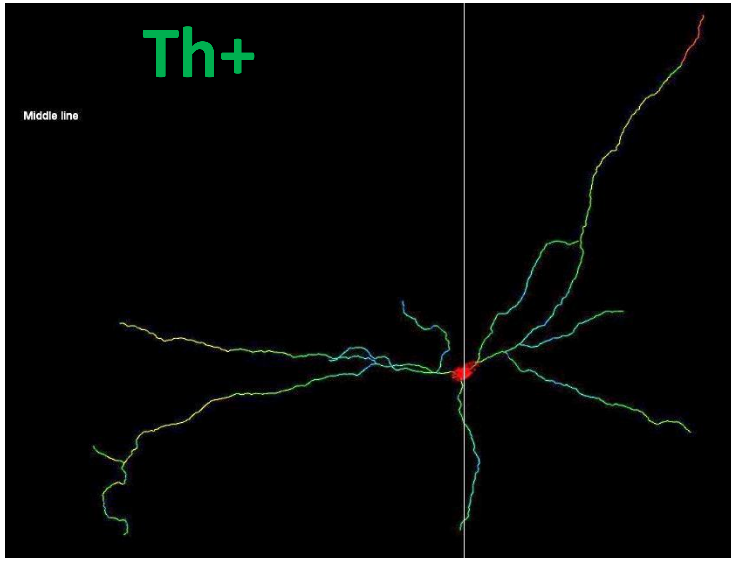

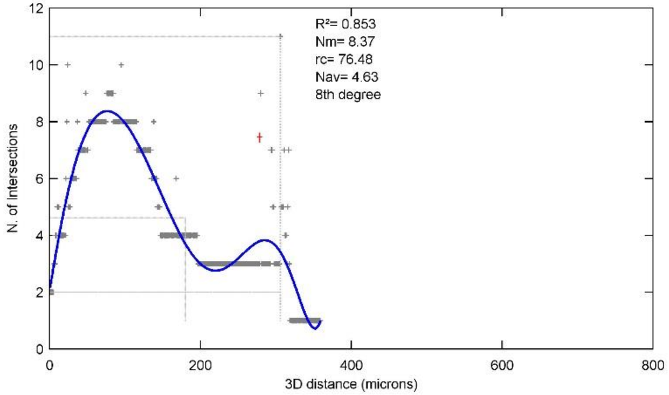

6
Delayed

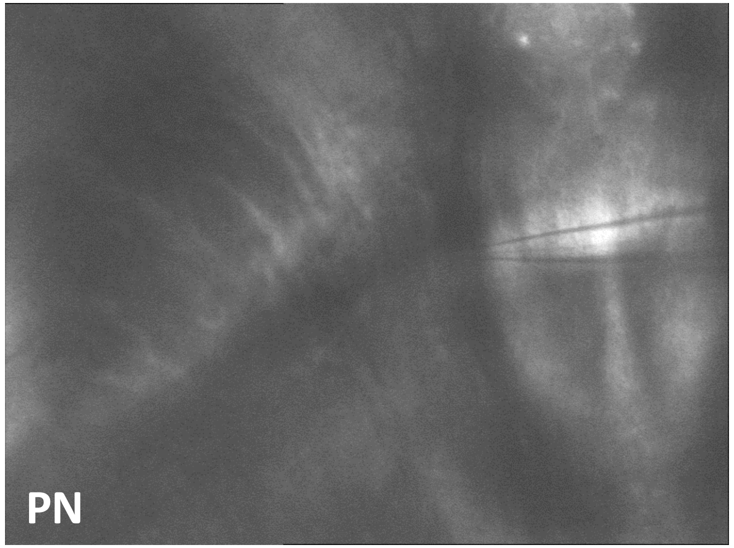

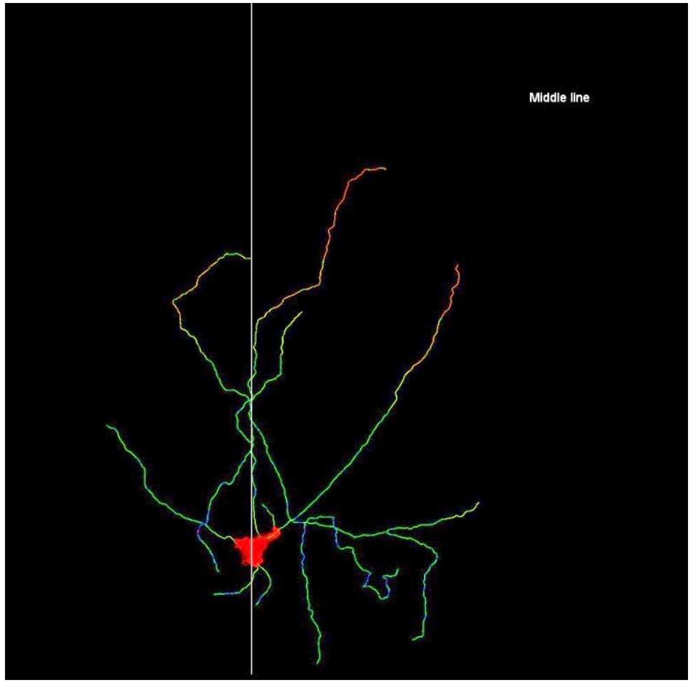

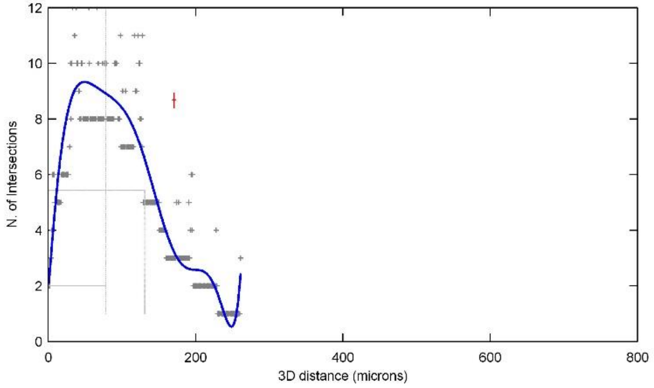

Neurobiotin

7
Delayed

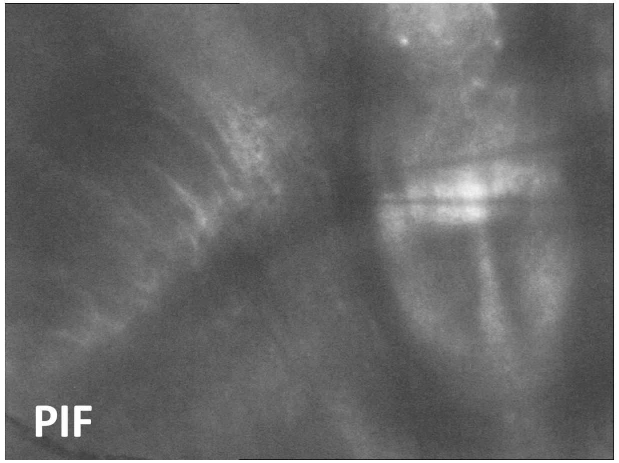

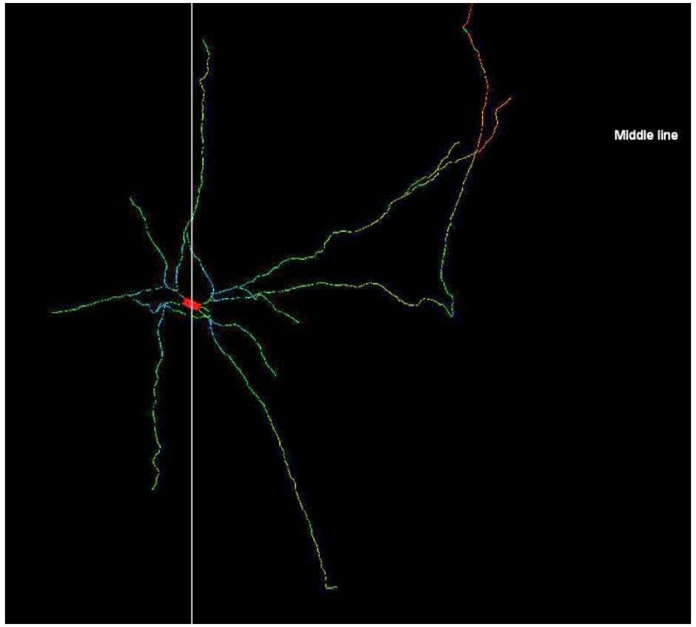

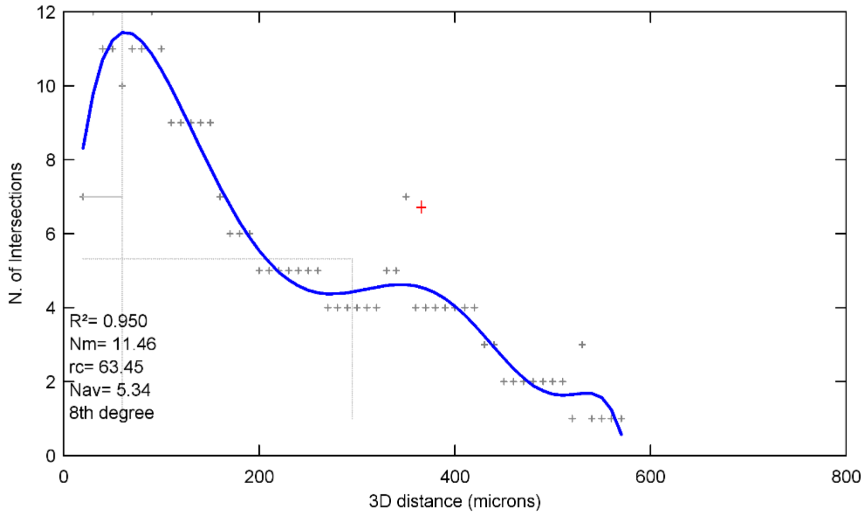

8
Delayed

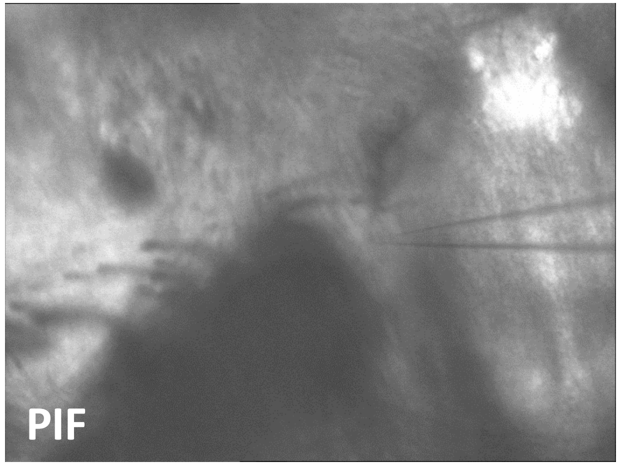

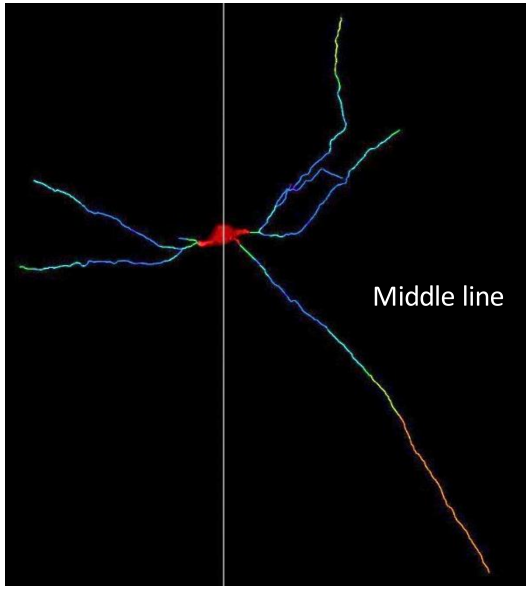

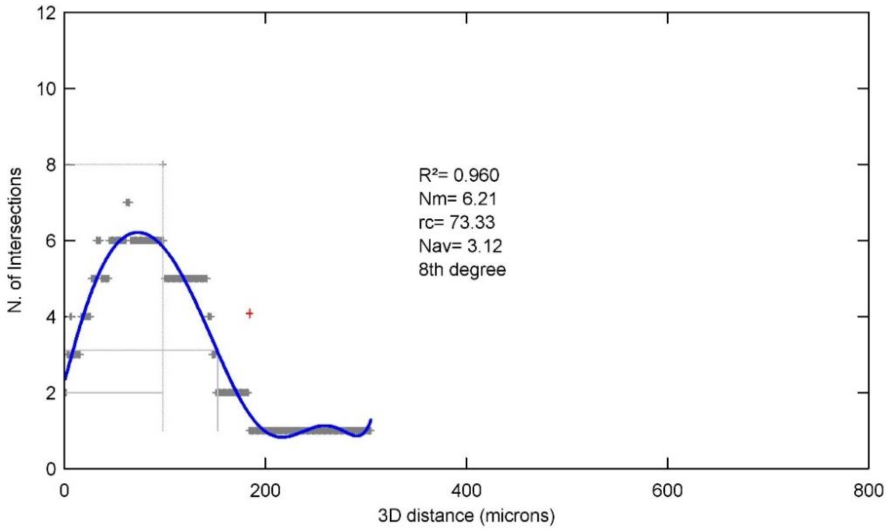

Biocytin

9
Delayed

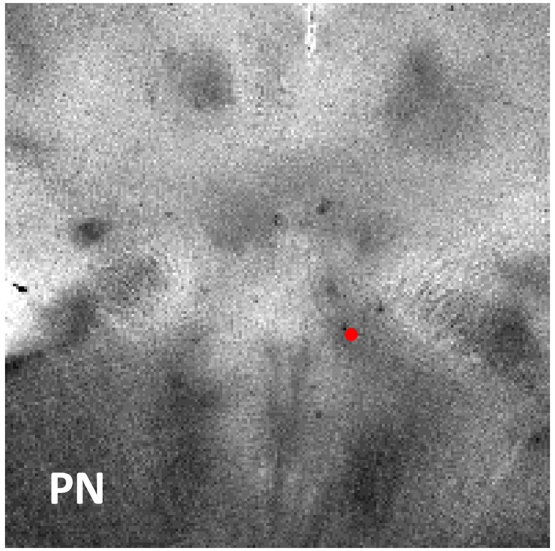

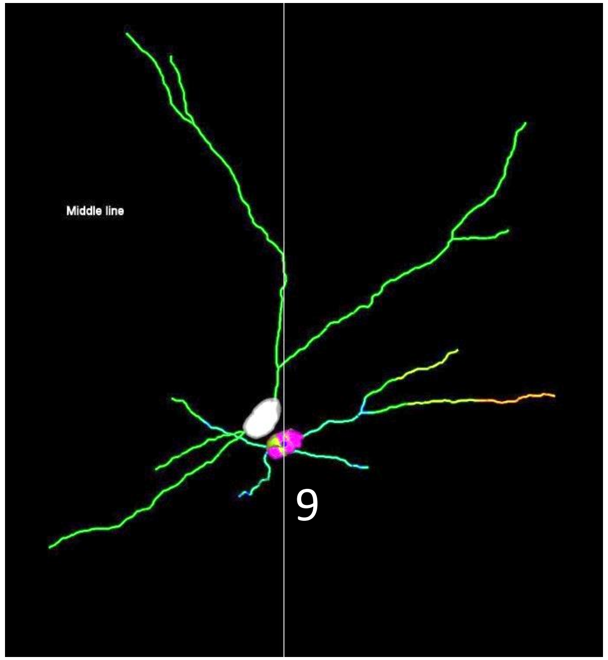

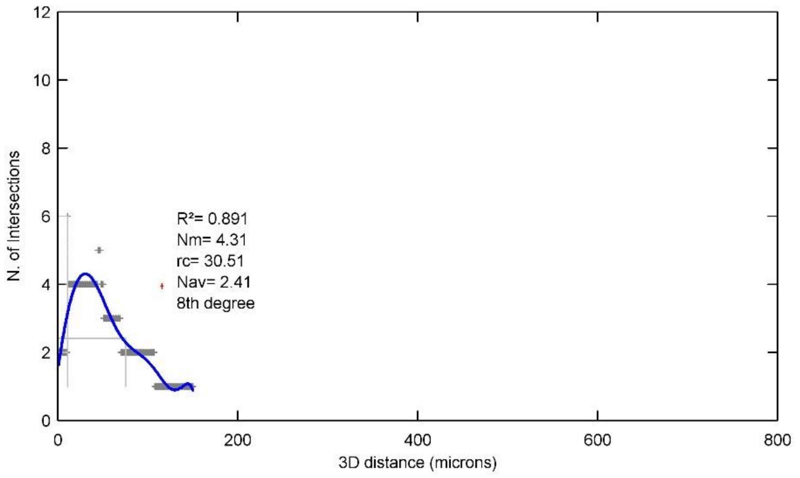

10
Delayed

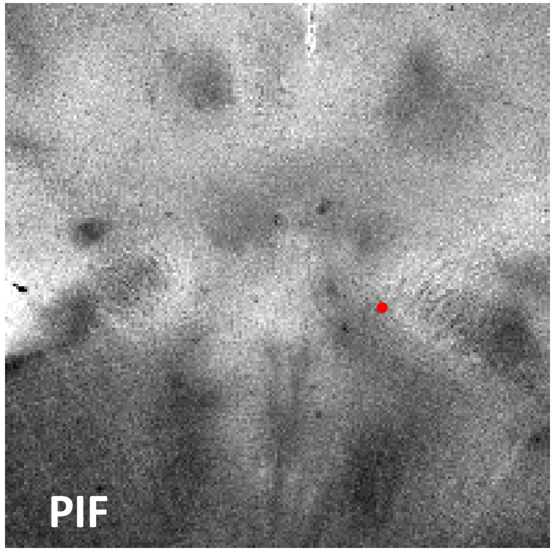

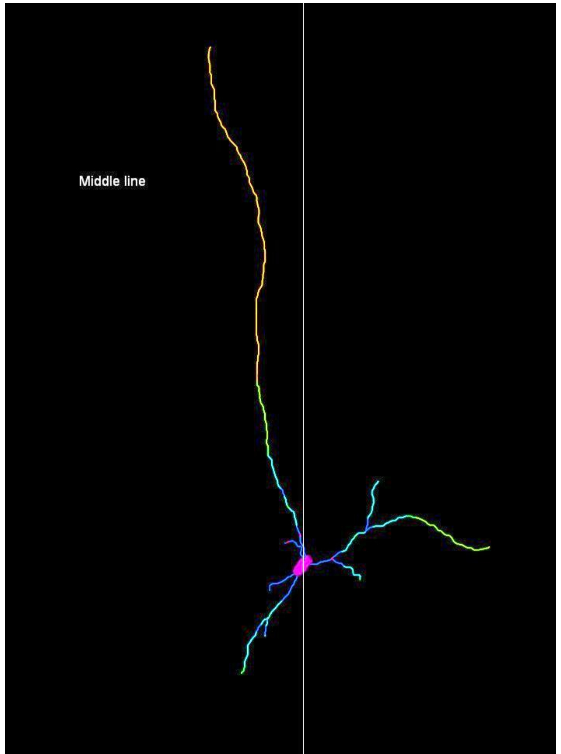

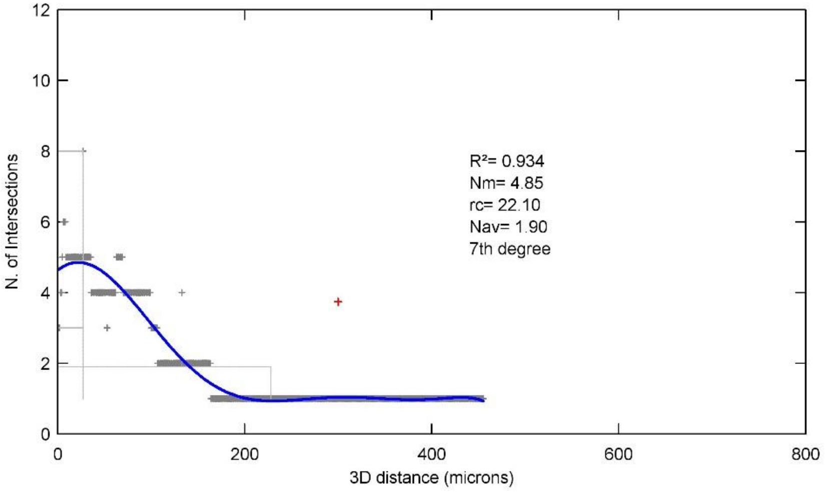

Biocytine

11, 12
Delayed

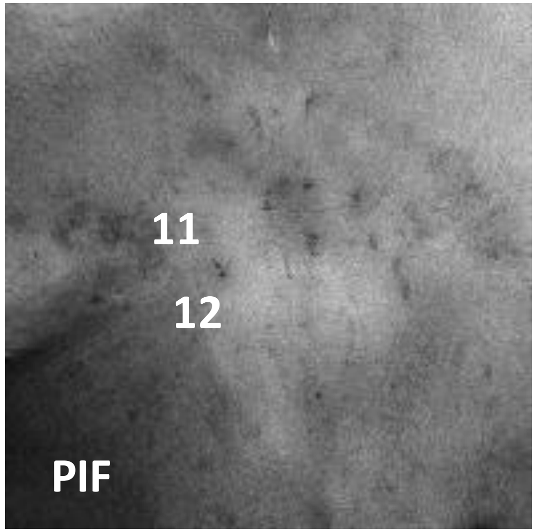

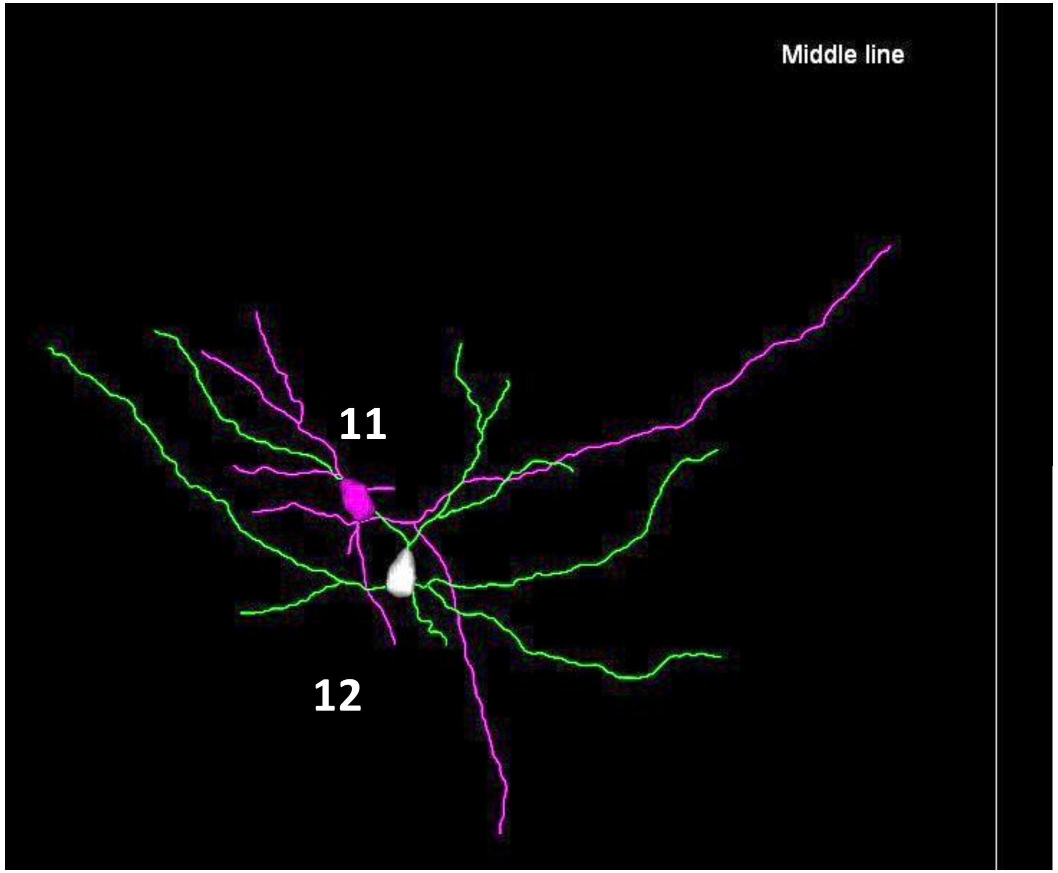

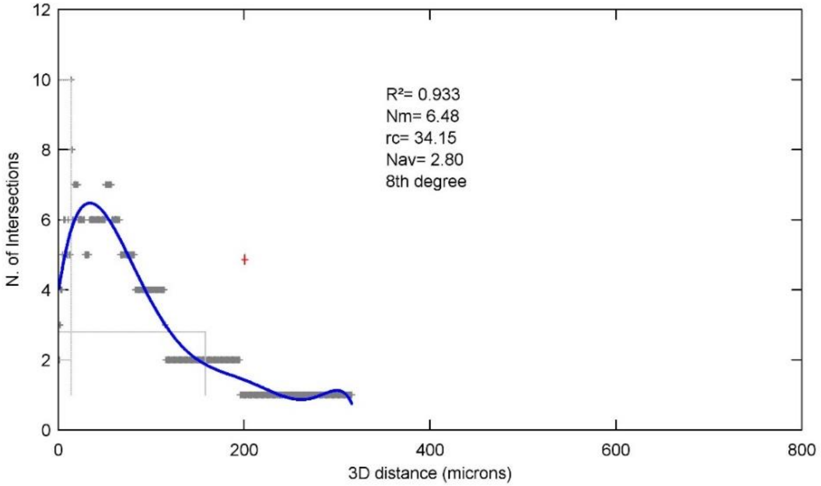

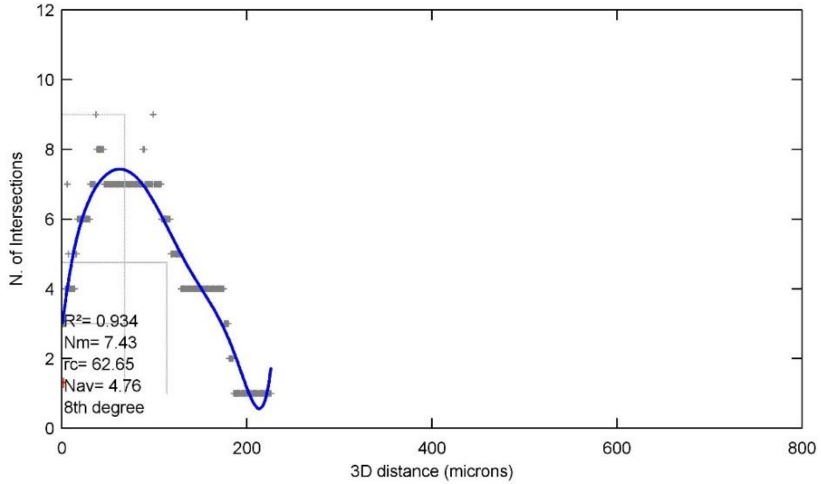

Biocytin

13
Delayed

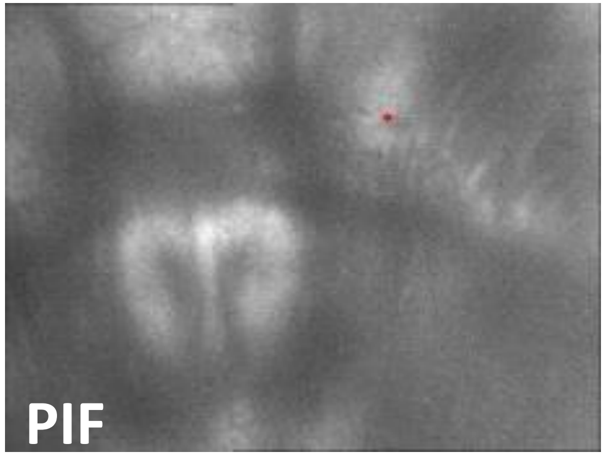

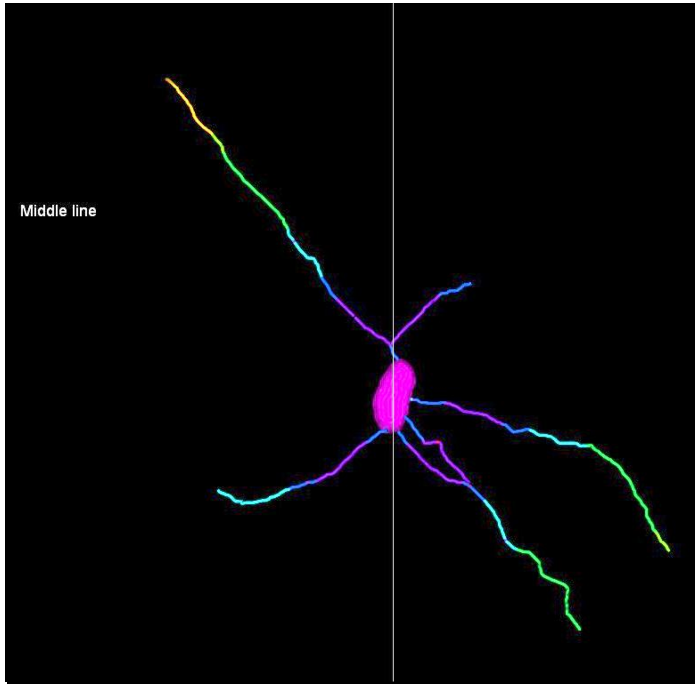

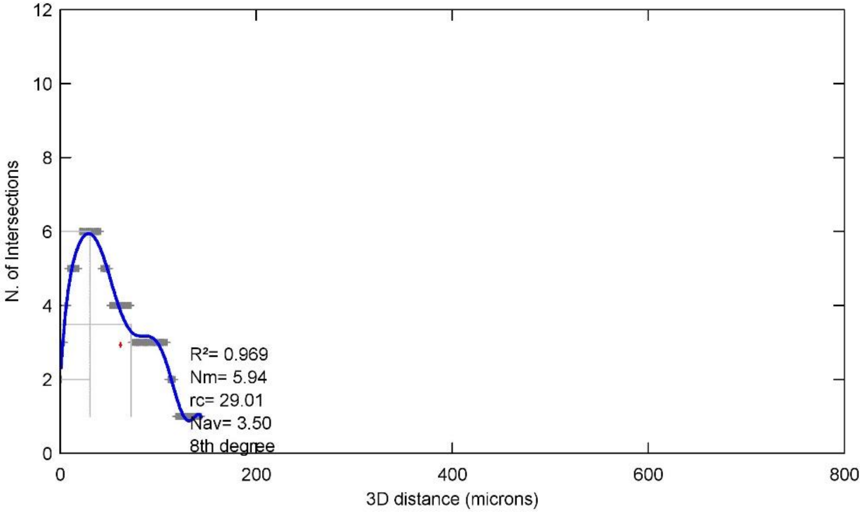

14
Delayed

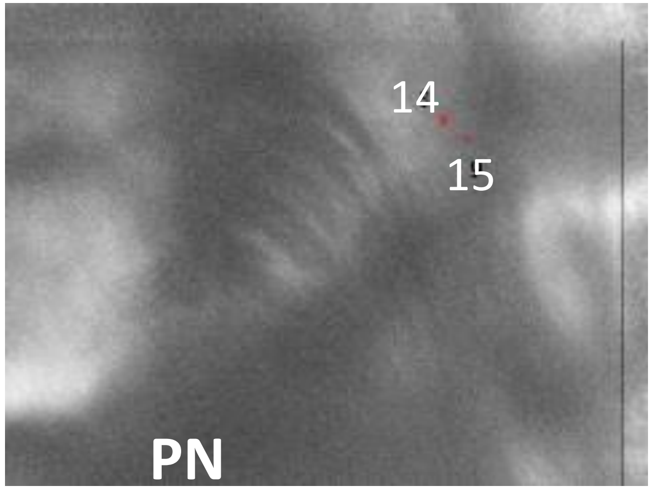

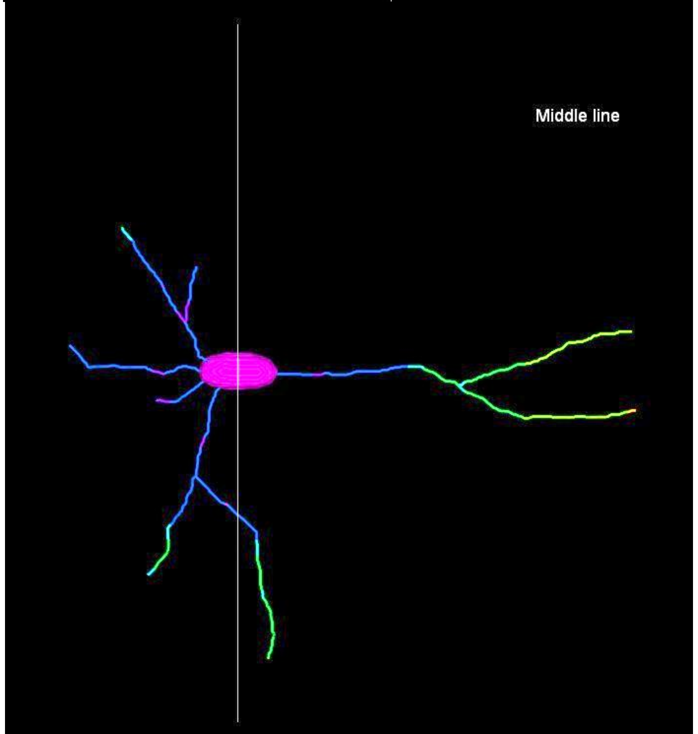

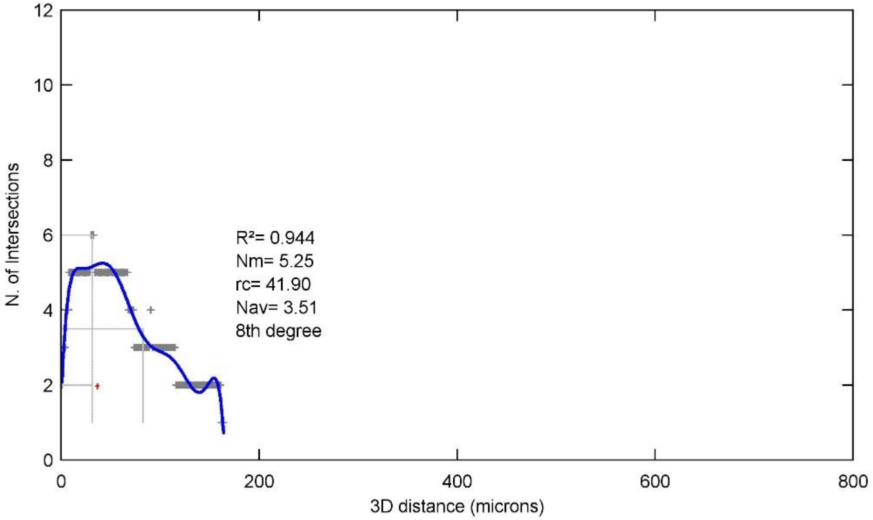

Biocytin

15
Delayed

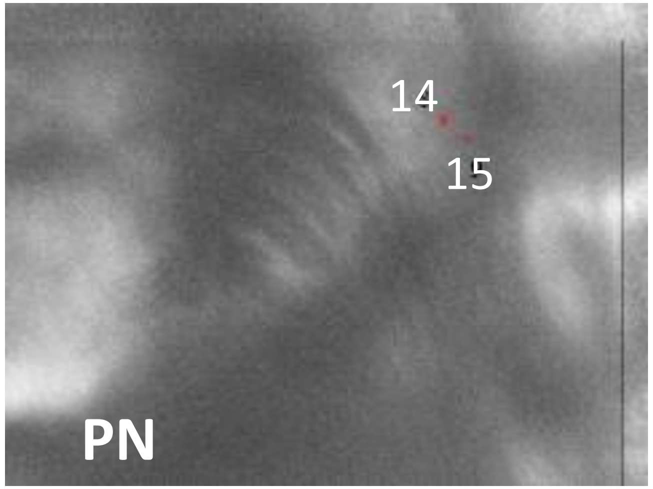

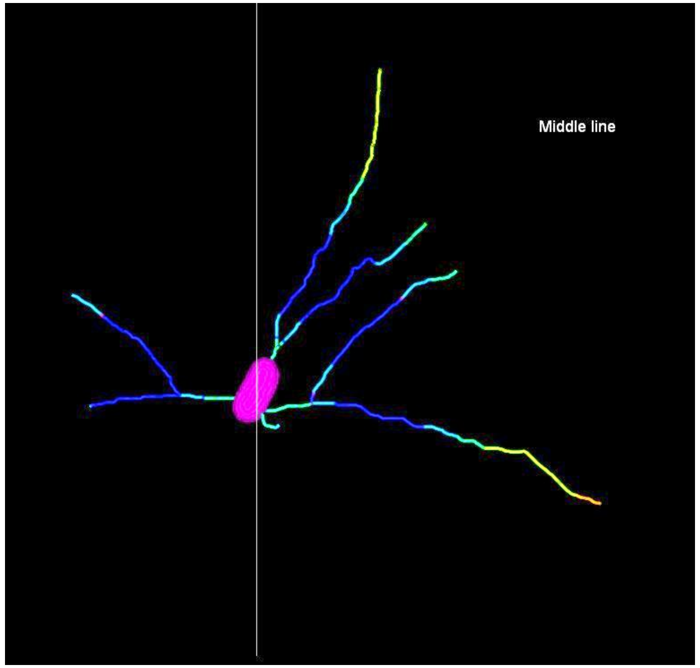

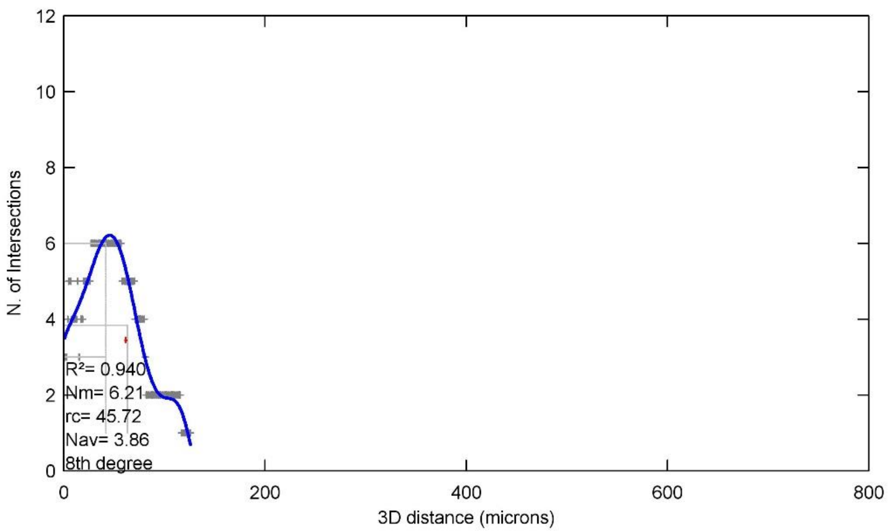

16
Delayed

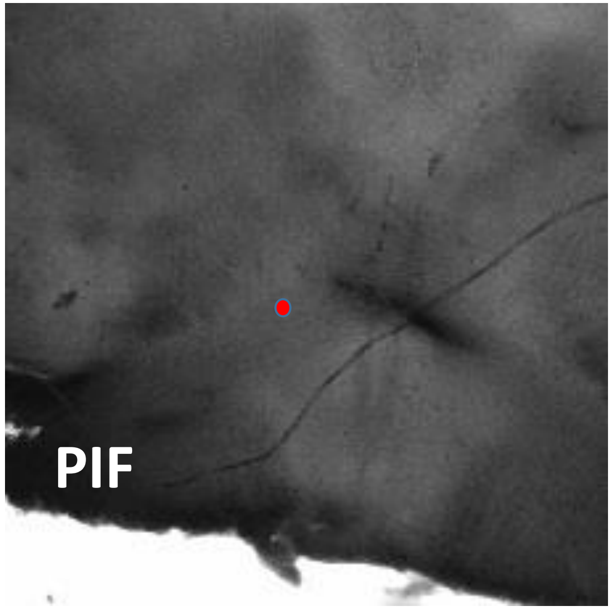

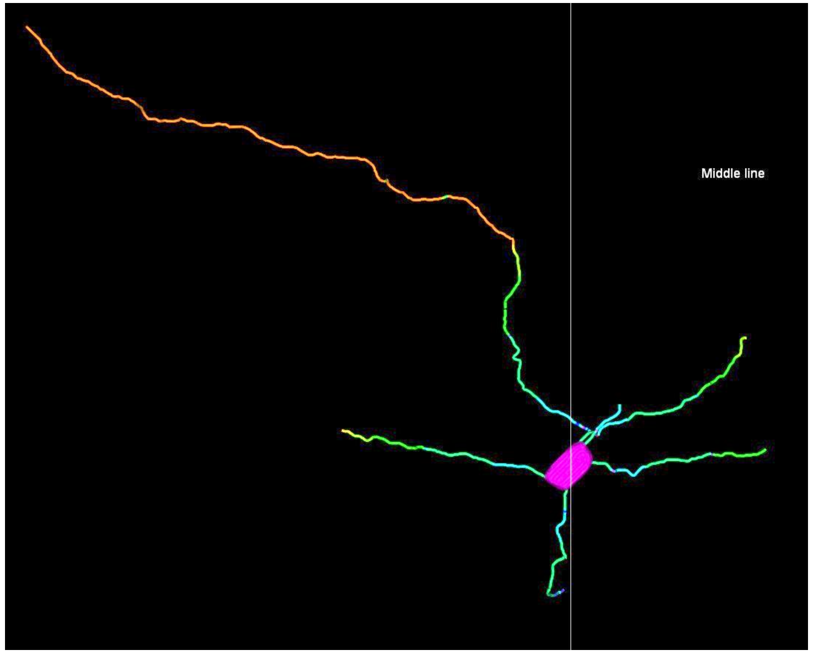

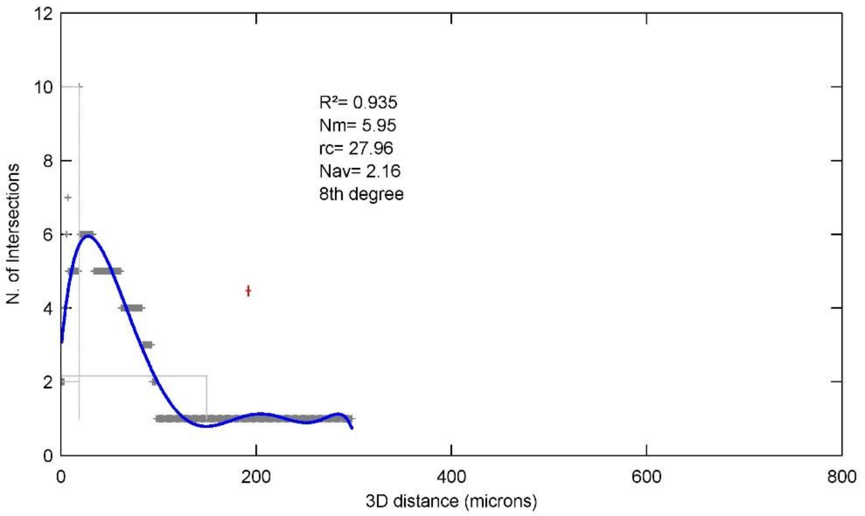

Biocytin

17
Delayed

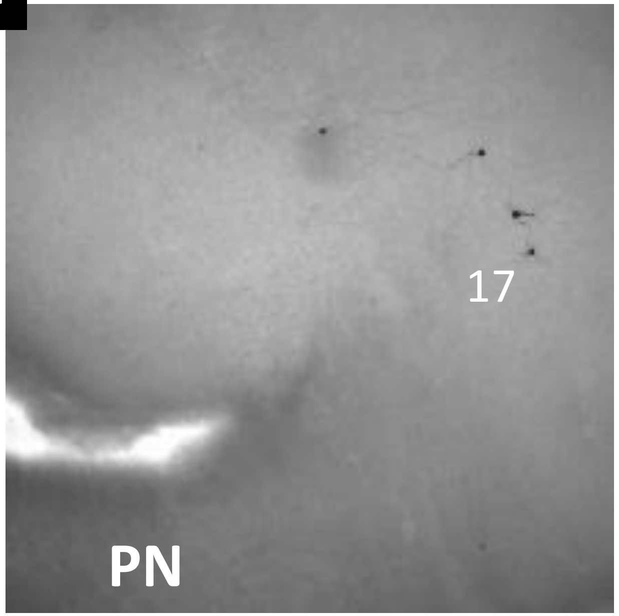

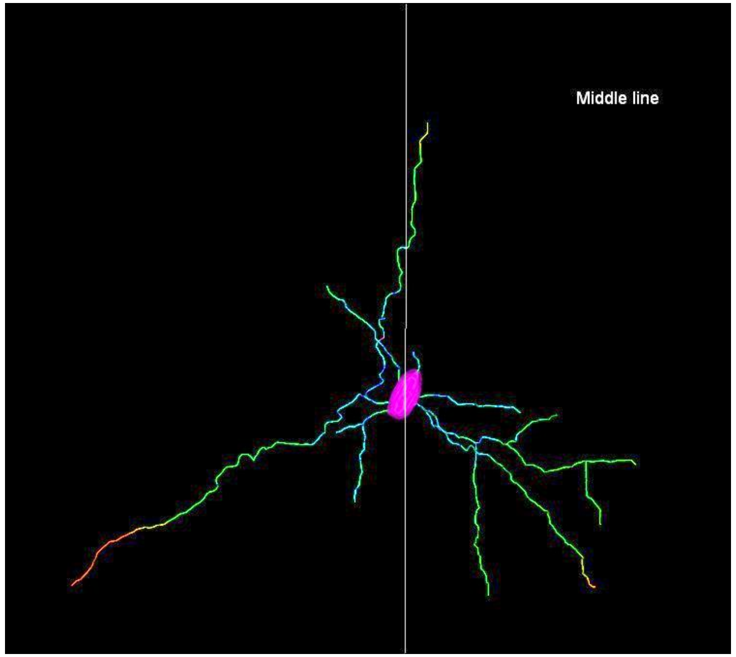

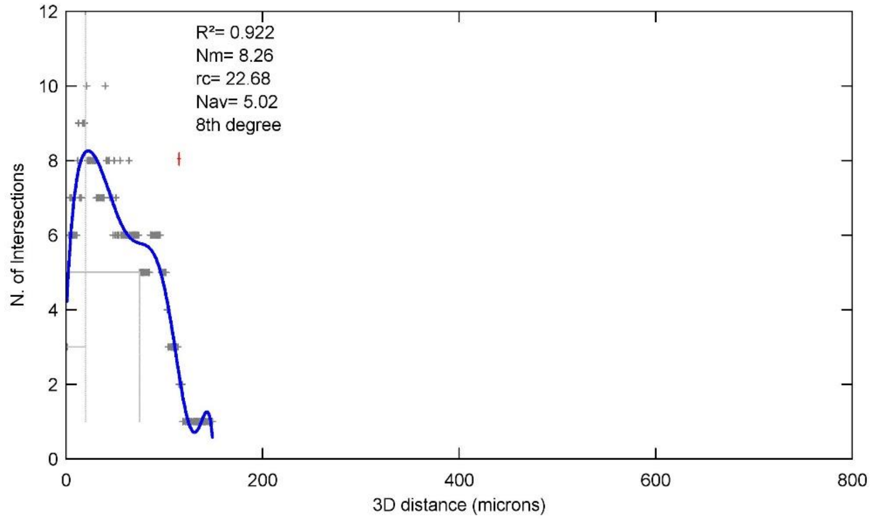

18
Delayed

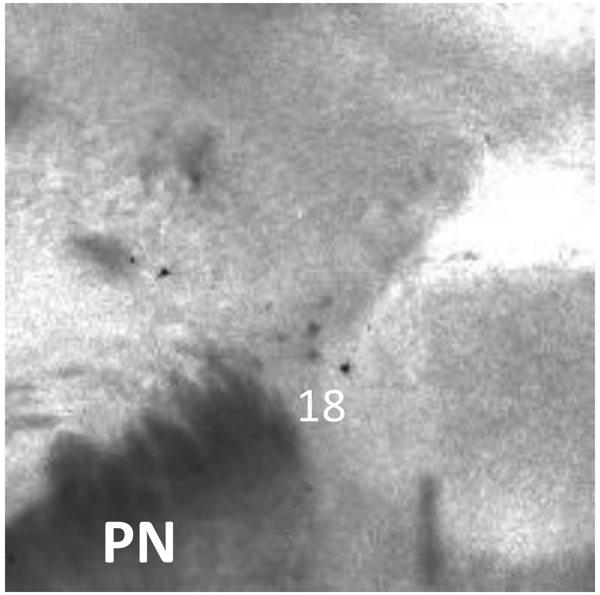

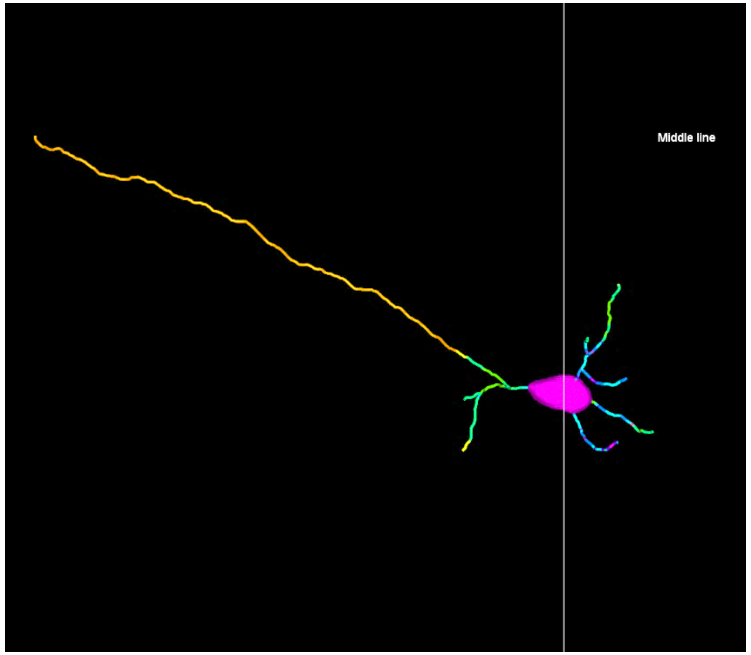

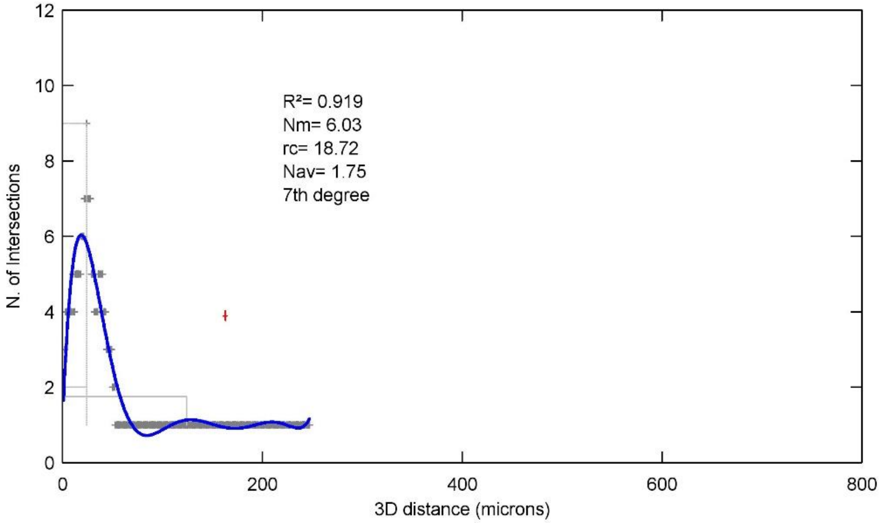

Supplement: Figure 5—source data 2. — This zip archive contains morphological images of all traced neurons grouped according their electrophysiological profiles. Each subtype’s folder contains a PDF file (with the list of neurons, their original location within the VTA, images of the traced morphology and individual Sholl curves) and two subfolders: ‘3D_gif’ – with *.gif files of the listed neurons; and ‘WaveFront_3D_obj’ – with corresponding *.obj files. *.gif files can be opened by any image viewer. *.obj files save information about the 3D model of the neurons and can be opened/reused with any 3D viewer or graphic software. [file elife-59328-fig5-data2.zip › Morphology_Source/Delayed/Delayed_list.pdf]
