# Supplementary figures and images for: Heterogeneous somatostatin-expressing neuron population in mouse ventral tegmental area
Source: eLife. 2020 Aug 4;9:e59328. doi: 10.7554/eLife.59328 (PMC7440918; doi:10.7554/eLife.59328)

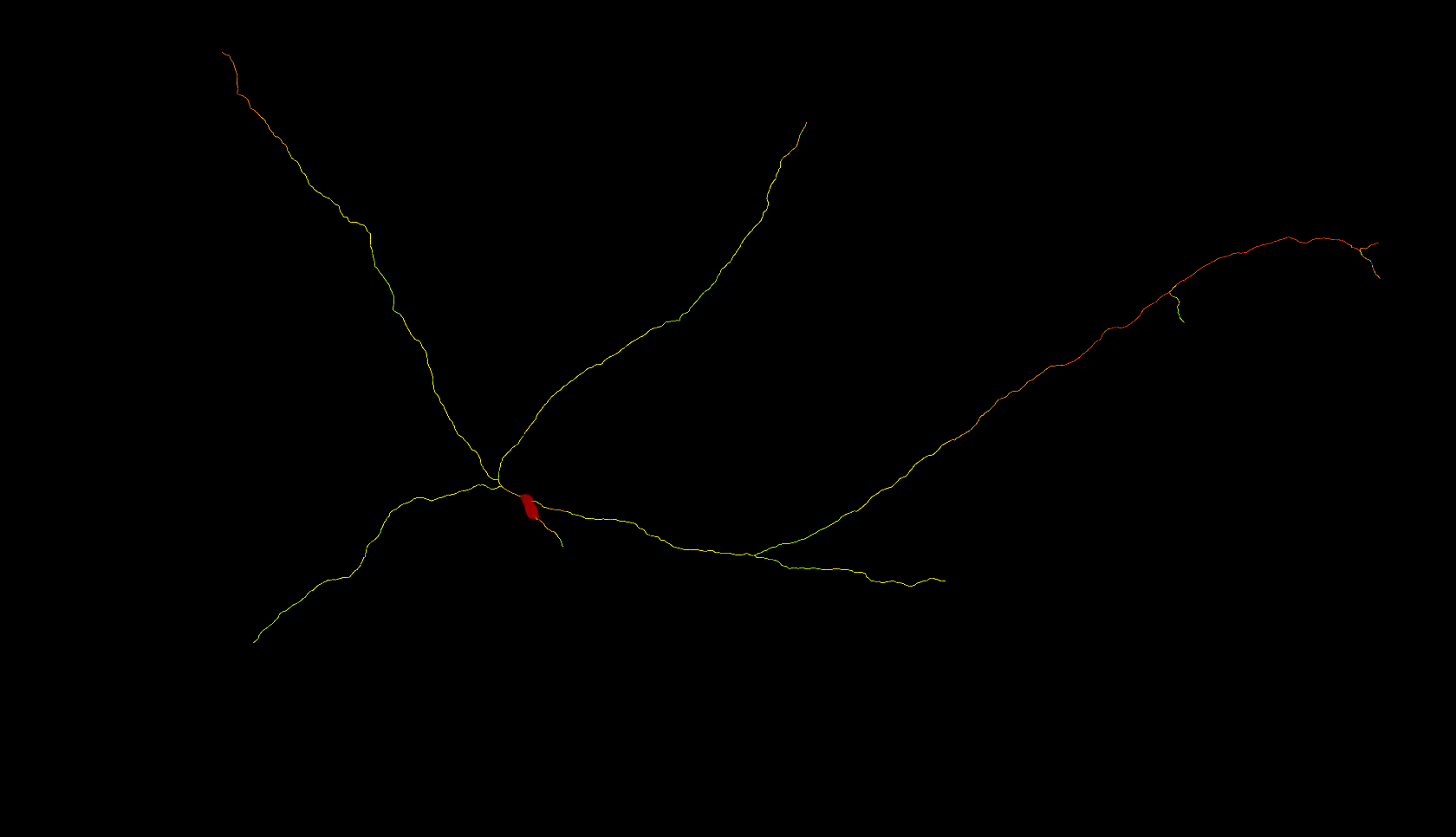

Supplement: Figure 5—source data 2. — This zip archive contains morphological images of all traced neurons grouped according their electrophysiological profiles. Each subtype’s folder contains a PDF file (with the list of neurons, their original location within the VTA, images of the traced morphology and individual Sholl curves) and two subfolders: ‘3D_gif’ – with *.gif files of the listed neurons; and ‘WaveFront_3D_obj’ – with corresponding *.obj files. *.gif files can be opened by any image viewer. *.obj files save information about the 3D model of the neurons and can be opened/reused with any 3D viewer or graphic software. [file elife-59328-fig5-data2.zip › Morphology_Source/ADP/3D_gif/1.gif]

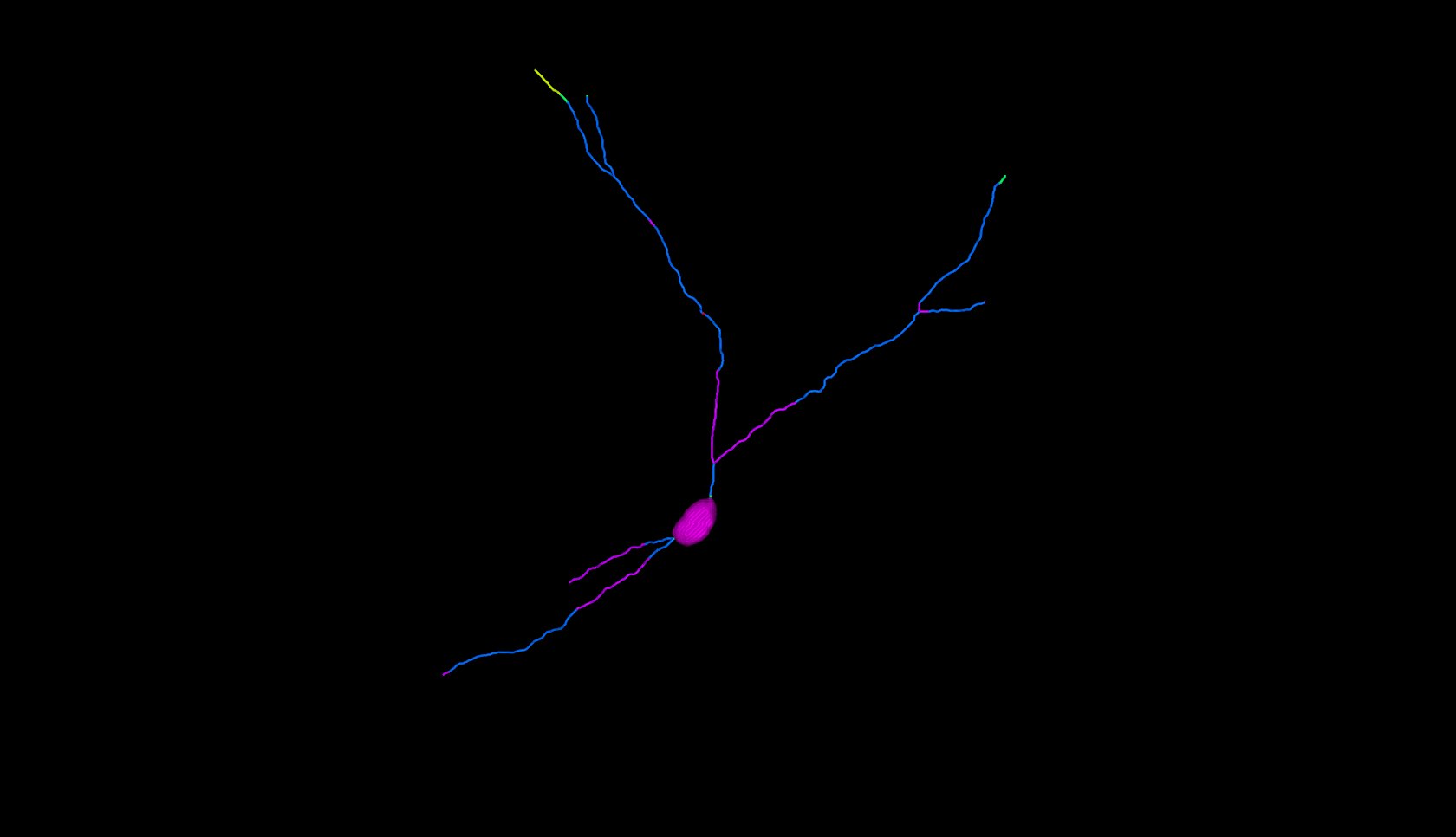

Supplement: Figure 5—source data 2. — This zip archive contains morphological images of all traced neurons grouped according their electrophysiological profiles. Each subtype’s folder contains a PDF file (with the list of neurons, their original location within the VTA, images of the traced morphology and individual Sholl curves) and two subfolders: ‘3D_gif’ – with *.gif files of the listed neurons; and ‘WaveFront_3D_obj’ – with corresponding *.obj files. *.gif files can be opened by any image viewer. *.obj files save information about the 3D model of the neurons and can be opened/reused with any 3D viewer or graphic software. [file elife-59328-fig5-data2.zip › Morphology_Source/ADP/3D_gif/10.gif]

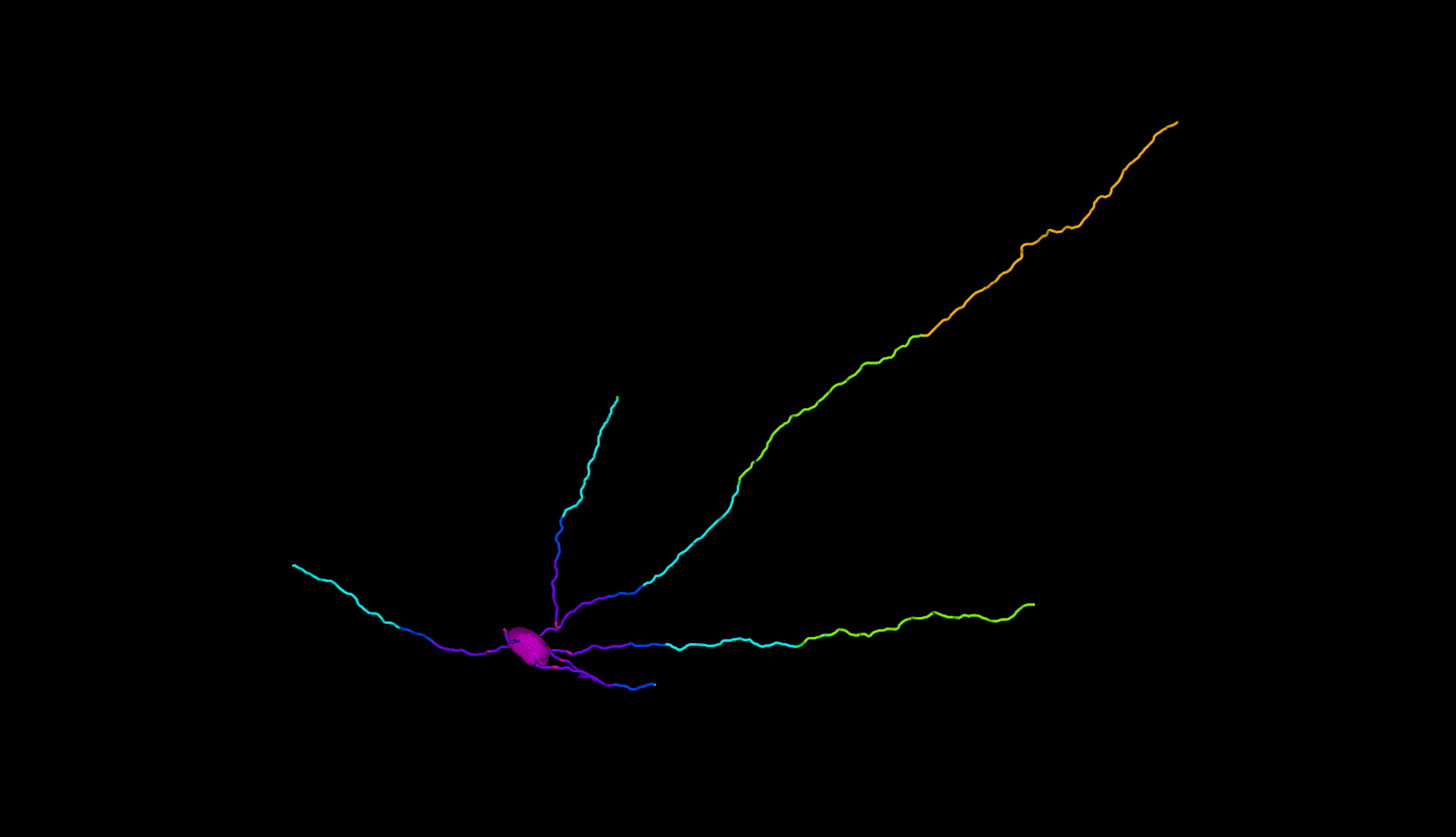

Supplement: Figure 5—source data 2. — This zip archive contains morphological images of all traced neurons grouped according their electrophysiological profiles. Each subtype’s folder contains a PDF file (with the list of neurons, their original location within the VTA, images of the traced morphology and individual Sholl curves) and two subfolders: ‘3D_gif’ – with *.gif files of the listed neurons; and ‘WaveFront_3D_obj’ – with corresponding *.obj files. *.gif files can be opened by any image viewer. *.obj files save information about the 3D model of the neurons and can be opened/reused with any 3D viewer or graphic software. [file elife-59328-fig5-data2.zip › Morphology_Source/ADP/3D_gif/12.gif]

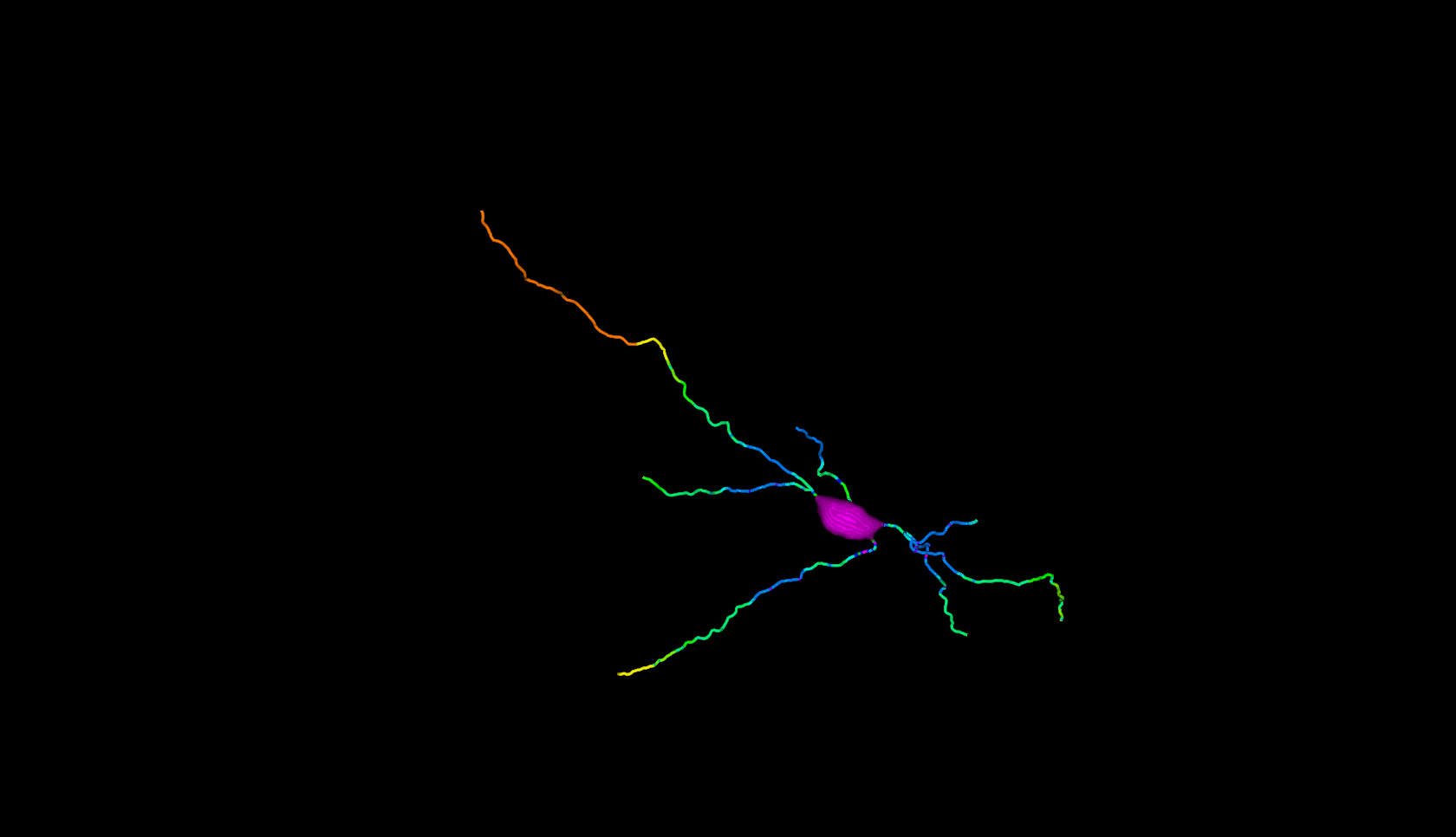

Supplement: Figure 5—source data 2. — This zip archive contains morphological images of all traced neurons grouped according their electrophysiological profiles. Each subtype’s folder contains a PDF file (with the list of neurons, their original location within the VTA, images of the traced morphology and individual Sholl curves) and two subfolders: ‘3D_gif’ – with *.gif files of the listed neurons; and ‘WaveFront_3D_obj’ – with corresponding *.obj files. *.gif files can be opened by any image viewer. *.obj files save information about the 3D model of the neurons and can be opened/reused with any 3D viewer or graphic software. [file elife-59328-fig5-data2.zip › Morphology_Source/ADP/3D_gif/13.gif]

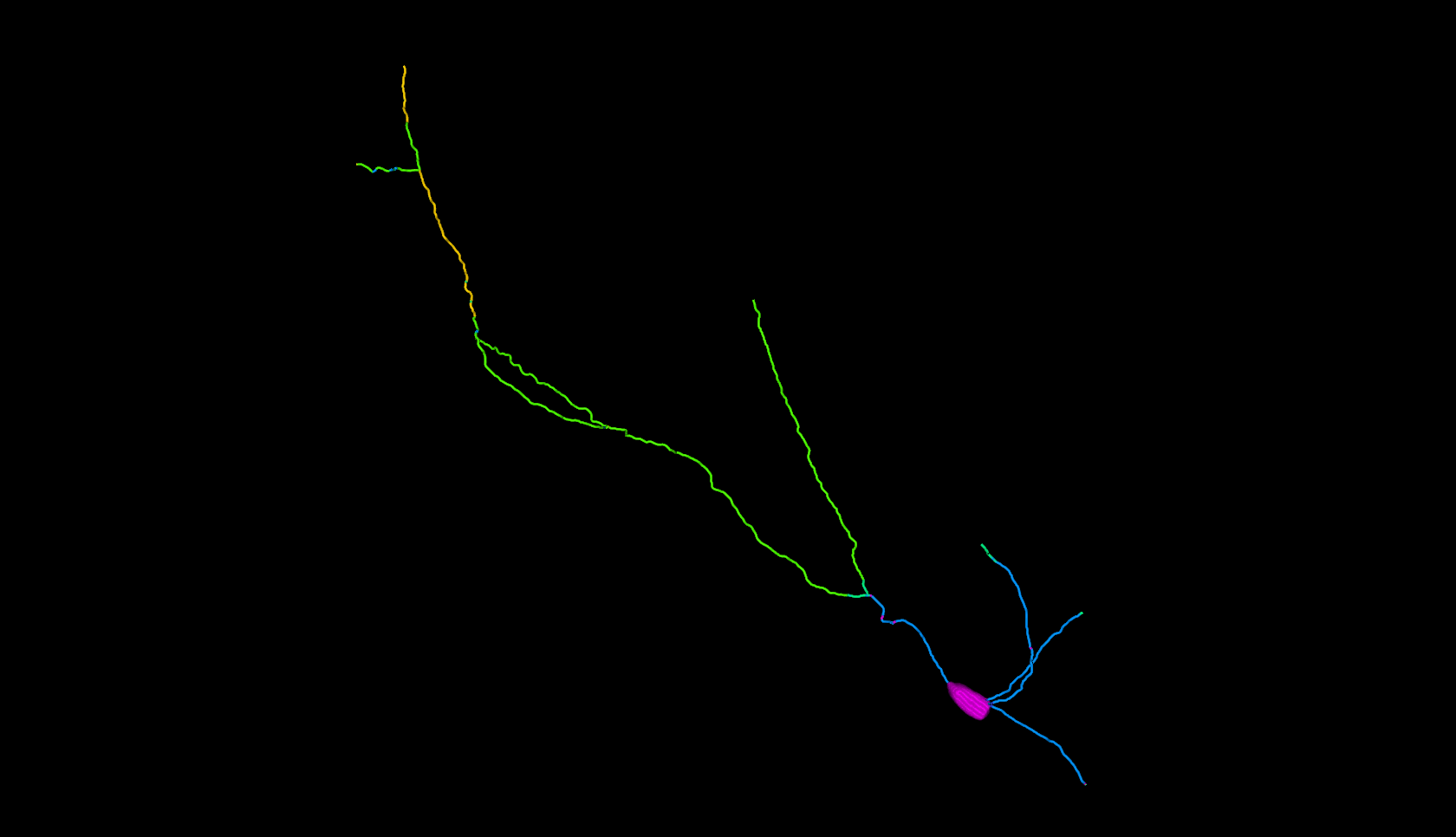

Supplement: Figure 5—source data 2. — This zip archive contains morphological images of all traced neurons grouped according their electrophysiological profiles. Each subtype’s folder contains a PDF file (with the list of neurons, their original location within the VTA, images of the traced morphology and individual Sholl curves) and two subfolders: ‘3D_gif’ – with *.gif files of the listed neurons; and ‘WaveFront_3D_obj’ – with corresponding *.obj files. *.gif files can be opened by any image viewer. *.obj files save information about the 3D model of the neurons and can be opened/reused with any 3D viewer or graphic software. [file elife-59328-fig5-data2.zip › Morphology_Source/ADP/3D_gif/14.gif]

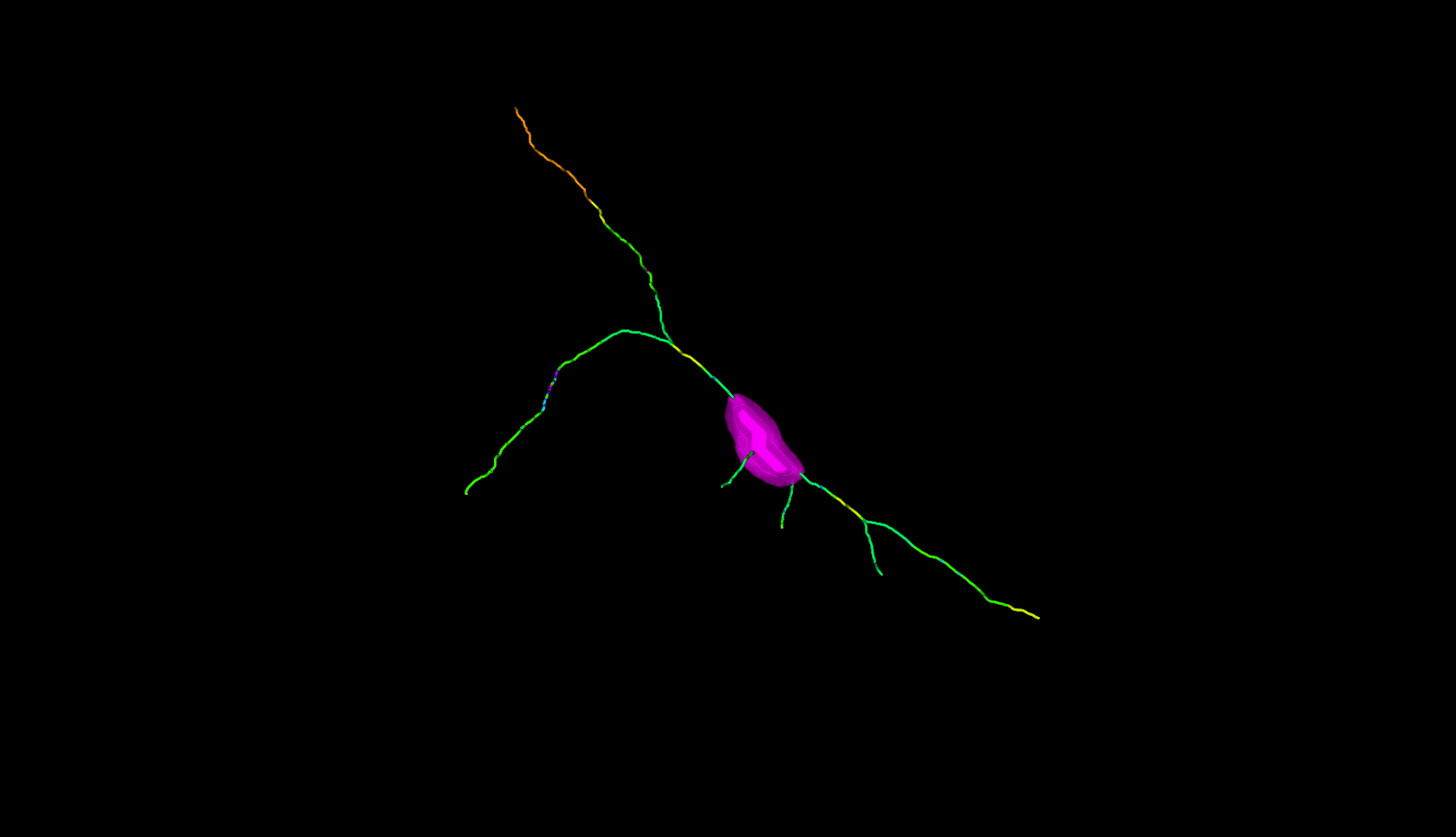

Supplement: Figure 5—source data 2. — This zip archive contains morphological images of all traced neurons grouped according their electrophysiological profiles. Each subtype’s folder contains a PDF file (with the list of neurons, their original location within the VTA, images of the traced morphology and individual Sholl curves) and two subfolders: ‘3D_gif’ – with *.gif files of the listed neurons; and ‘WaveFront_3D_obj’ – with corresponding *.obj files. *.gif files can be opened by any image viewer. *.obj files save information about the 3D model of the neurons and can be opened/reused with any 3D viewer or graphic software. [file elife-59328-fig5-data2.zip › Morphology_Source/ADP/3D_gif/15.gif]

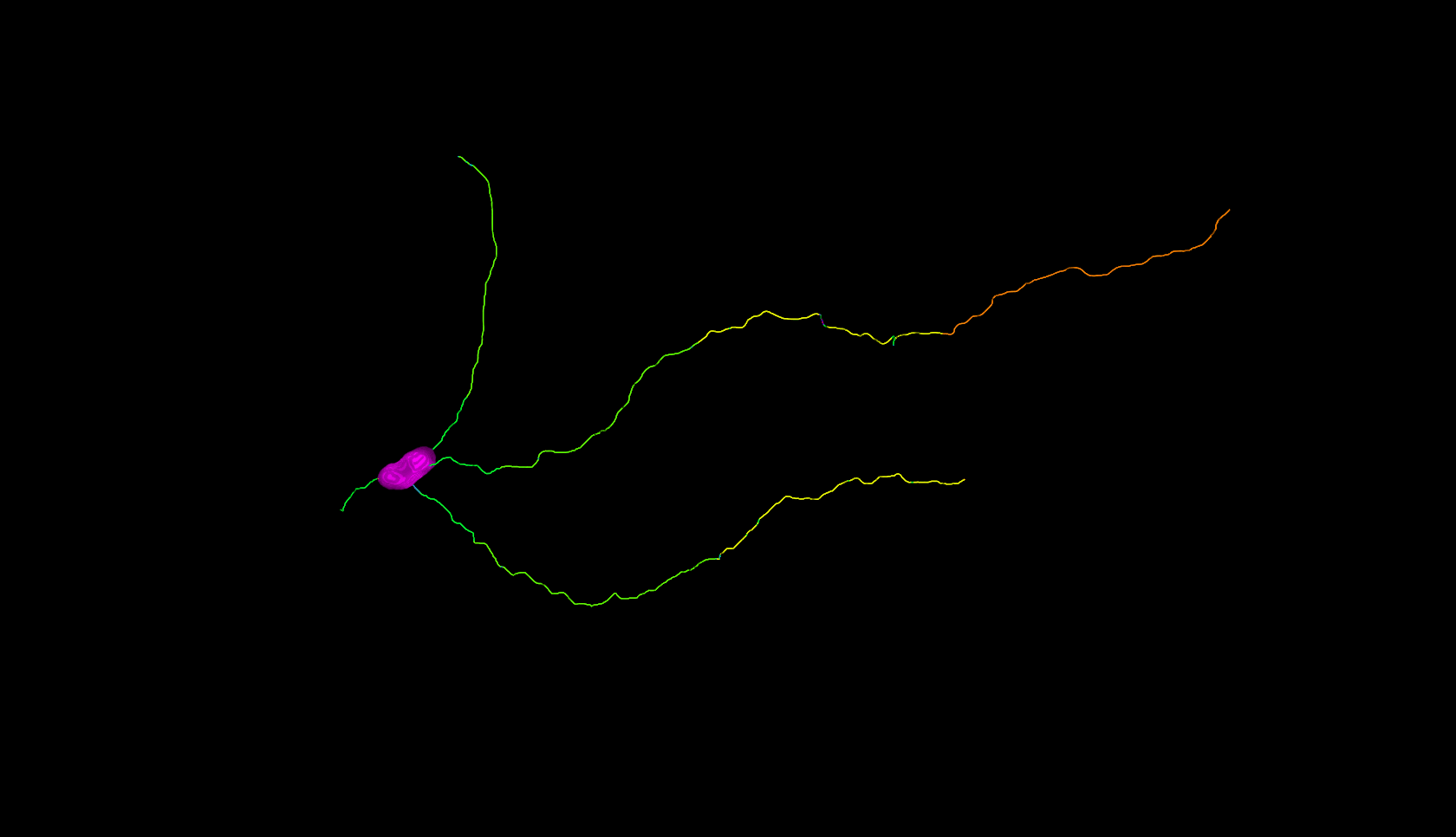

Supplement: Figure 5—source data 2. — This zip archive contains morphological images of all traced neurons grouped according their electrophysiological profiles. Each subtype’s folder contains a PDF file (with the list of neurons, their original location within the VTA, images of the traced morphology and individual Sholl curves) and two subfolders: ‘3D_gif’ – with *.gif files of the listed neurons; and ‘WaveFront_3D_obj’ – with corresponding *.obj files. *.gif files can be opened by any image viewer. *.obj files save information about the 3D model of the neurons and can be opened/reused with any 3D viewer or graphic software. [file elife-59328-fig5-data2.zip › Morphology_Source/ADP/3D_gif/16.gif]

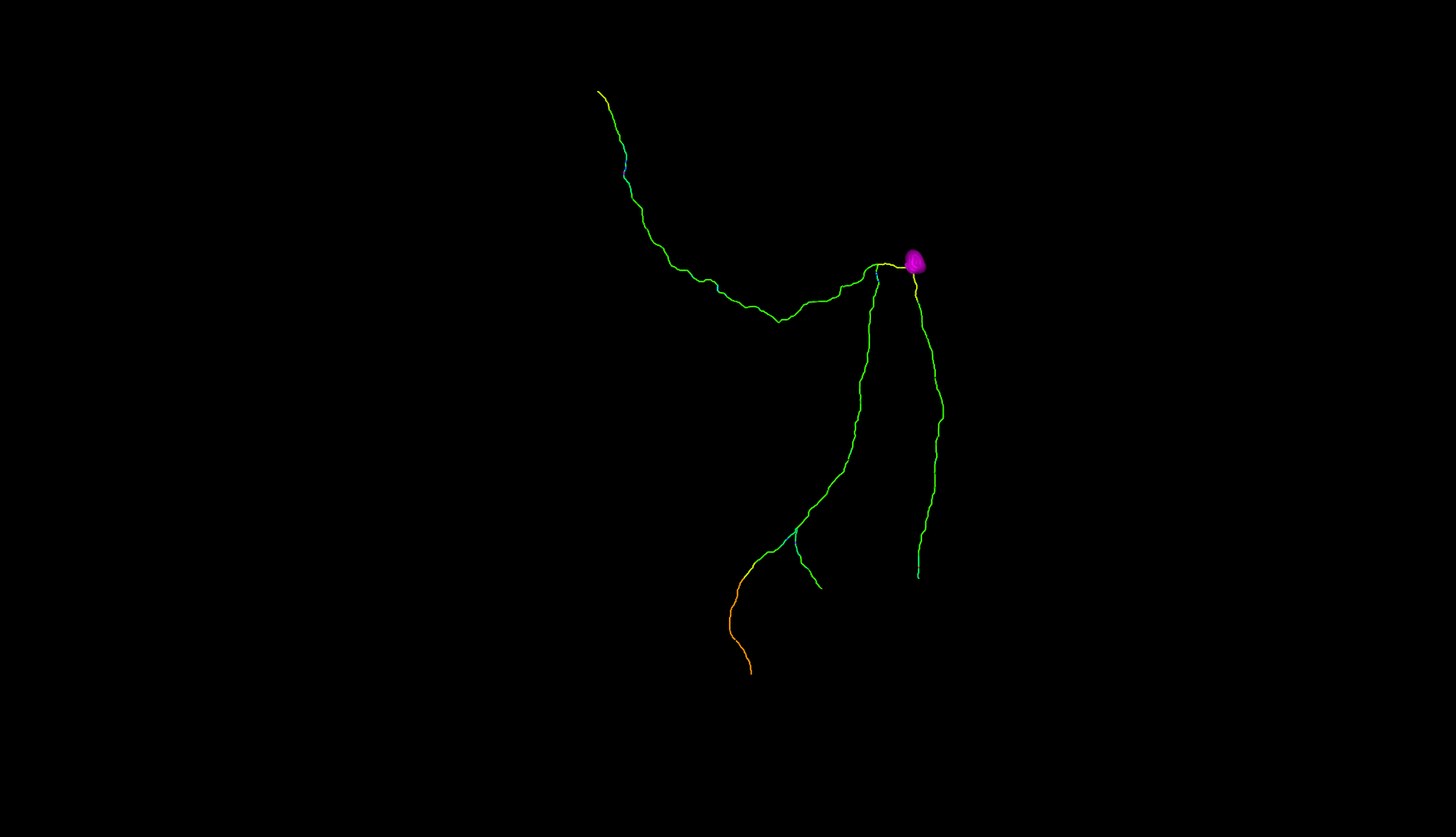

Supplement: Figure 5—source data 2. — This zip archive contains morphological images of all traced neurons grouped according their electrophysiological profiles. Each subtype’s folder contains a PDF file (with the list of neurons, their original location within the VTA, images of the traced morphology and individual Sholl curves) and two subfolders: ‘3D_gif’ – with *.gif files of the listed neurons; and ‘WaveFront_3D_obj’ – with corresponding *.obj files. *.gif files can be opened by any image viewer. *.obj files save information about the 3D model of the neurons and can be opened/reused with any 3D viewer or graphic software. [file elife-59328-fig5-data2.zip › Morphology_Source/ADP/3D_gif/17.gif]

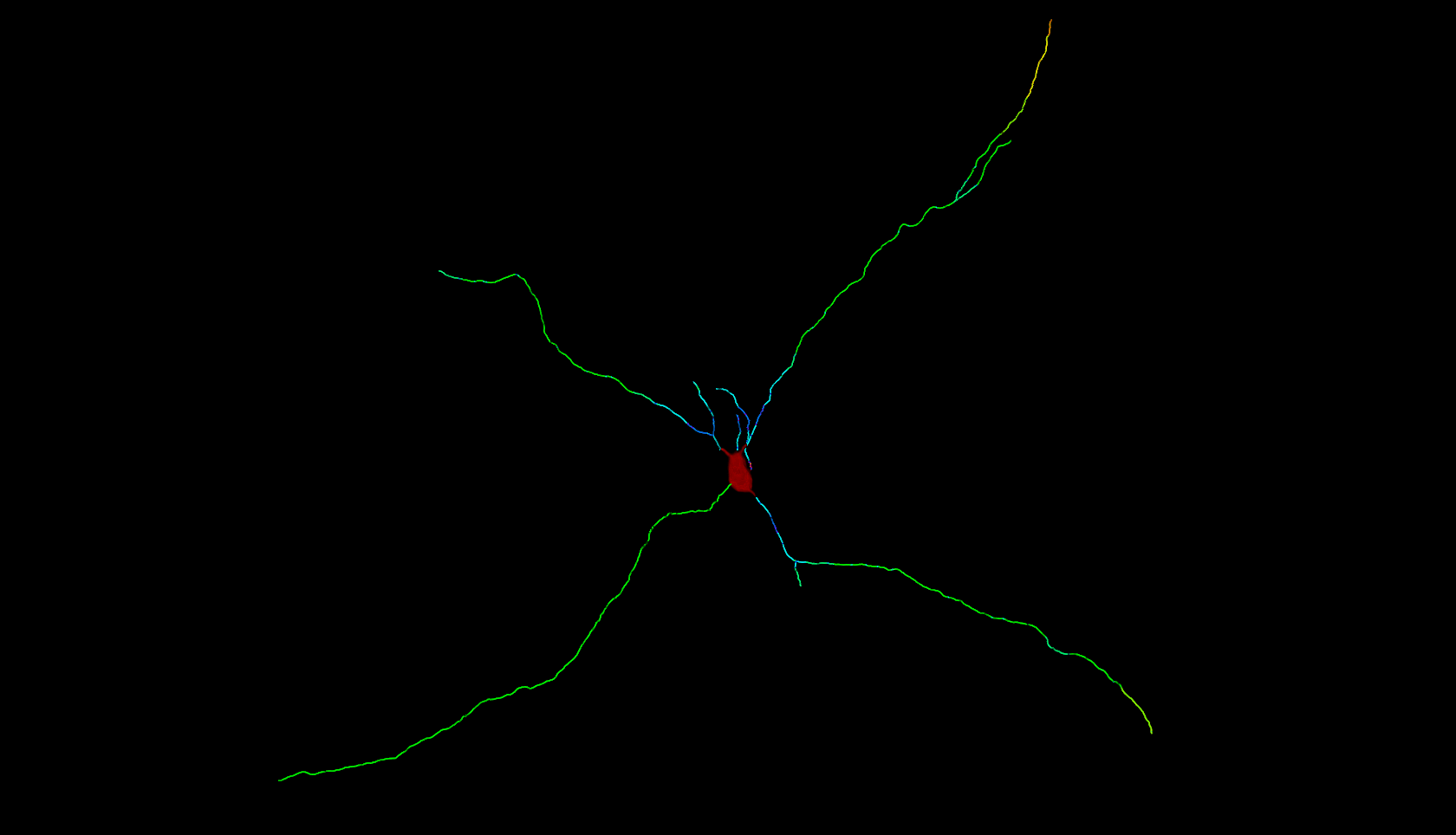

Supplement: Figure 5—source data 2. — This zip archive contains morphological images of all traced neurons grouped according their electrophysiological profiles. Each subtype’s folder contains a PDF file (with the list of neurons, their original location within the VTA, images of the traced morphology and individual Sholl curves) and two subfolders: ‘3D_gif’ – with *.gif files of the listed neurons; and ‘WaveFront_3D_obj’ – with corresponding *.obj files. *.gif files can be opened by any image viewer. *.obj files save information about the 3D model of the neurons and can be opened/reused with any 3D viewer or graphic software. [file elife-59328-fig5-data2.zip › Morphology_Source/ADP/3D_gif/2.gif]

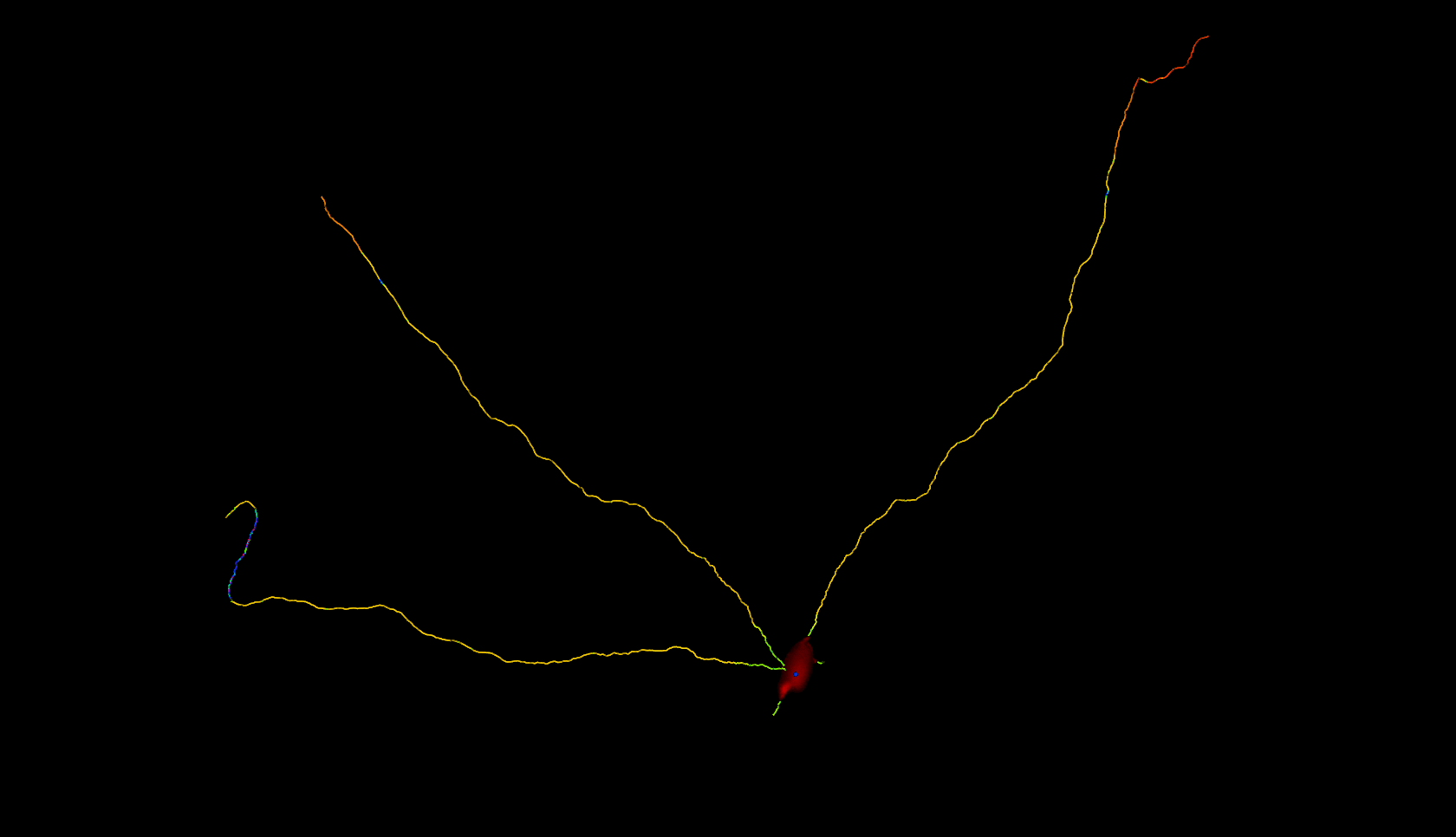

Supplement: Figure 5—source data 2. — This zip archive contains morphological images of all traced neurons grouped according their electrophysiological profiles. Each subtype’s folder contains a PDF file (with the list of neurons, their original location within the VTA, images of the traced morphology and individual Sholl curves) and two subfolders: ‘3D_gif’ – with *.gif files of the listed neurons; and ‘WaveFront_3D_obj’ – with corresponding *.obj files. *.gif files can be opened by any image viewer. *.obj files save information about the 3D model of the neurons and can be opened/reused with any 3D viewer or graphic software. [file elife-59328-fig5-data2.zip › Morphology_Source/ADP/3D_gif/3.gif]

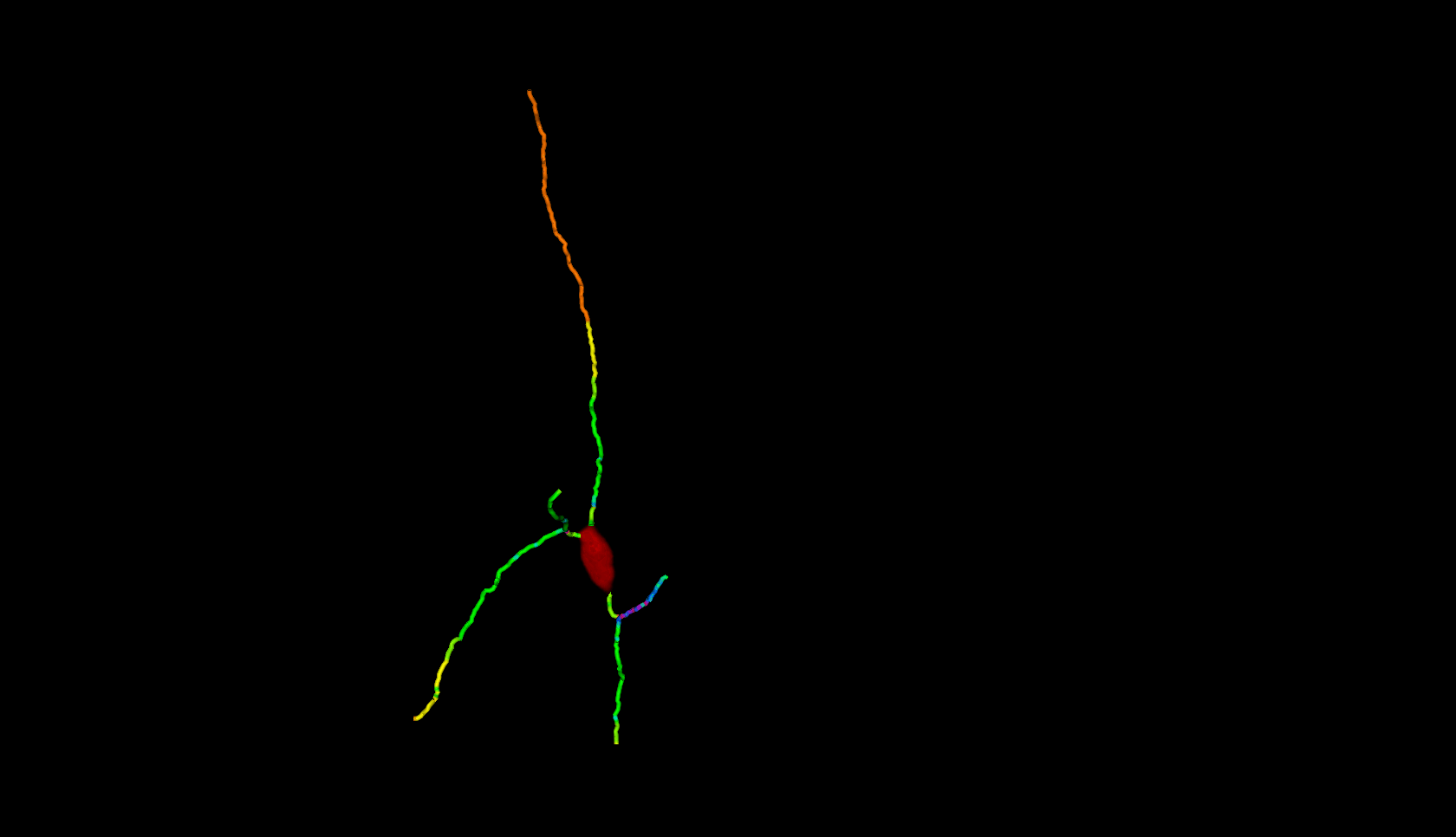

Supplement: Figure 5—source data 2. — This zip archive contains morphological images of all traced neurons grouped according their electrophysiological profiles. Each subtype’s folder contains a PDF file (with the list of neurons, their original location within the VTA, images of the traced morphology and individual Sholl curves) and two subfolders: ‘3D_gif’ – with *.gif files of the listed neurons; and ‘WaveFront_3D_obj’ – with corresponding *.obj files. *.gif files can be opened by any image viewer. *.obj files save information about the 3D model of the neurons and can be opened/reused with any 3D viewer or graphic software. [file elife-59328-fig5-data2.zip › Morphology_Source/ADP/3D_gif/4.gif]

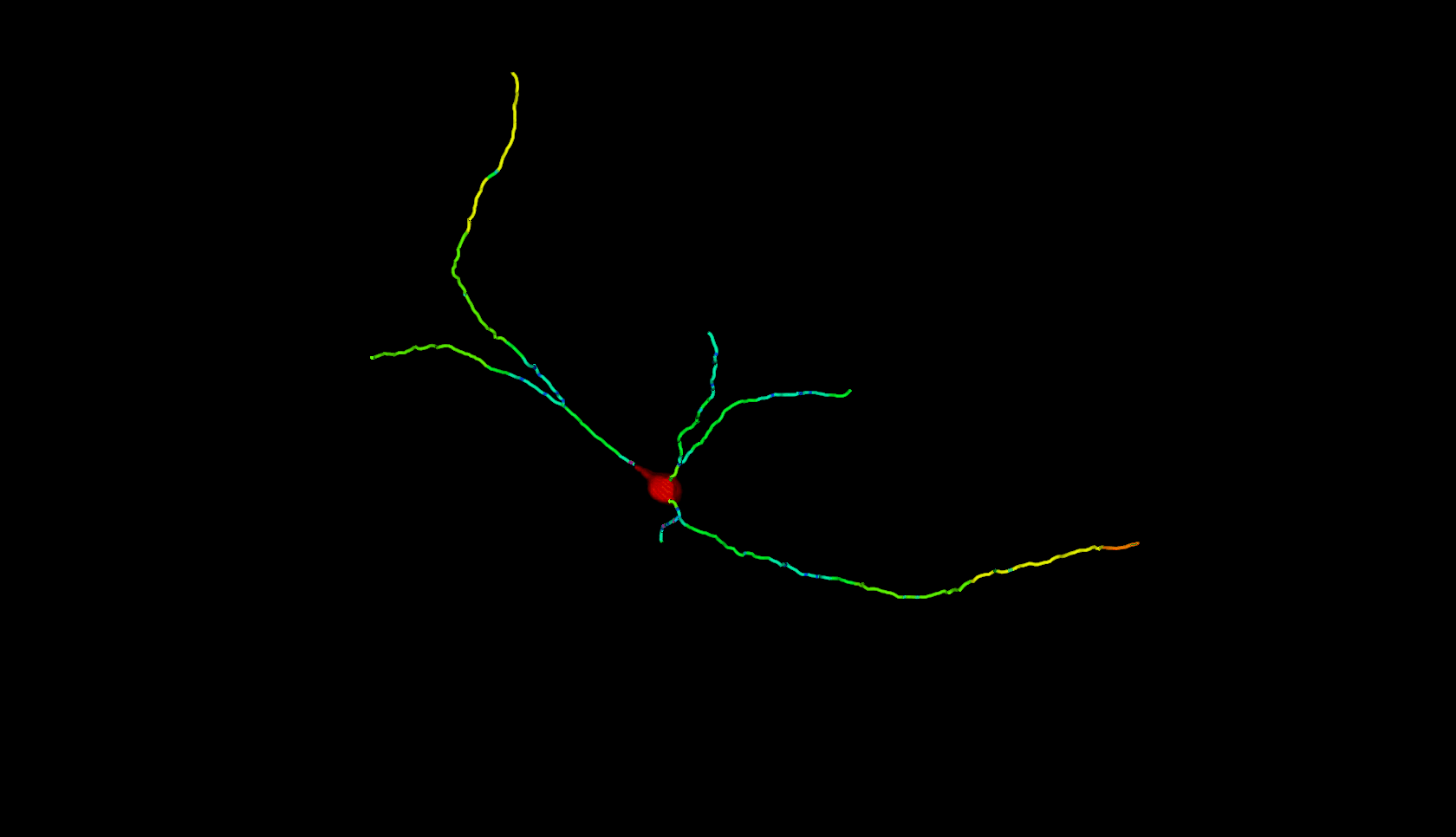

Supplement: Figure 5—source data 2. — This zip archive contains morphological images of all traced neurons grouped according their electrophysiological profiles. Each subtype’s folder contains a PDF file (with the list of neurons, their original location within the VTA, images of the traced morphology and individual Sholl curves) and two subfolders: ‘3D_gif’ – with *.gif files of the listed neurons; and ‘WaveFront_3D_obj’ – with corresponding *.obj files. *.gif files can be opened by any image viewer. *.obj files save information about the 3D model of the neurons and can be opened/reused with any 3D viewer or graphic software. [file elife-59328-fig5-data2.zip › Morphology_Source/ADP/3D_gif/5.gif]

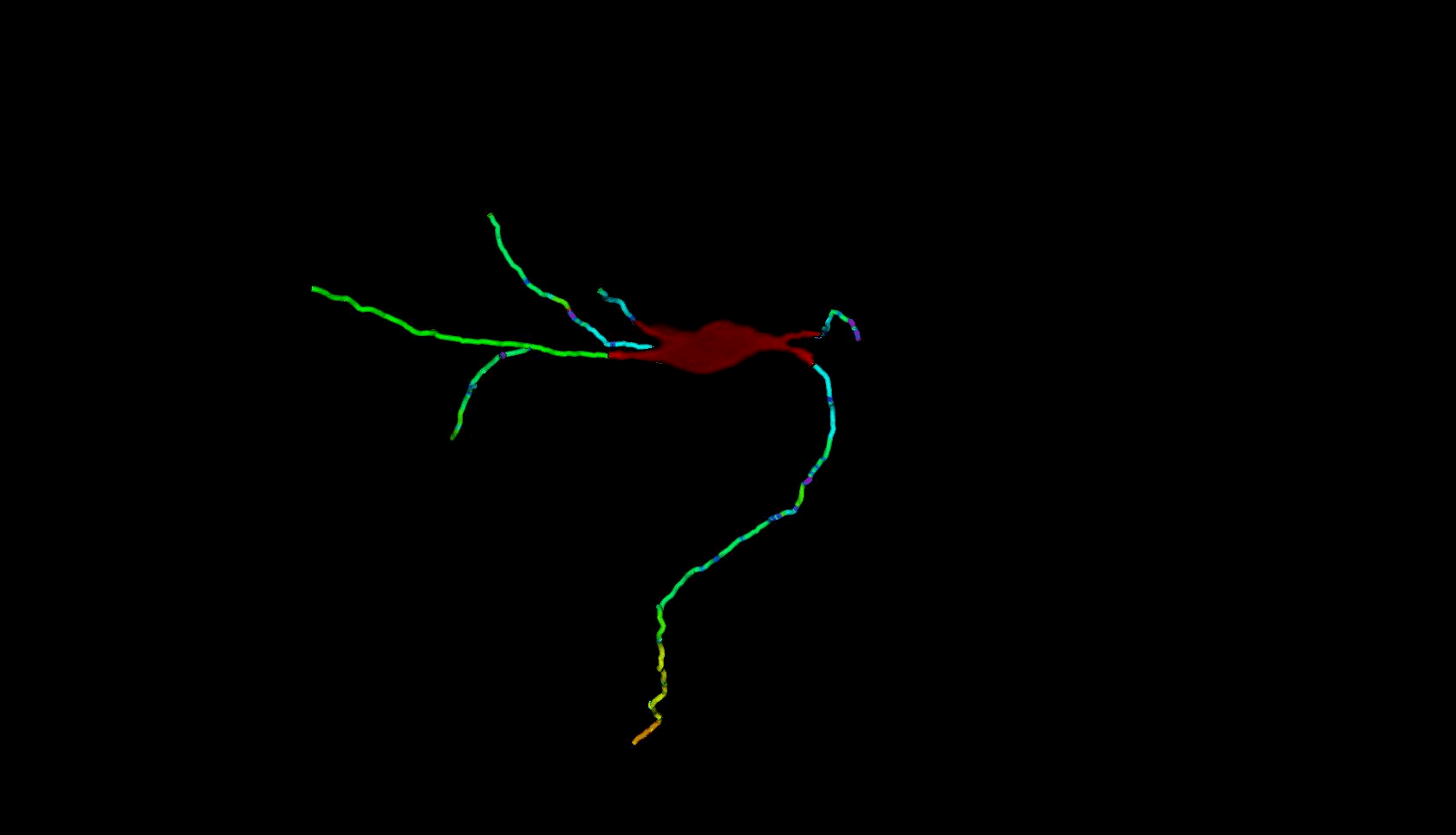

Supplement: Figure 5—source data 2. — This zip archive contains morphological images of all traced neurons grouped according their electrophysiological profiles. Each subtype’s folder contains a PDF file (with the list of neurons, their original location within the VTA, images of the traced morphology and individual Sholl curves) and two subfolders: ‘3D_gif’ – with *.gif files of the listed neurons; and ‘WaveFront_3D_obj’ – with corresponding *.obj files. *.gif files can be opened by any image viewer. *.obj files save information about the 3D model of the neurons and can be opened/reused with any 3D viewer or graphic software. [file elife-59328-fig5-data2.zip › Morphology_Source/ADP/3D_gif/6.gif]

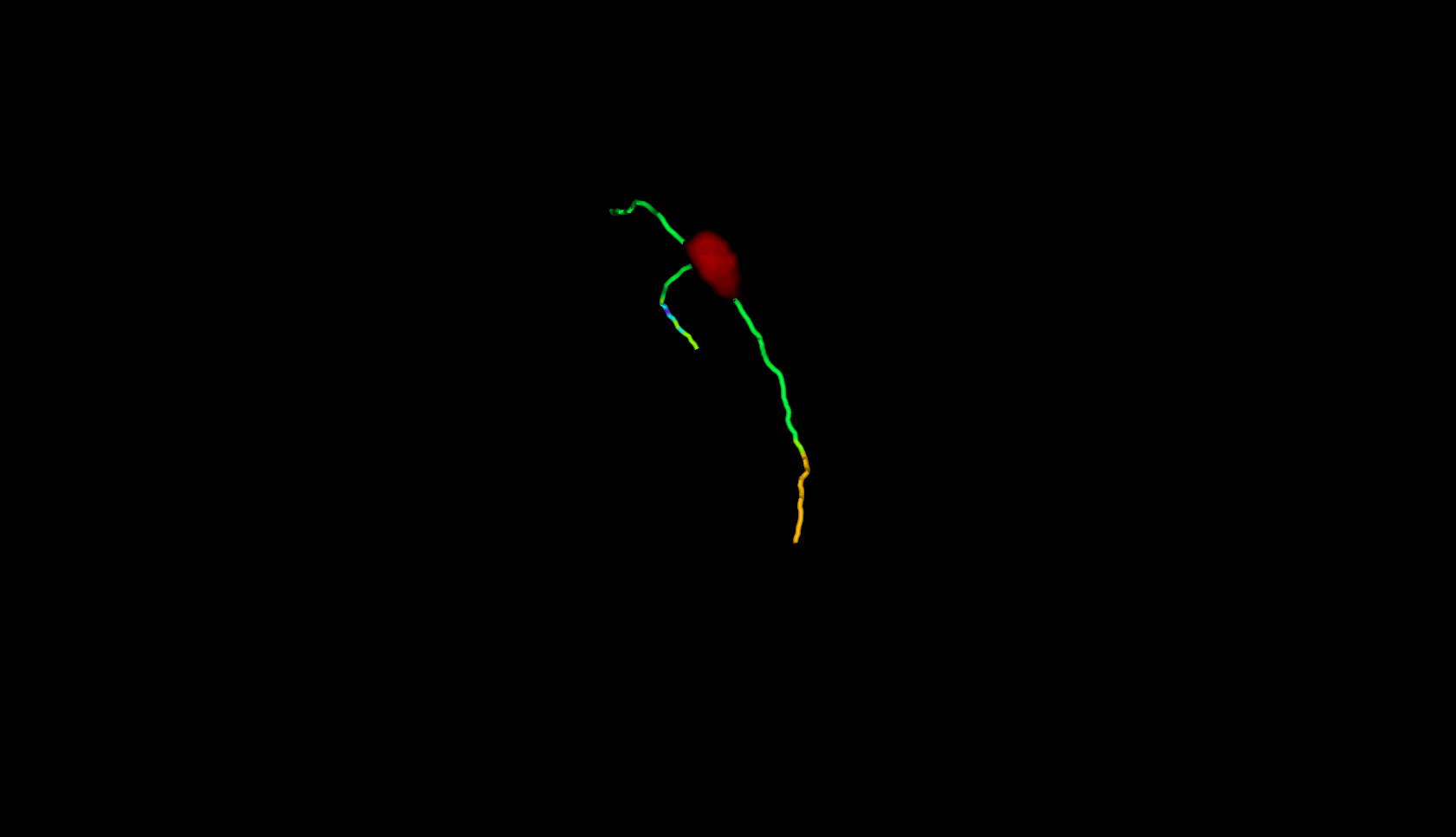

Supplement: Figure 5—source data 2. — This zip archive contains morphological images of all traced neurons grouped according their electrophysiological profiles. Each subtype’s folder contains a PDF file (with the list of neurons, their original location within the VTA, images of the traced morphology and individual Sholl curves) and two subfolders: ‘3D_gif’ – with *.gif files of the listed neurons; and ‘WaveFront_3D_obj’ – with corresponding *.obj files. *.gif files can be opened by any image viewer. *.obj files save information about the 3D model of the neurons and can be opened/reused with any 3D viewer or graphic software. [file elife-59328-fig5-data2.zip › Morphology_Source/ADP/3D_gif/7.gif]

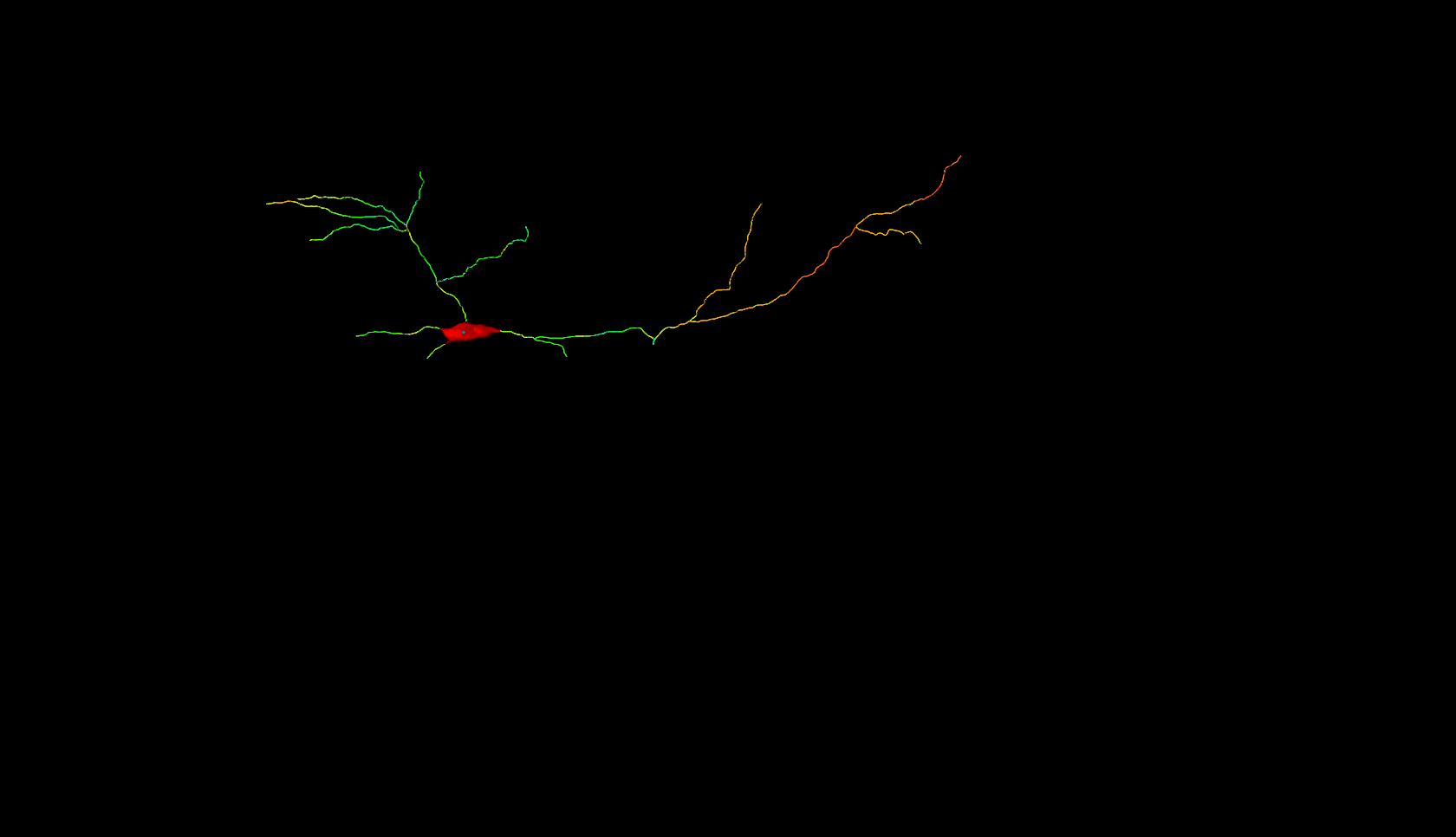

Supplement: Figure 5—source data 2. — This zip archive contains morphological images of all traced neurons grouped according their electrophysiological profiles. Each subtype’s folder contains a PDF file (with the list of neurons, their original location within the VTA, images of the traced morphology and individual Sholl curves) and two subfolders: ‘3D_gif’ – with *.gif files of the listed neurons; and ‘WaveFront_3D_obj’ – with corresponding *.obj files. *.gif files can be opened by any image viewer. *.obj files save information about the 3D model of the neurons and can be opened/reused with any 3D viewer or graphic software. [file elife-59328-fig5-data2.zip › Morphology_Source/ADP/3D_gif/8.gif]

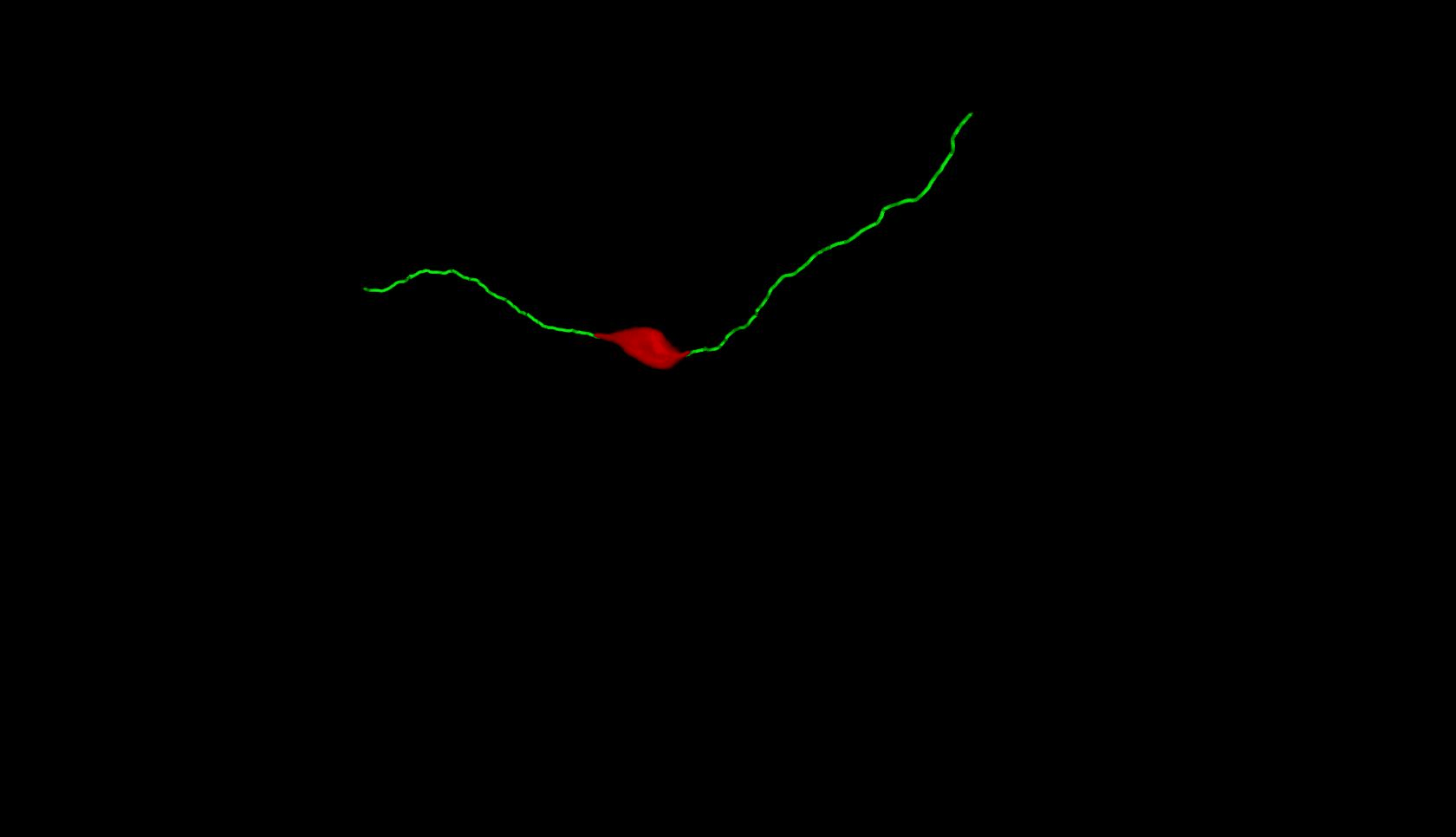

Supplement: Figure 5—source data 2. — This zip archive contains morphological images of all traced neurons grouped according their electrophysiological profiles. Each subtype’s folder contains a PDF file (with the list of neurons, their original location within the VTA, images of the traced morphology and individual Sholl curves) and two subfolders: ‘3D_gif’ – with *.gif files of the listed neurons; and ‘WaveFront_3D_obj’ – with corresponding *.obj files. *.gif files can be opened by any image viewer. *.obj files save information about the 3D model of the neurons and can be opened/reused with any 3D viewer or graphic software. [file elife-59328-fig5-data2.zip › Morphology_Source/ADP/3D_gif/9.gif]

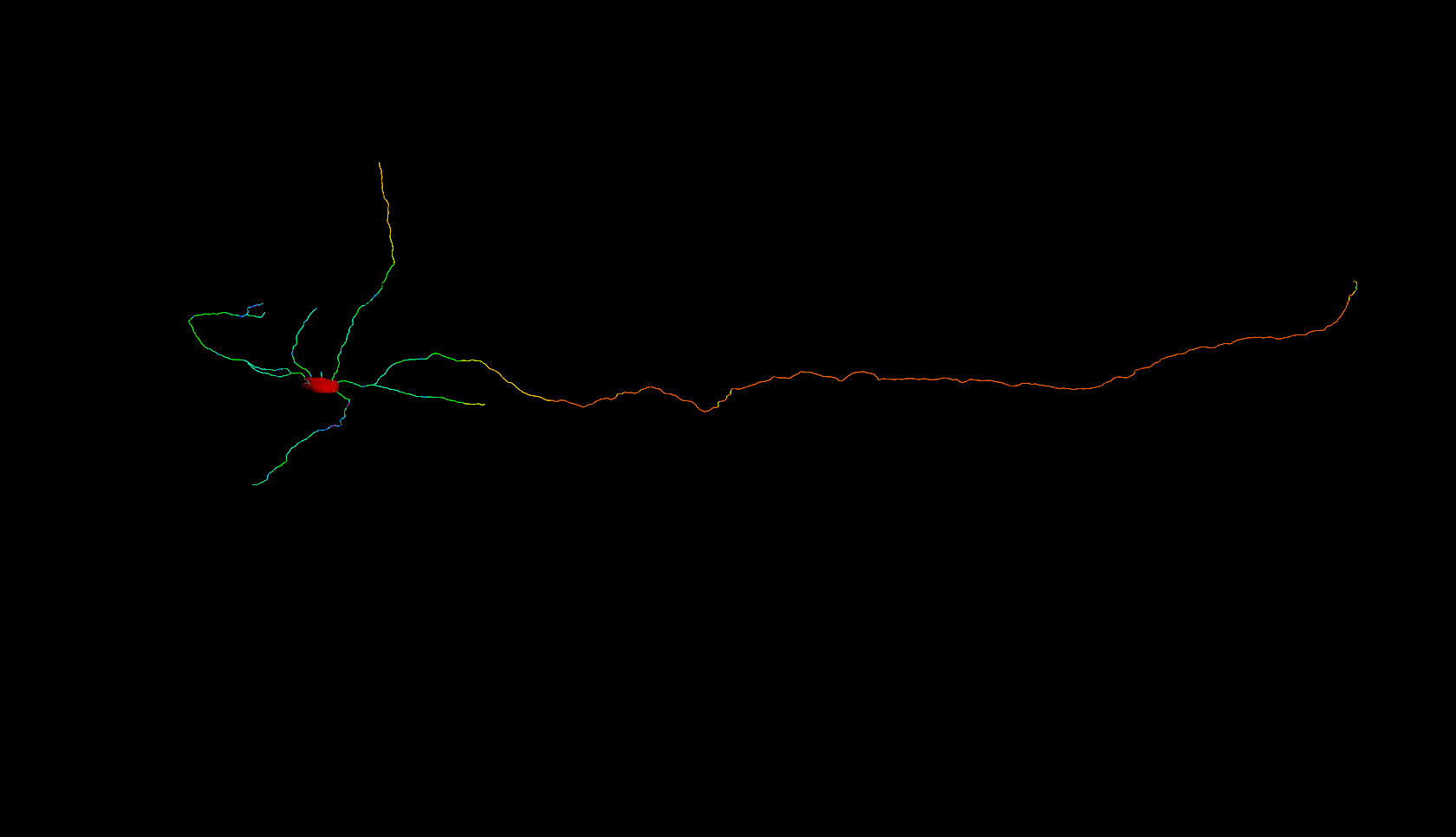

Supplement: Figure 5—source data 2. — This zip archive contains morphological images of all traced neurons grouped according their electrophysiological profiles. Each subtype’s folder contains a PDF file (with the list of neurons, their original location within the VTA, images of the traced morphology and individual Sholl curves) and two subfolders: ‘3D_gif’ – with *.gif files of the listed neurons; and ‘WaveFront_3D_obj’ – with corresponding *.obj files. *.gif files can be opened by any image viewer. *.obj files save information about the 3D model of the neurons and can be opened/reused with any 3D viewer or graphic software. [file elife-59328-fig5-data2.zip › Morphology_Source/Delayed/3D_gif/1.gif]

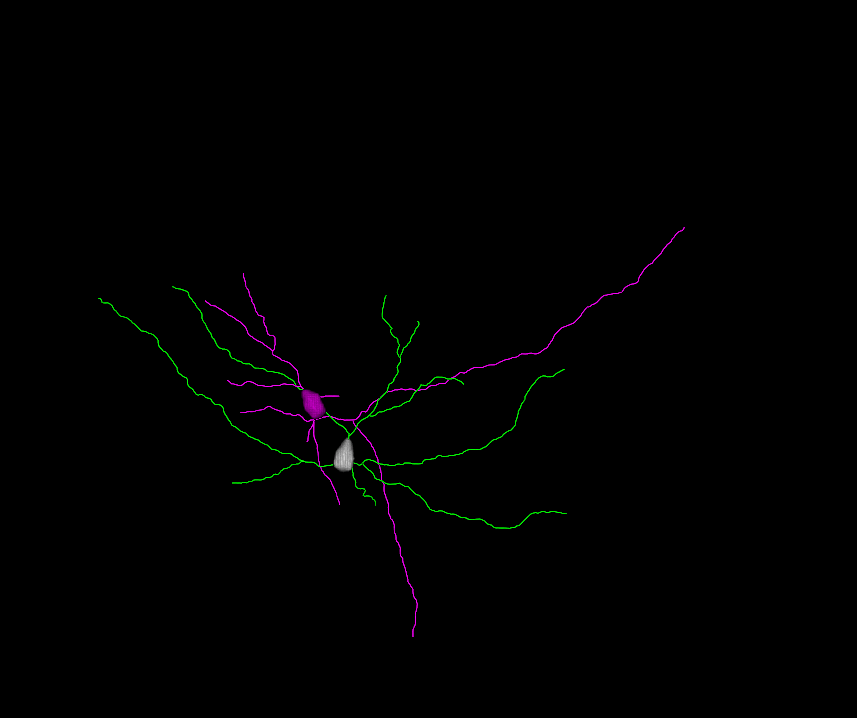

Supplement: Figure 5—source data 2. — This zip archive contains morphological images of all traced neurons grouped according their electrophysiological profiles. Each subtype’s folder contains a PDF file (with the list of neurons, their original location within the VTA, images of the traced morphology and individual Sholl curves) and two subfolders: ‘3D_gif’ – with *.gif files of the listed neurons; and ‘WaveFront_3D_obj’ – with corresponding *.obj files. *.gif files can be opened by any image viewer. *.obj files save information about the 3D model of the neurons and can be opened/reused with any 3D viewer or graphic software. [file elife-59328-fig5-data2.zip › Morphology_Source/Delayed/3D_gif/11+12.gif]

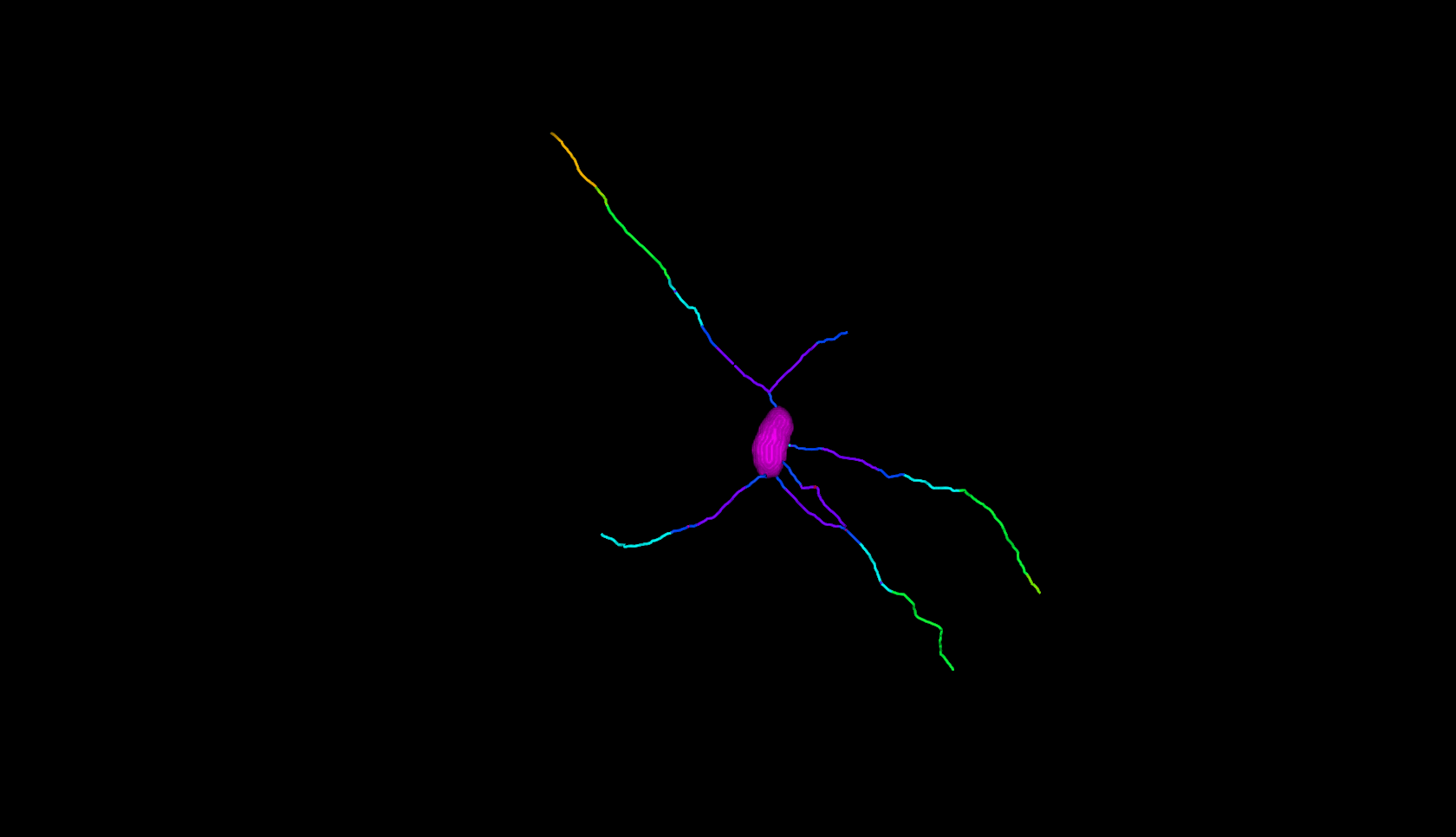

Supplement: Figure 5—source data 2. — This zip archive contains morphological images of all traced neurons grouped according their electrophysiological profiles. Each subtype’s folder contains a PDF file (with the list of neurons, their original location within the VTA, images of the traced morphology and individual Sholl curves) and two subfolders: ‘3D_gif’ – with *.gif files of the listed neurons; and ‘WaveFront_3D_obj’ – with corresponding *.obj files. *.gif files can be opened by any image viewer. *.obj files save information about the 3D model of the neurons and can be opened/reused with any 3D viewer or graphic software. [file elife-59328-fig5-data2.zip › Morphology_Source/Delayed/3D_gif/13.gif]

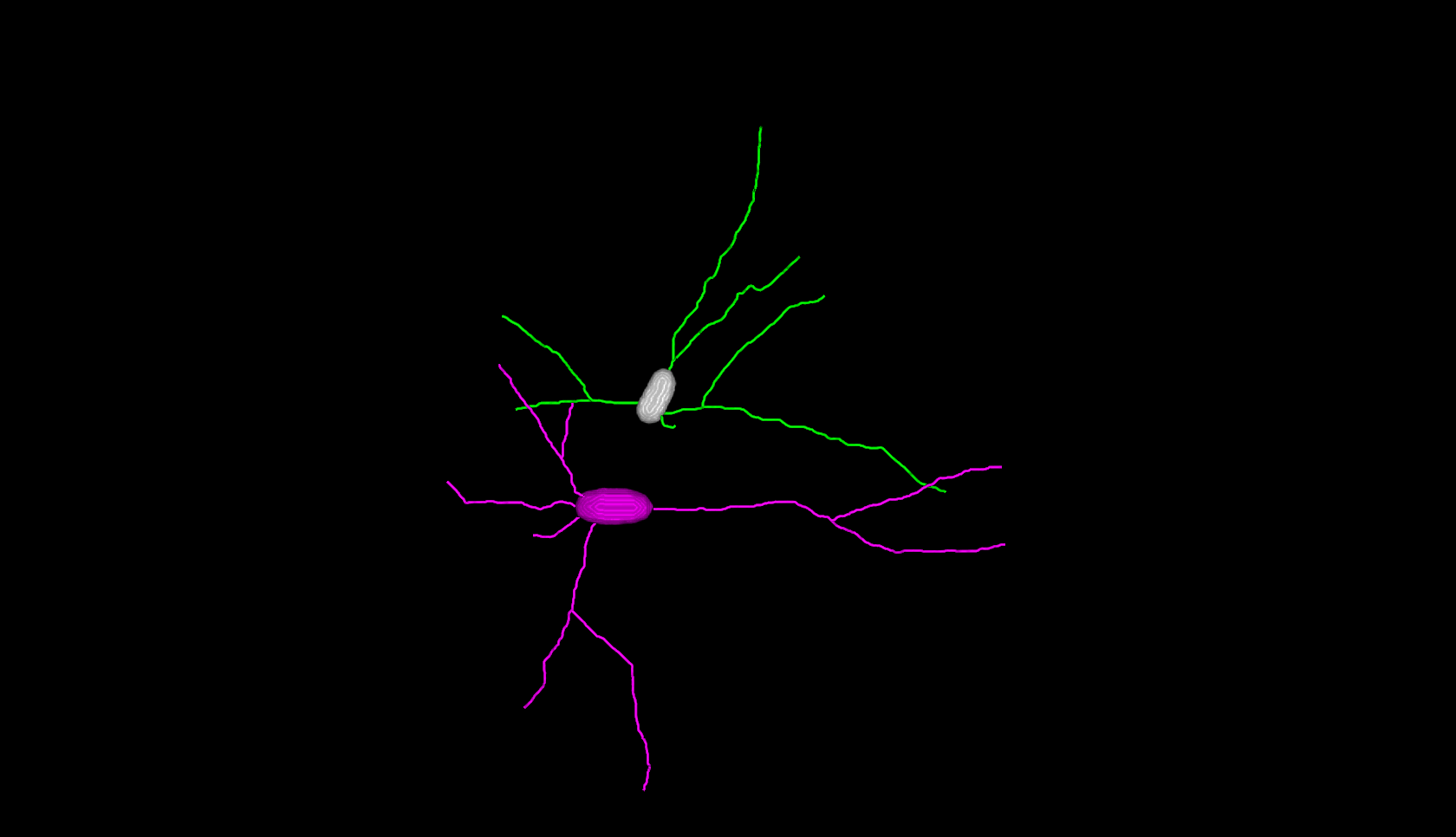

Supplement: Figure 5—source data 2. — This zip archive contains morphological images of all traced neurons grouped according their electrophysiological profiles. Each subtype’s folder contains a PDF file (with the list of neurons, their original location within the VTA, images of the traced morphology and individual Sholl curves) and two subfolders: ‘3D_gif’ – with *.gif files of the listed neurons; and ‘WaveFront_3D_obj’ – with corresponding *.obj files. *.gif files can be opened by any image viewer. *.obj files save information about the 3D model of the neurons and can be opened/reused with any 3D viewer or graphic software. [file elife-59328-fig5-data2.zip › Morphology_Source/Delayed/3D_gif/14+15.gif]

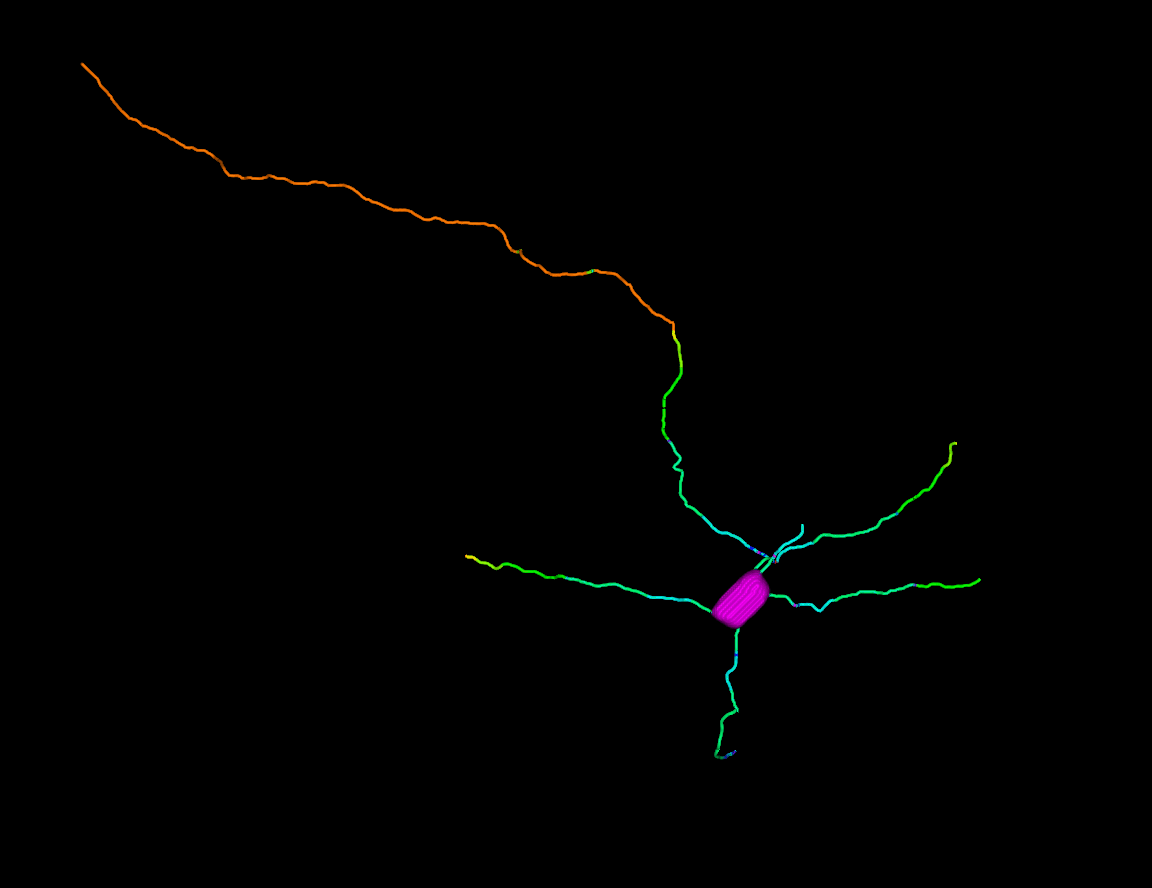

Supplement: Figure 5—source data 2. — This zip archive contains morphological images of all traced neurons grouped according their electrophysiological profiles. Each subtype’s folder contains a PDF file (with the list of neurons, their original location within the VTA, images of the traced morphology and individual Sholl curves) and two subfolders: ‘3D_gif’ – with *.gif files of the listed neurons; and ‘WaveFront_3D_obj’ – with corresponding *.obj files. *.gif files can be opened by any image viewer. *.obj files save information about the 3D model of the neurons and can be opened/reused with any 3D viewer or graphic software. [file elife-59328-fig5-data2.zip › Morphology_Source/Delayed/3D_gif/16.gif]

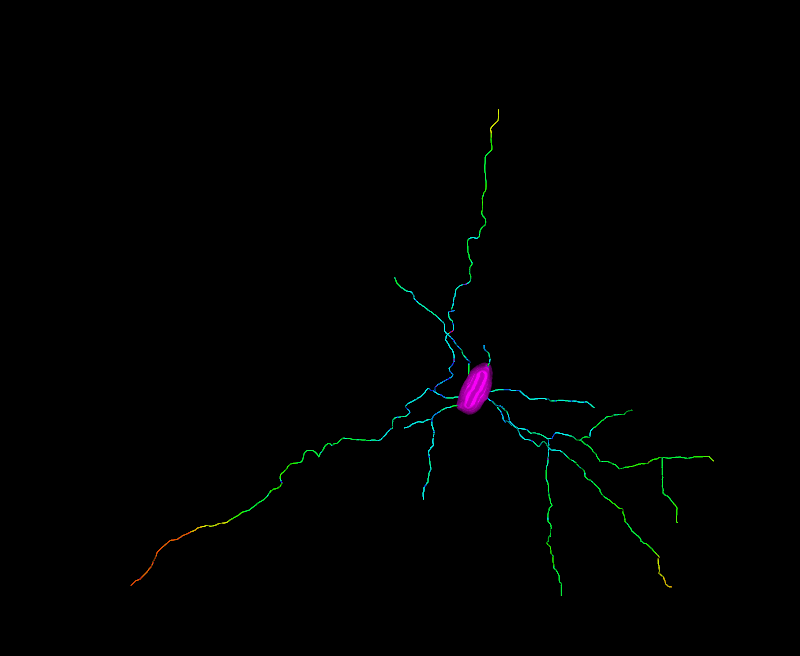

Supplement: Figure 5—source data 2. — This zip archive contains morphological images of all traced neurons grouped according their electrophysiological profiles. Each subtype’s folder contains a PDF file (with the list of neurons, their original location within the VTA, images of the traced morphology and individual Sholl curves) and two subfolders: ‘3D_gif’ – with *.gif files of the listed neurons; and ‘WaveFront_3D_obj’ – with corresponding *.obj files. *.gif files can be opened by any image viewer. *.obj files save information about the 3D model of the neurons and can be opened/reused with any 3D viewer or graphic software. [file elife-59328-fig5-data2.zip › Morphology_Source/Delayed/3D_gif/17.gif]

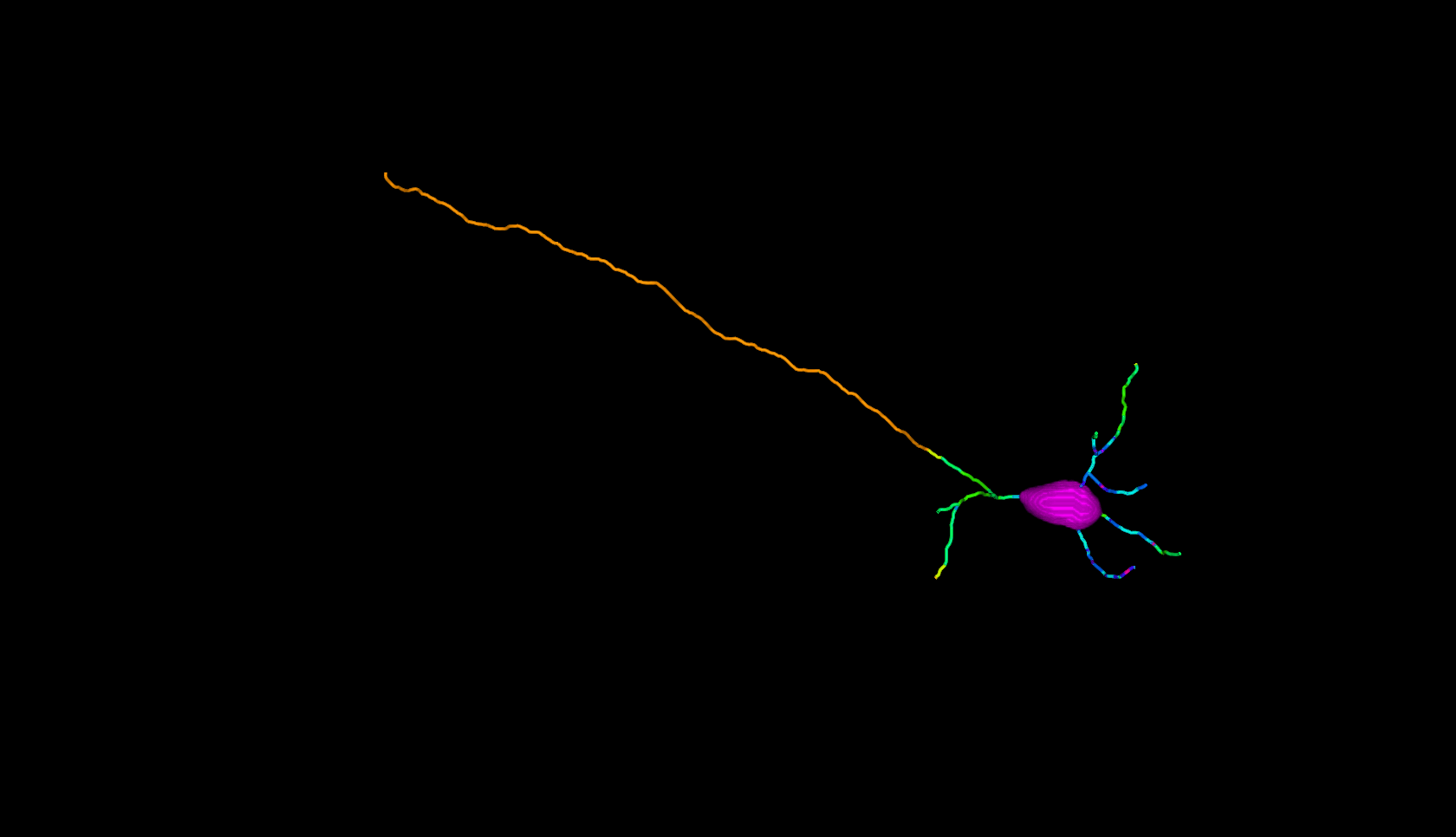

Supplement: Figure 5—source data 2. — This zip archive contains morphological images of all traced neurons grouped according their electrophysiological profiles. Each subtype’s folder contains a PDF file (with the list of neurons, their original location within the VTA, images of the traced morphology and individual Sholl curves) and two subfolders: ‘3D_gif’ – with *.gif files of the listed neurons; and ‘WaveFront_3D_obj’ – with corresponding *.obj files. *.gif files can be opened by any image viewer. *.obj files save information about the 3D model of the neurons and can be opened/reused with any 3D viewer or graphic software. [file elife-59328-fig5-data2.zip › Morphology_Source/Delayed/3D_gif/18.gif]

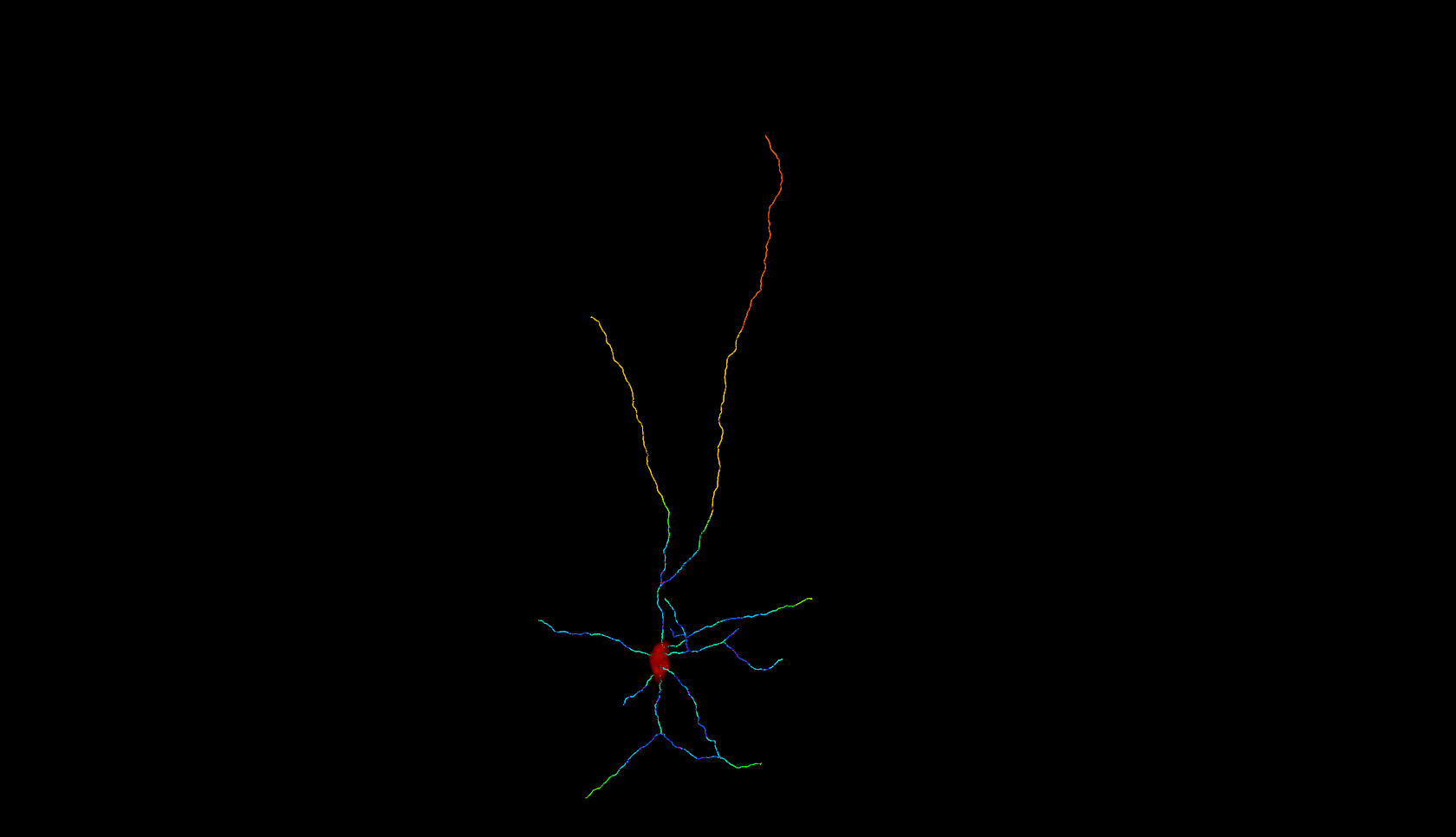

Supplement: Figure 5—source data 2. — This zip archive contains morphological images of all traced neurons grouped according their electrophysiological profiles. Each subtype’s folder contains a PDF file (with the list of neurons, their original location within the VTA, images of the traced morphology and individual Sholl curves) and two subfolders: ‘3D_gif’ – with *.gif files of the listed neurons; and ‘WaveFront_3D_obj’ – with corresponding *.obj files. *.gif files can be opened by any image viewer. *.obj files save information about the 3D model of the neurons and can be opened/reused with any 3D viewer or graphic software. [file elife-59328-fig5-data2.zip › Morphology_Source/Delayed/3D_gif/2.gif]

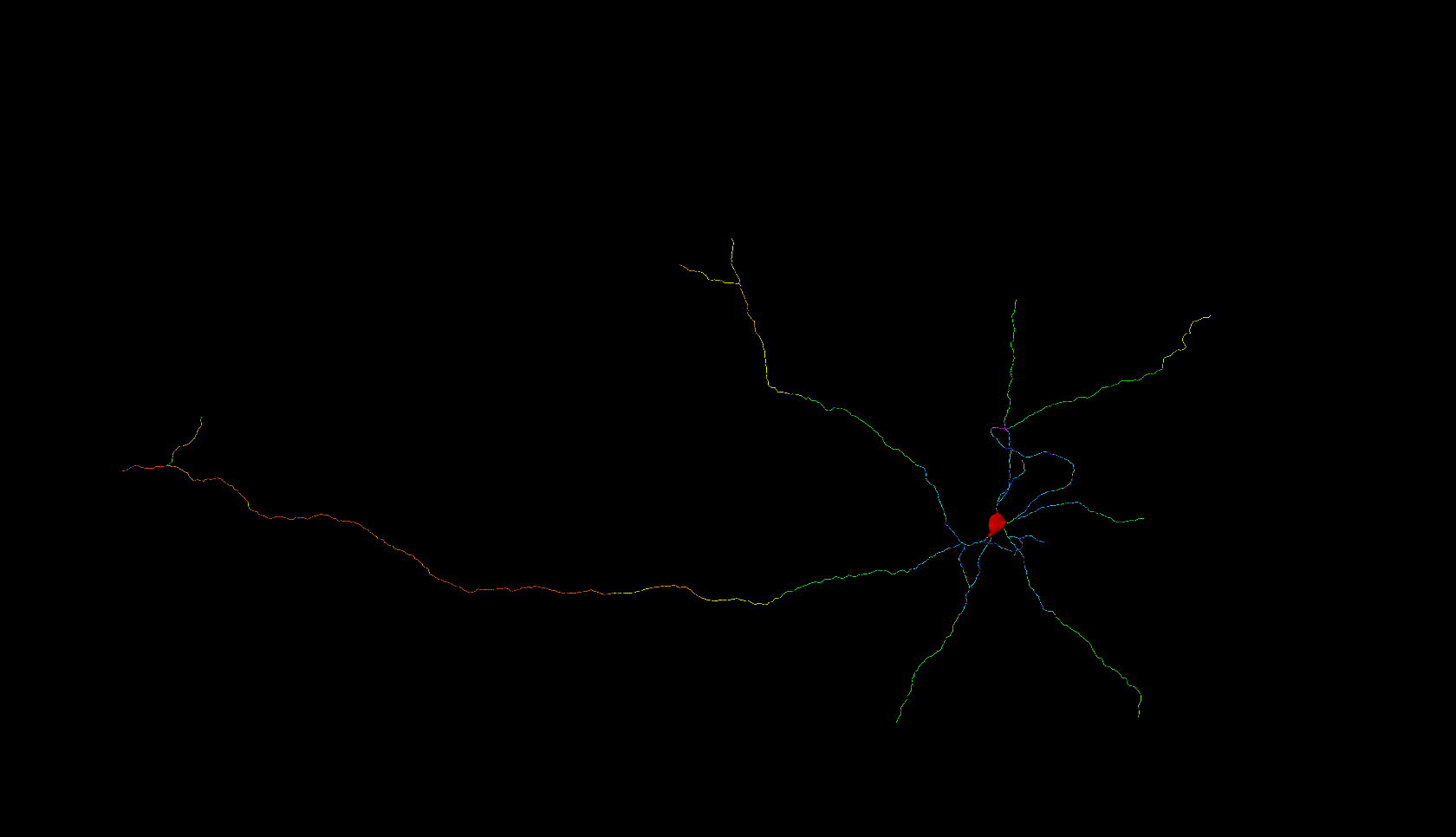

Supplement: Figure 5—source data 2. — This zip archive contains morphological images of all traced neurons grouped according their electrophysiological profiles. Each subtype’s folder contains a PDF file (with the list of neurons, their original location within the VTA, images of the traced morphology and individual Sholl curves) and two subfolders: ‘3D_gif’ – with *.gif files of the listed neurons; and ‘WaveFront_3D_obj’ – with corresponding *.obj files. *.gif files can be opened by any image viewer. *.obj files save information about the 3D model of the neurons and can be opened/reused with any 3D viewer or graphic software. [file elife-59328-fig5-data2.zip › Morphology_Source/Delayed/3D_gif/3.gif]

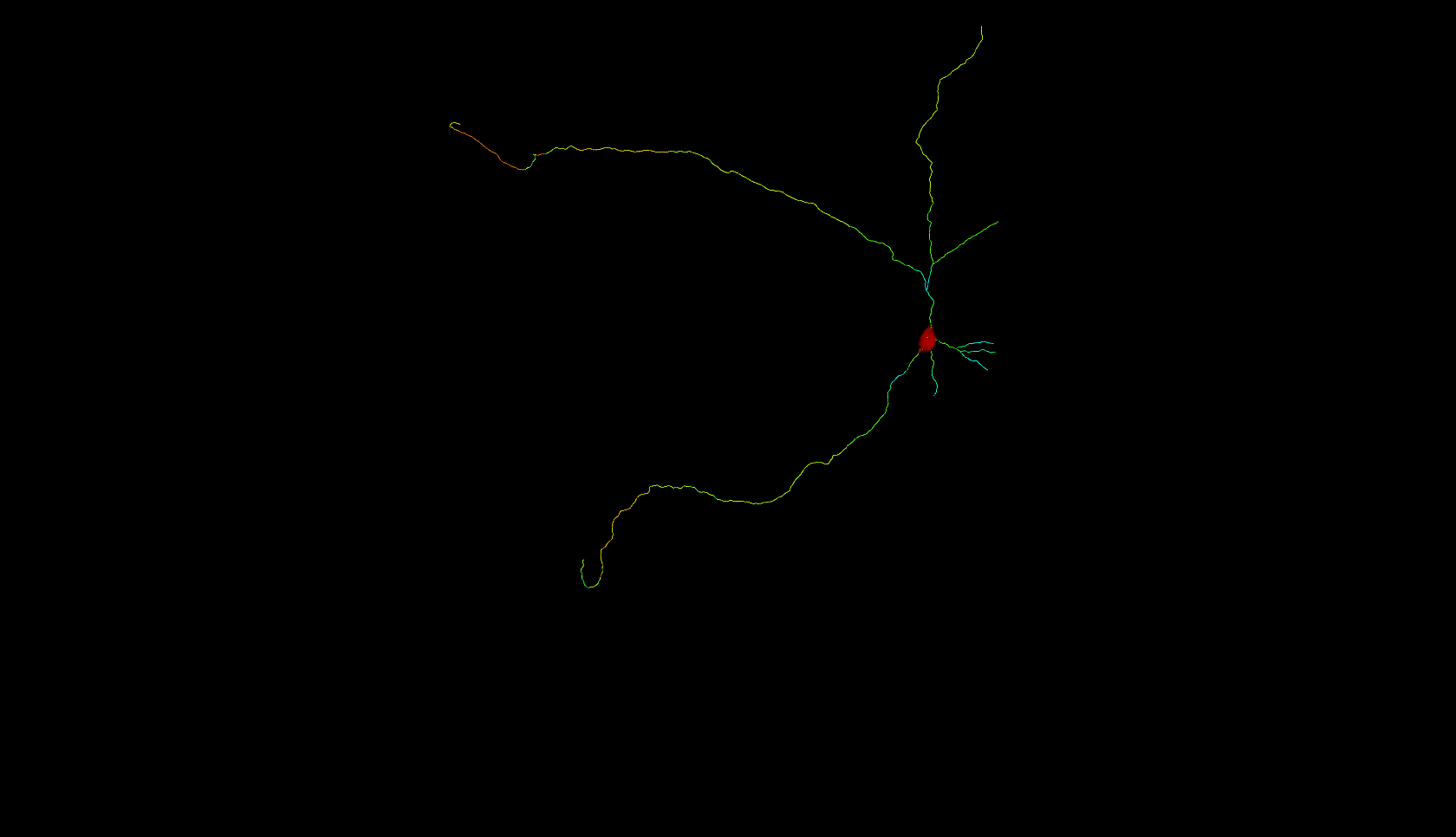

Supplement: Figure 5—source data 2. — This zip archive contains morphological images of all traced neurons grouped according their electrophysiological profiles. Each subtype’s folder contains a PDF file (with the list of neurons, their original location within the VTA, images of the traced morphology and individual Sholl curves) and two subfolders: ‘3D_gif’ – with *.gif files of the listed neurons; and ‘WaveFront_3D_obj’ – with corresponding *.obj files. *.gif files can be opened by any image viewer. *.obj files save information about the 3D model of the neurons and can be opened/reused with any 3D viewer or graphic software. [file elife-59328-fig5-data2.zip › Morphology_Source/Delayed/3D_gif/4.gif]

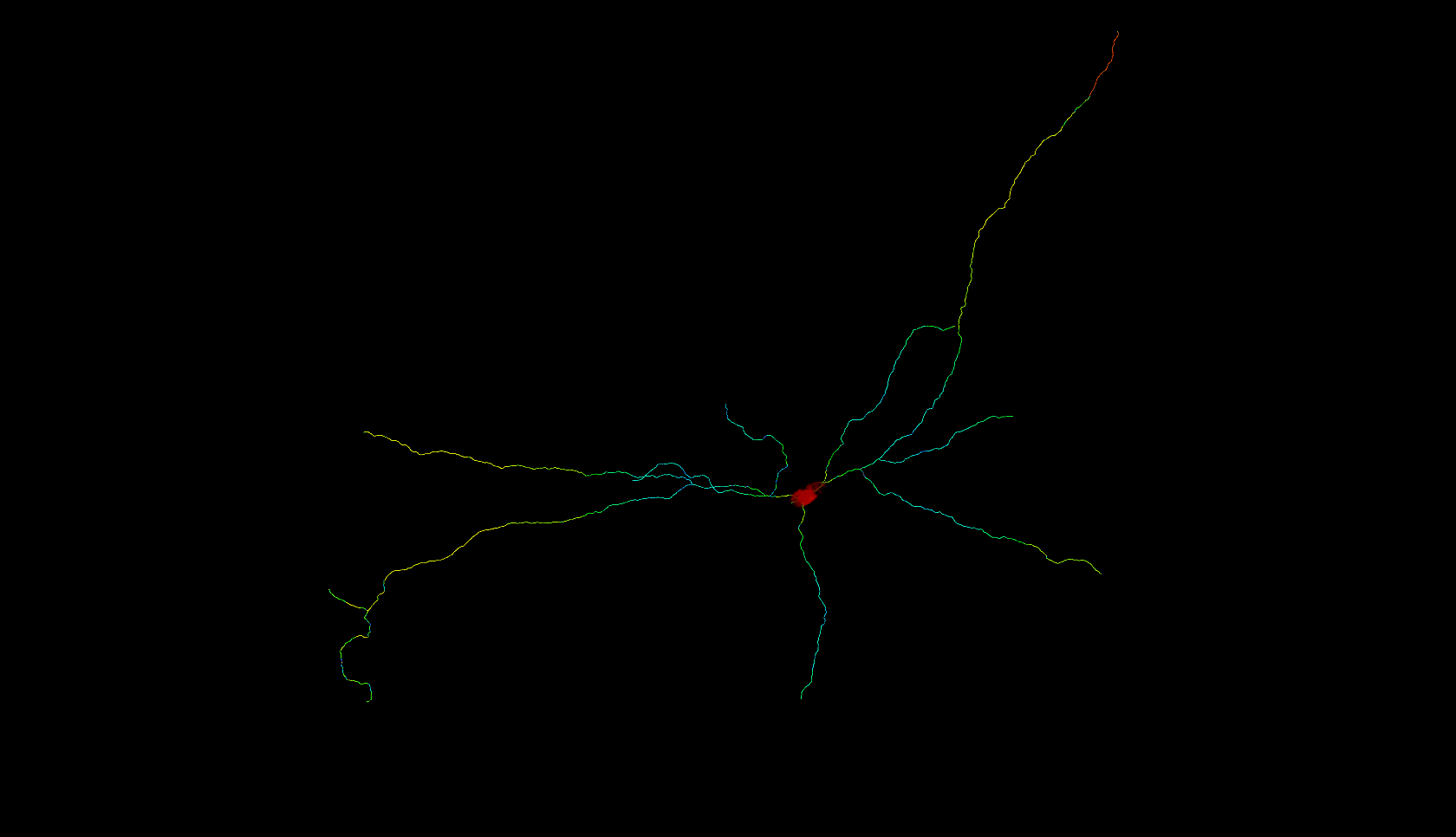

Supplement: Figure 5—source data 2. — This zip archive contains morphological images of all traced neurons grouped according their electrophysiological profiles. Each subtype’s folder contains a PDF file (with the list of neurons, their original location within the VTA, images of the traced morphology and individual Sholl curves) and two subfolders: ‘3D_gif’ – with *.gif files of the listed neurons; and ‘WaveFront_3D_obj’ – with corresponding *.obj files. *.gif files can be opened by any image viewer. *.obj files save information about the 3D model of the neurons and can be opened/reused with any 3D viewer or graphic software. [file elife-59328-fig5-data2.zip › Morphology_Source/Delayed/3D_gif/5.gif]

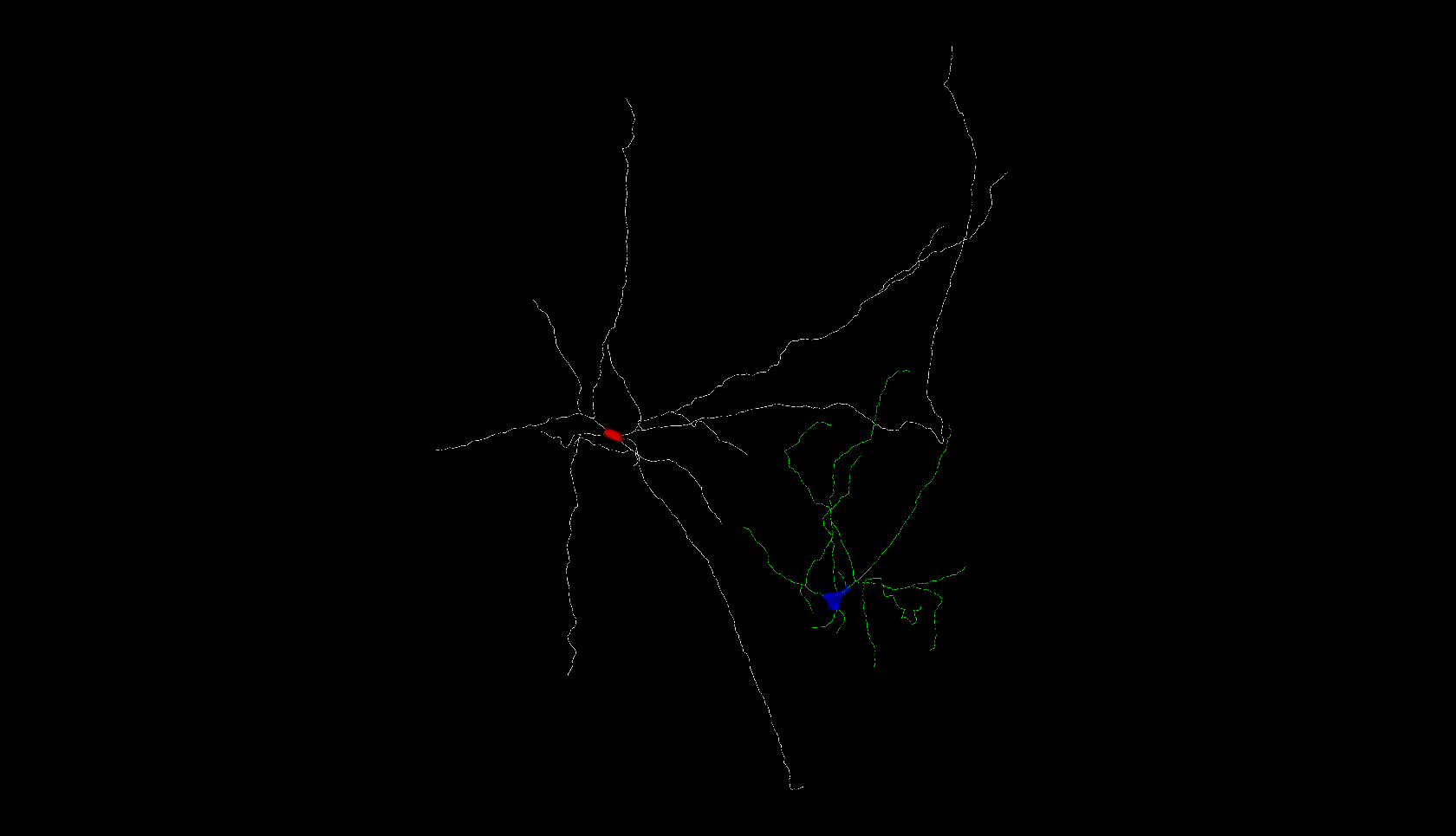

Supplement: Figure 5—source data 2. — This zip archive contains morphological images of all traced neurons grouped according their electrophysiological profiles. Each subtype’s folder contains a PDF file (with the list of neurons, their original location within the VTA, images of the traced morphology and individual Sholl curves) and two subfolders: ‘3D_gif’ – with *.gif files of the listed neurons; and ‘WaveFront_3D_obj’ – with corresponding *.obj files. *.gif files can be opened by any image viewer. *.obj files save information about the 3D model of the neurons and can be opened/reused with any 3D viewer or graphic software. [file elife-59328-fig5-data2.zip › Morphology_Source/Delayed/3D_gif/6+7.gif]

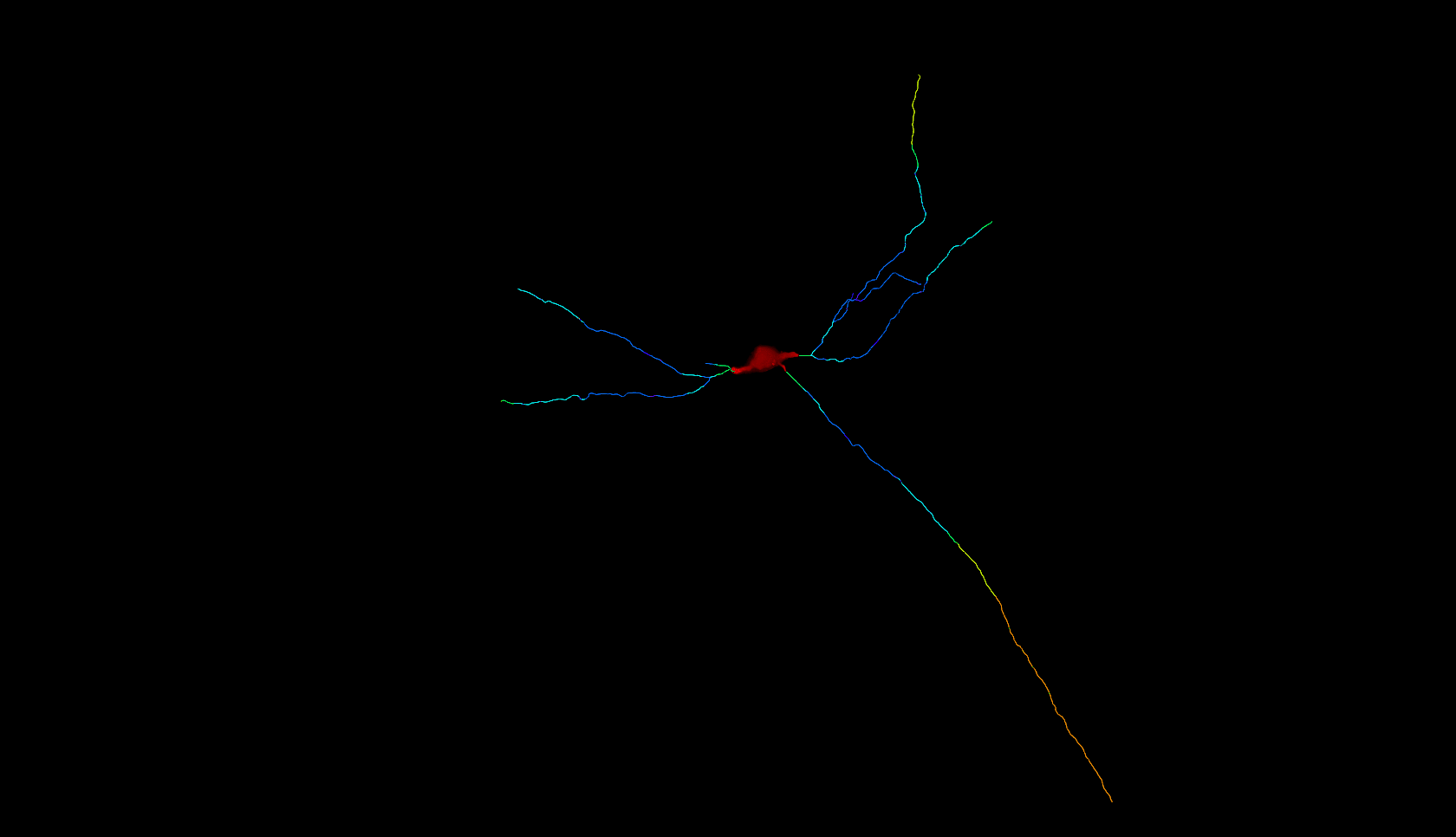

Supplement: Figure 5—source data 2. — This zip archive contains morphological images of all traced neurons grouped according their electrophysiological profiles. Each subtype’s folder contains a PDF file (with the list of neurons, their original location within the VTA, images of the traced morphology and individual Sholl curves) and two subfolders: ‘3D_gif’ – with *.gif files of the listed neurons; and ‘WaveFront_3D_obj’ – with corresponding *.obj files. *.gif files can be opened by any image viewer. *.obj files save information about the 3D model of the neurons and can be opened/reused with any 3D viewer or graphic software. [file elife-59328-fig5-data2.zip › Morphology_Source/Delayed/3D_gif/8.gif]

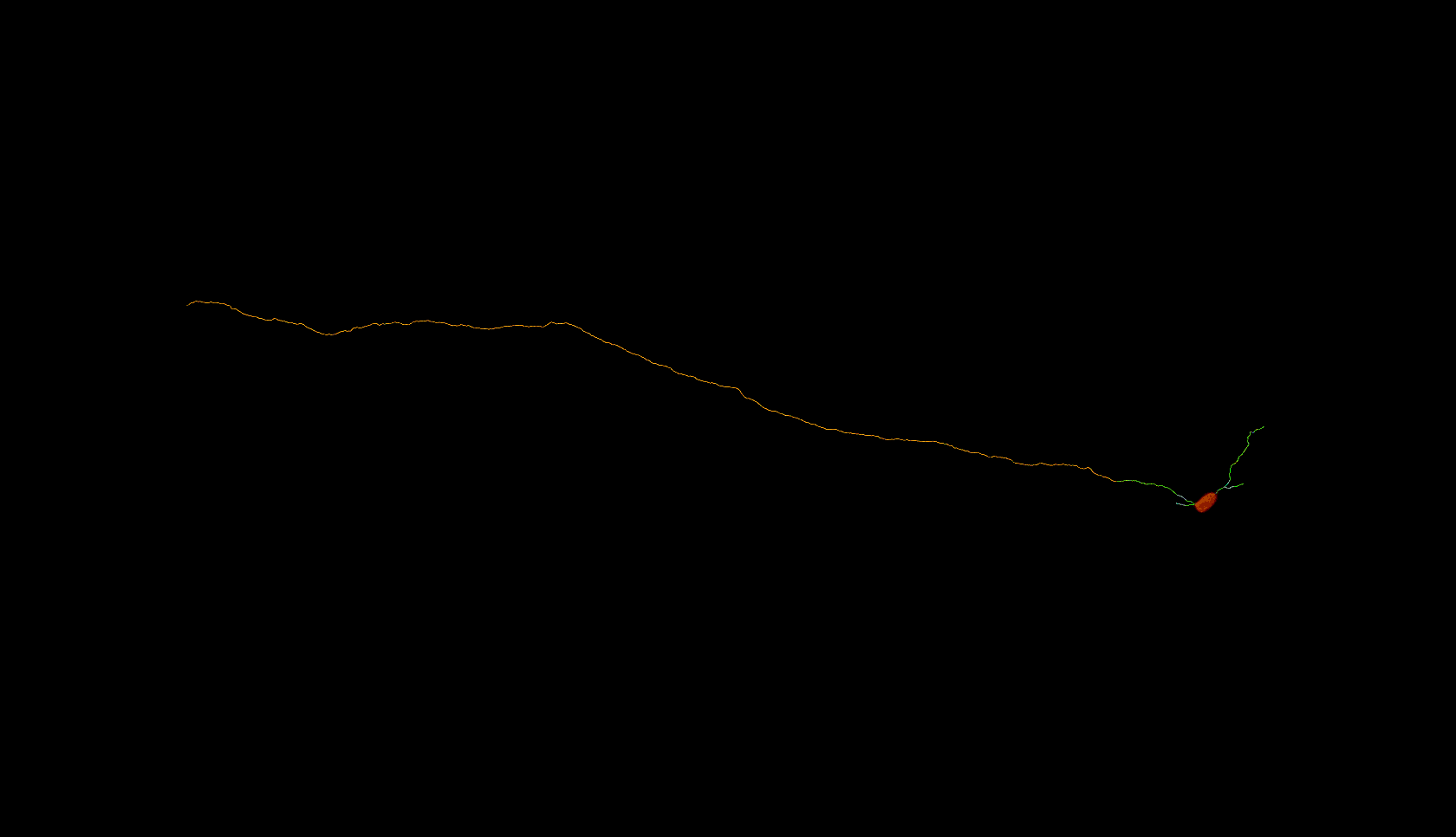

Supplement: Figure 5—source data 2. — This zip archive contains morphological images of all traced neurons grouped according their electrophysiological profiles. Each subtype’s folder contains a PDF file (with the list of neurons, their original location within the VTA, images of the traced morphology and individual Sholl curves) and two subfolders: ‘3D_gif’ – with *.gif files of the listed neurons; and ‘WaveFront_3D_obj’ – with corresponding *.obj files. *.gif files can be opened by any image viewer. *.obj files save information about the 3D model of the neurons and can be opened/reused with any 3D viewer or graphic software. [file elife-59328-fig5-data2.zip › Morphology_Source/HFF/3D_gif/1.gif]

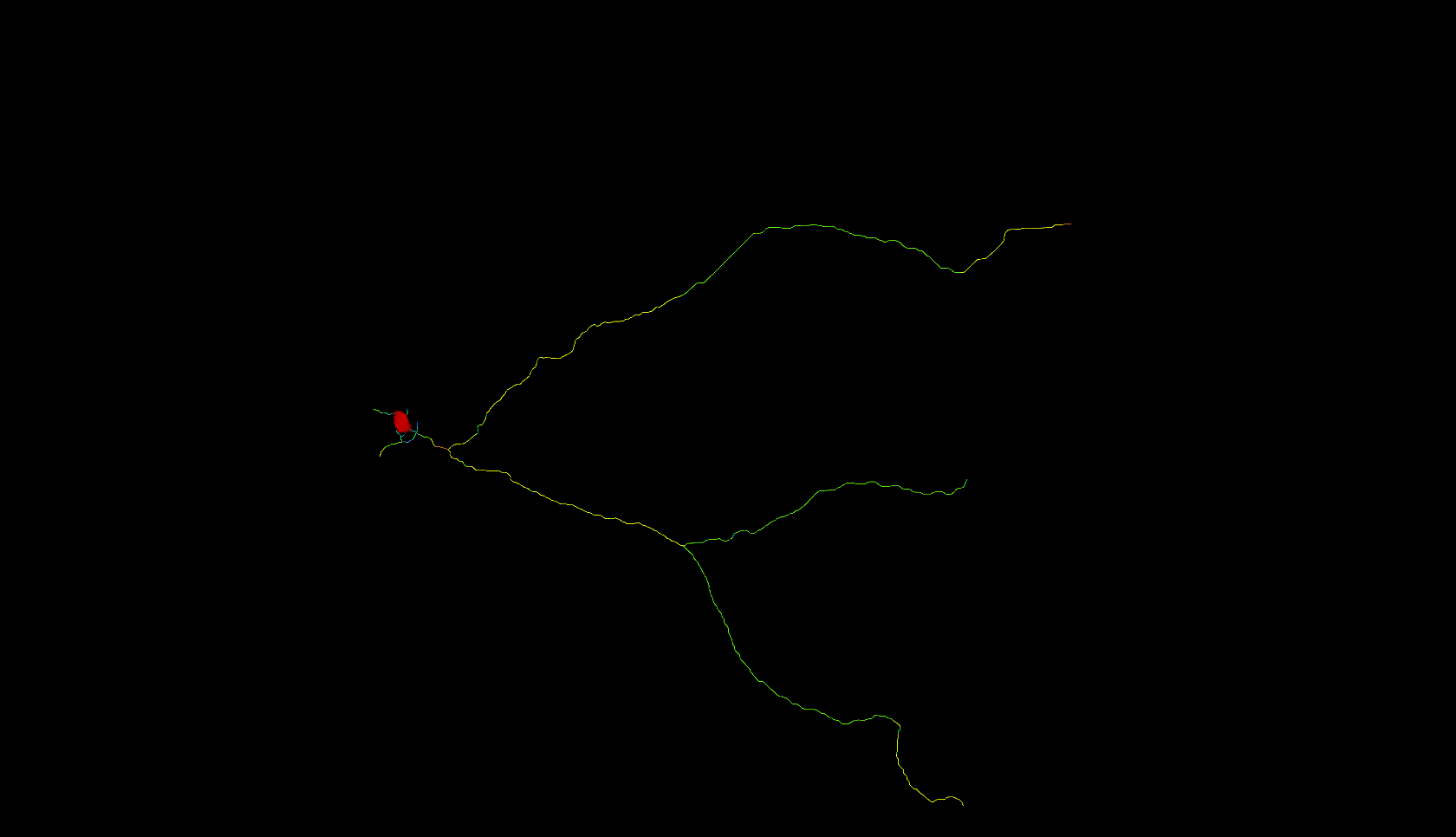

Supplement: Figure 5—source data 2. — This zip archive contains morphological images of all traced neurons grouped according their electrophysiological profiles. Each subtype’s folder contains a PDF file (with the list of neurons, their original location within the VTA, images of the traced morphology and individual Sholl curves) and two subfolders: ‘3D_gif’ – with *.gif files of the listed neurons; and ‘WaveFront_3D_obj’ – with corresponding *.obj files. *.gif files can be opened by any image viewer. *.obj files save information about the 3D model of the neurons and can be opened/reused with any 3D viewer or graphic software. [file elife-59328-fig5-data2.zip › Morphology_Source/HFF/3D_gif/2.gif]

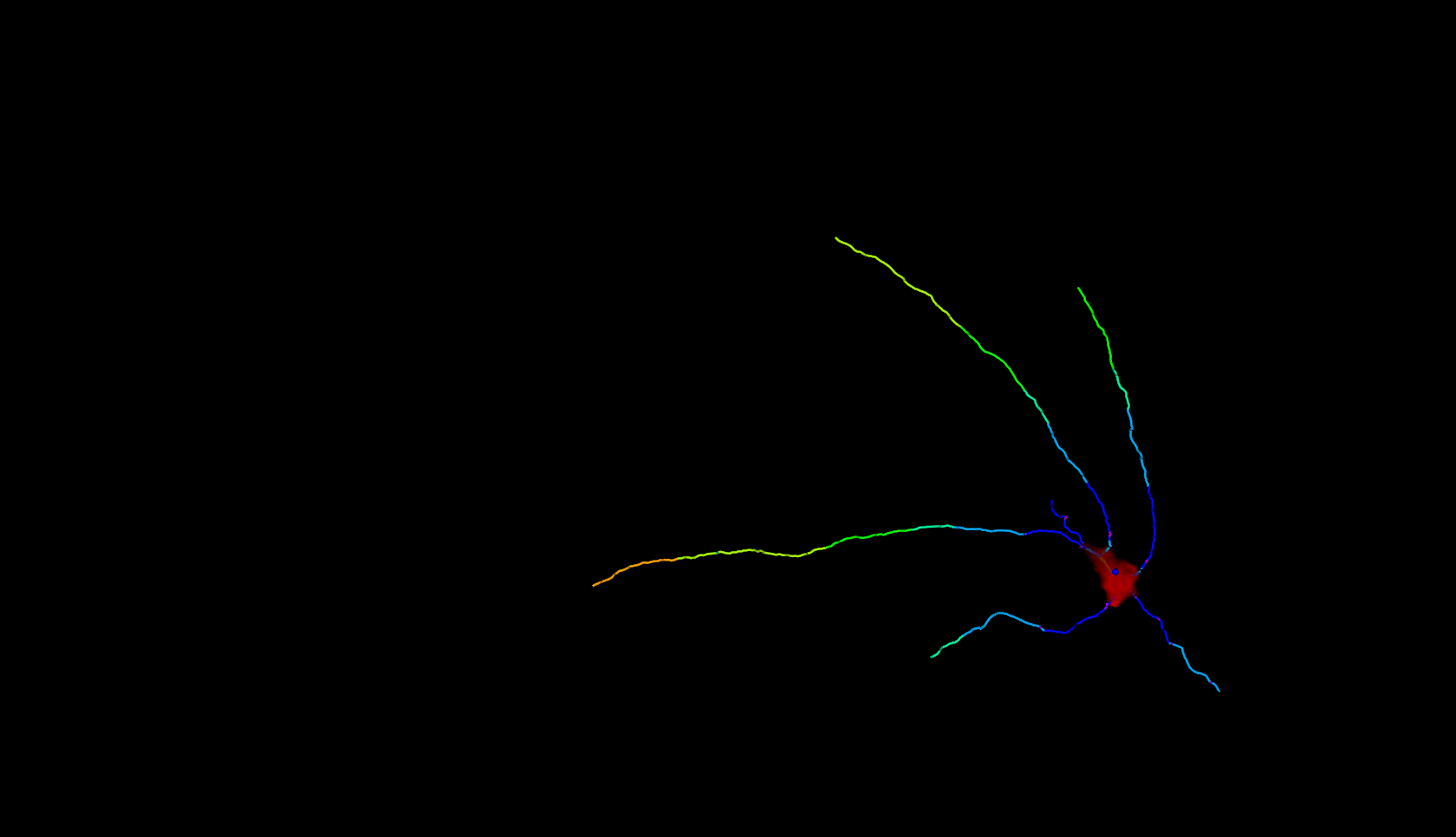

Supplement: Figure 5—source data 2. — This zip archive contains morphological images of all traced neurons grouped according their electrophysiological profiles. Each subtype’s folder contains a PDF file (with the list of neurons, their original location within the VTA, images of the traced morphology and individual Sholl curves) and two subfolders: ‘3D_gif’ – with *.gif files of the listed neurons; and ‘WaveFront_3D_obj’ – with corresponding *.obj files. *.gif files can be opened by any image viewer. *.obj files save information about the 3D model of the neurons and can be opened/reused with any 3D viewer or graphic software. [file elife-59328-fig5-data2.zip › Morphology_Source/HFF/3D_gif/3.gif]

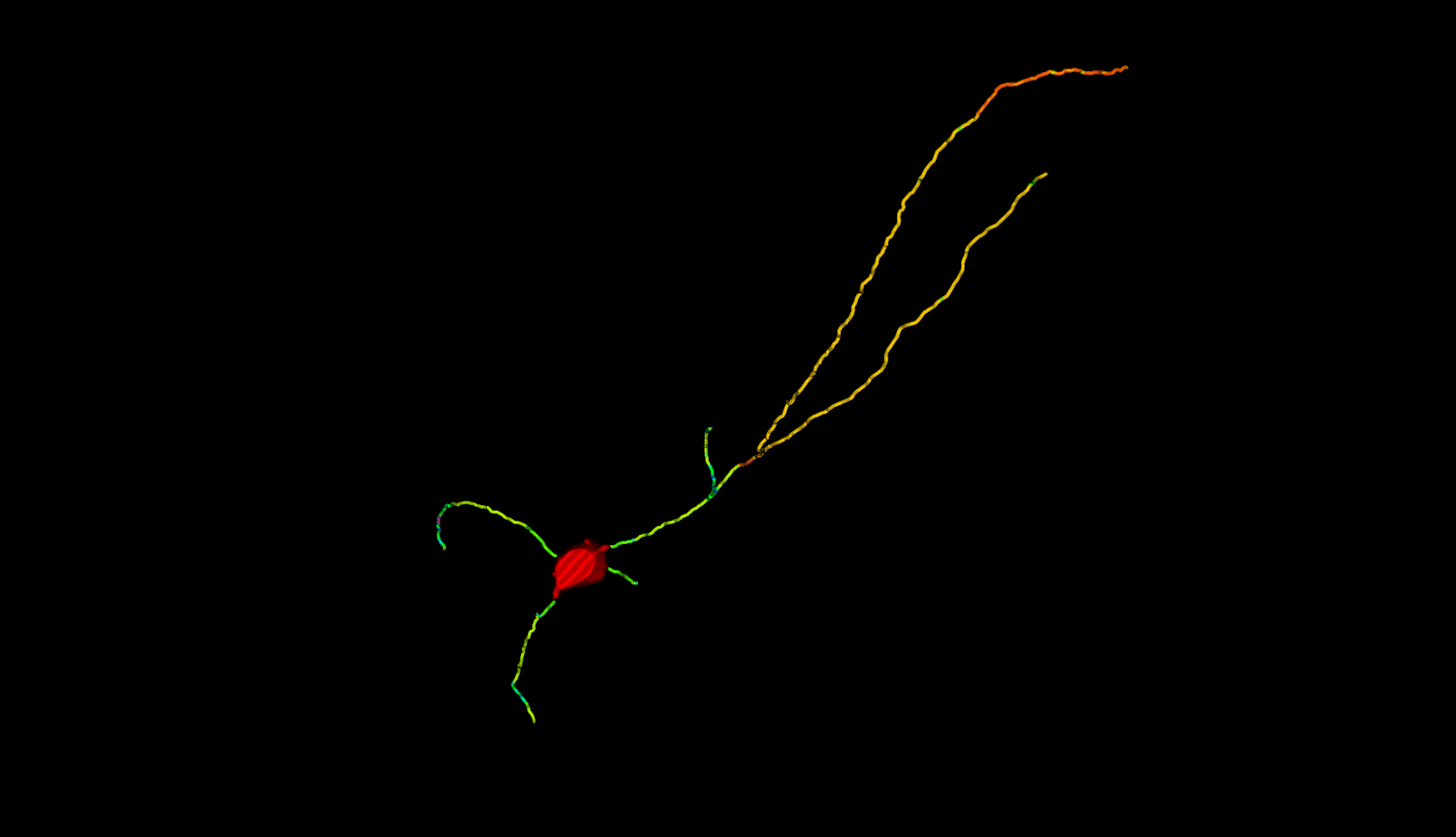

Supplement: Figure 5—source data 2. — This zip archive contains morphological images of all traced neurons grouped according their electrophysiological profiles. Each subtype’s folder contains a PDF file (with the list of neurons, their original location within the VTA, images of the traced morphology and individual Sholl curves) and two subfolders: ‘3D_gif’ – with *.gif files of the listed neurons; and ‘WaveFront_3D_obj’ – with corresponding *.obj files. *.gif files can be opened by any image viewer. *.obj files save information about the 3D model of the neurons and can be opened/reused with any 3D viewer or graphic software. [file elife-59328-fig5-data2.zip › Morphology_Source/HFF/3D_gif/5.gif]

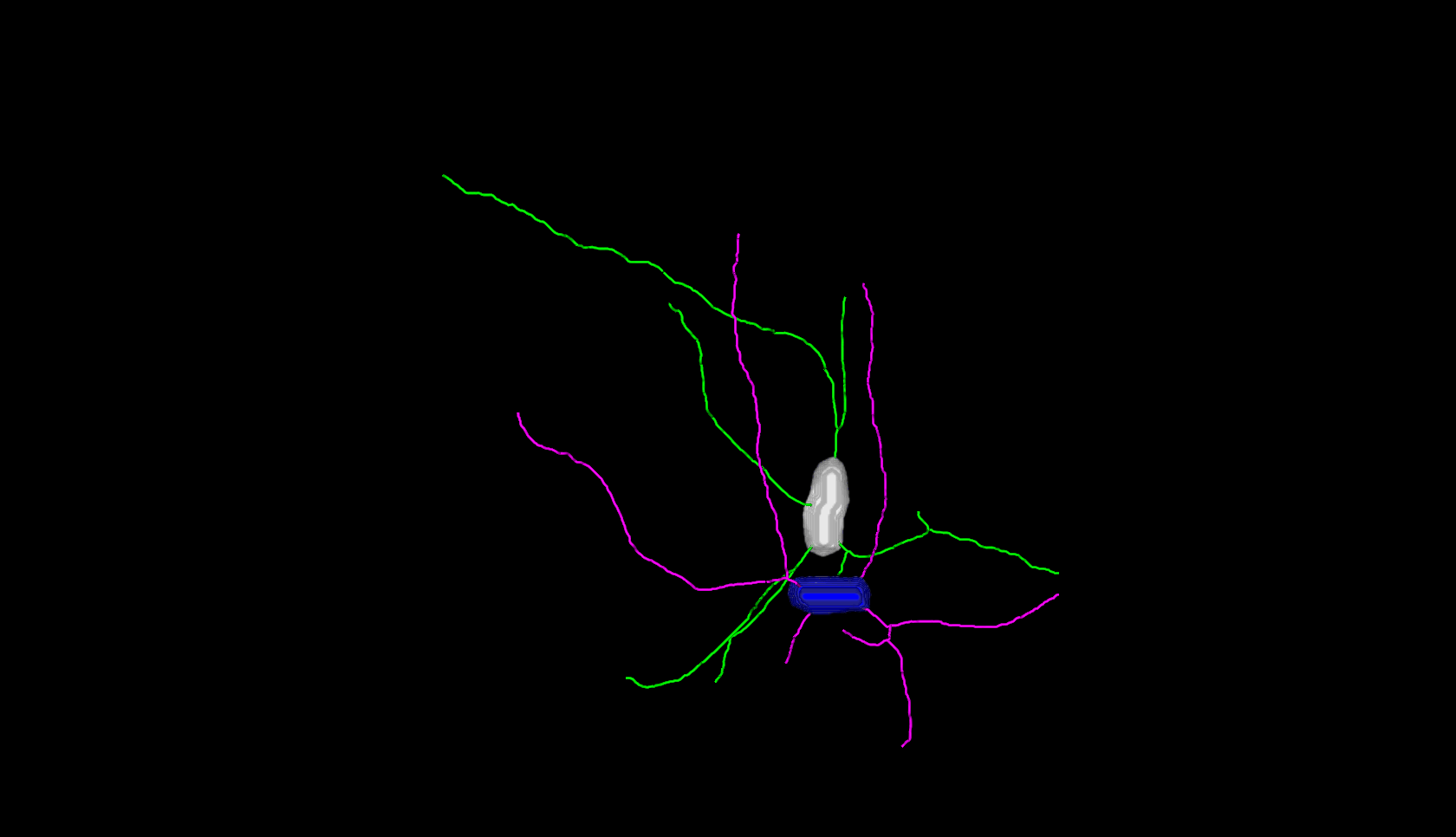

Supplement: Figure 5—source data 2. — This zip archive contains morphological images of all traced neurons grouped according their electrophysiological profiles. Each subtype’s folder contains a PDF file (with the list of neurons, their original location within the VTA, images of the traced morphology and individual Sholl curves) and two subfolders: ‘3D_gif’ – with *.gif files of the listed neurons; and ‘WaveFront_3D_obj’ – with corresponding *.obj files. *.gif files can be opened by any image viewer. *.obj files save information about the 3D model of the neurons and can be opened/reused with any 3D viewer or graphic software. [file elife-59328-fig5-data2.zip › Morphology_Source/HFF/3D_gif/6+7.gif]

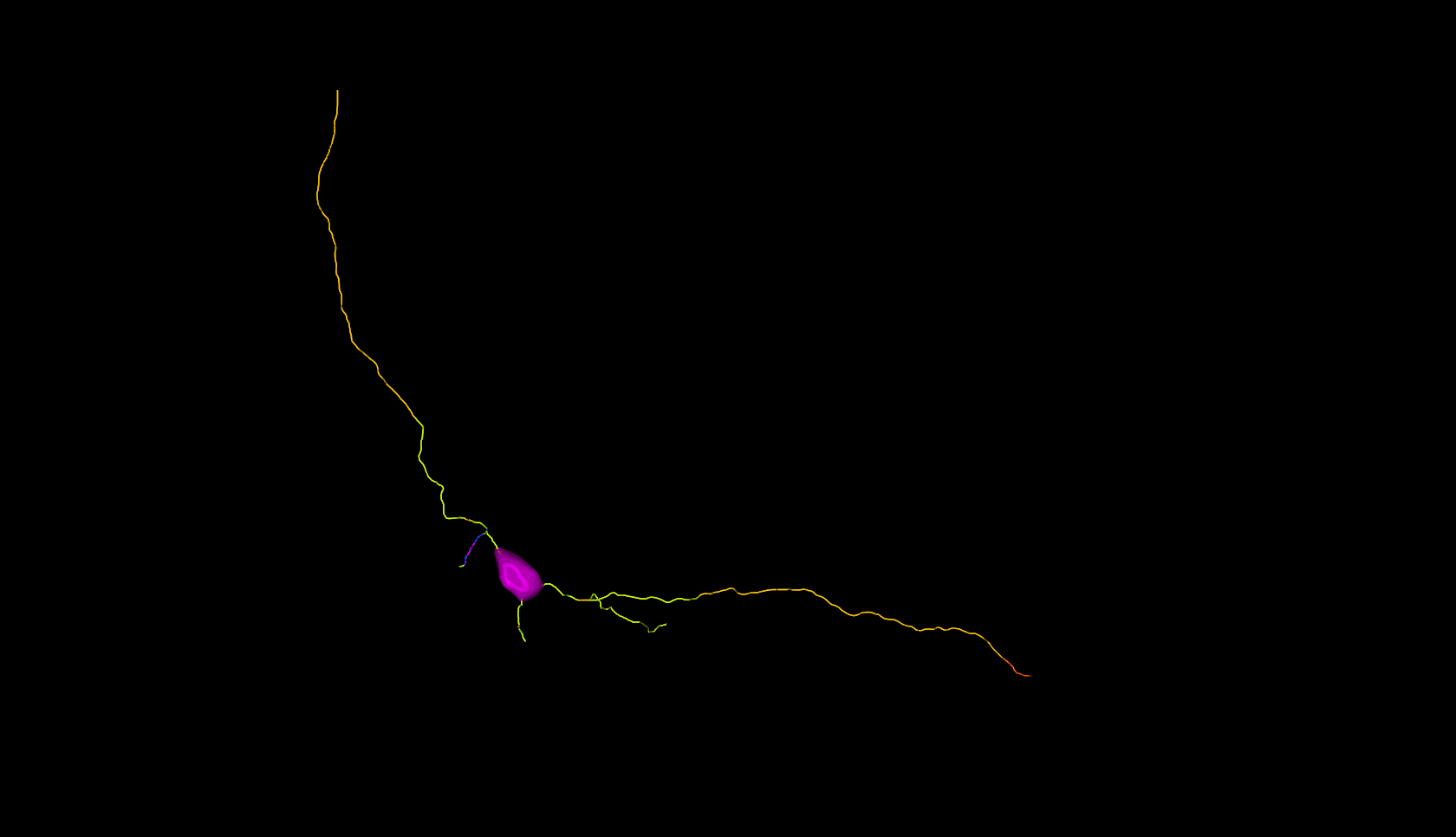

Supplement: Figure 5—source data 2. — This zip archive contains morphological images of all traced neurons grouped according their electrophysiological profiles. Each subtype’s folder contains a PDF file (with the list of neurons, their original location within the VTA, images of the traced morphology and individual Sholl curves) and two subfolders: ‘3D_gif’ – with *.gif files of the listed neurons; and ‘WaveFront_3D_obj’ – with corresponding *.obj files. *.gif files can be opened by any image viewer. *.obj files save information about the 3D model of the neurons and can be opened/reused with any 3D viewer or graphic software. [file elife-59328-fig5-data2.zip › Morphology_Source/HFF/3D_gif/8.gif]

## Location

## Traced morphology

## Sholl curve

1  
HFF

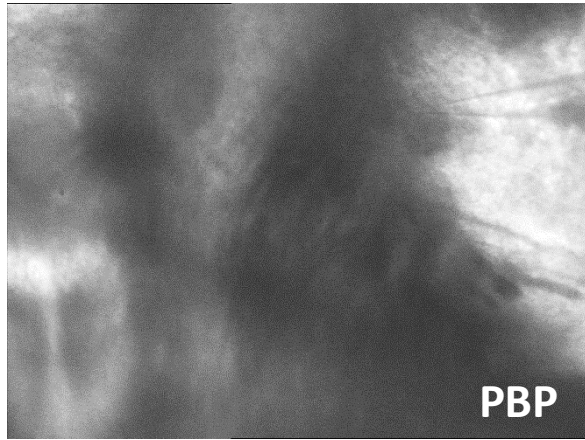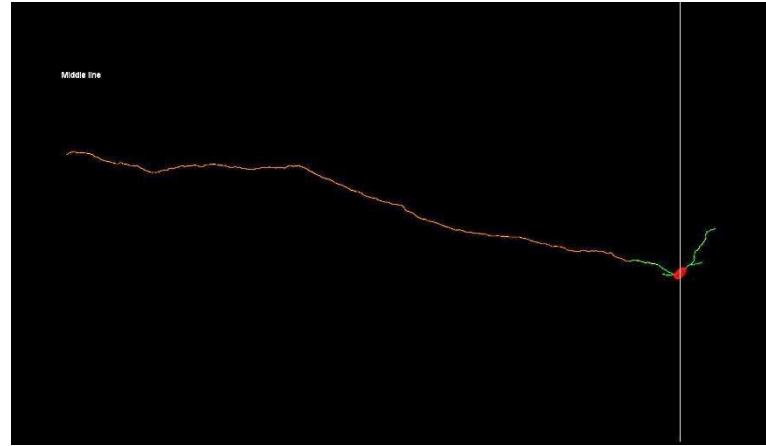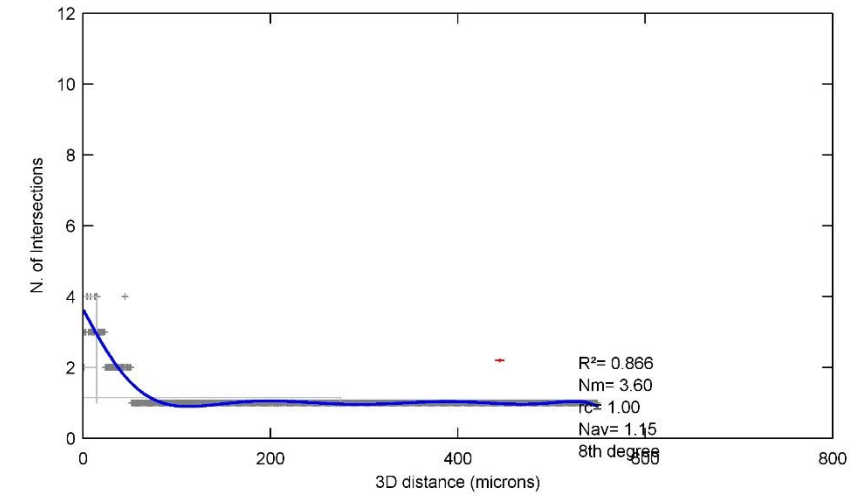

2  
HFF

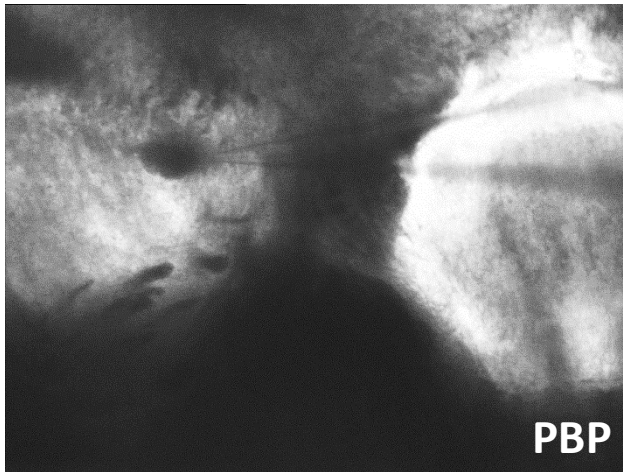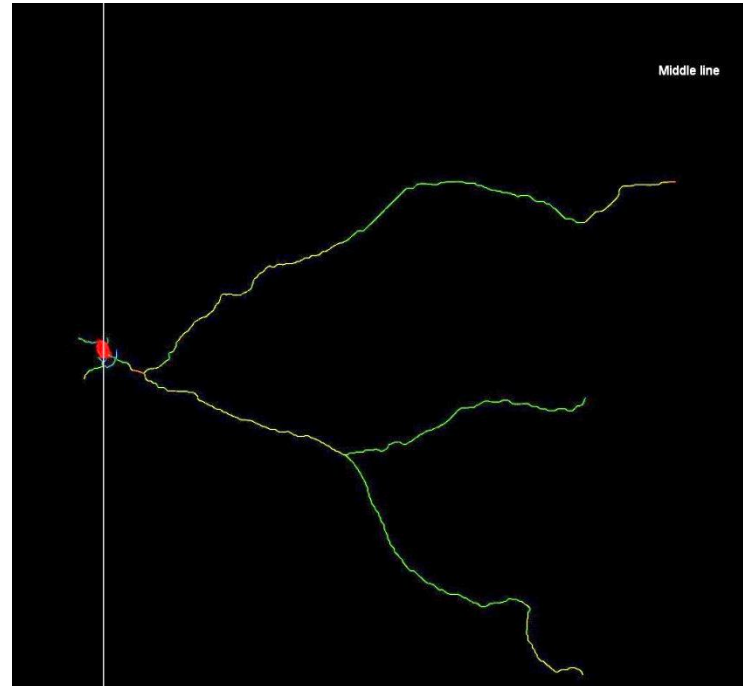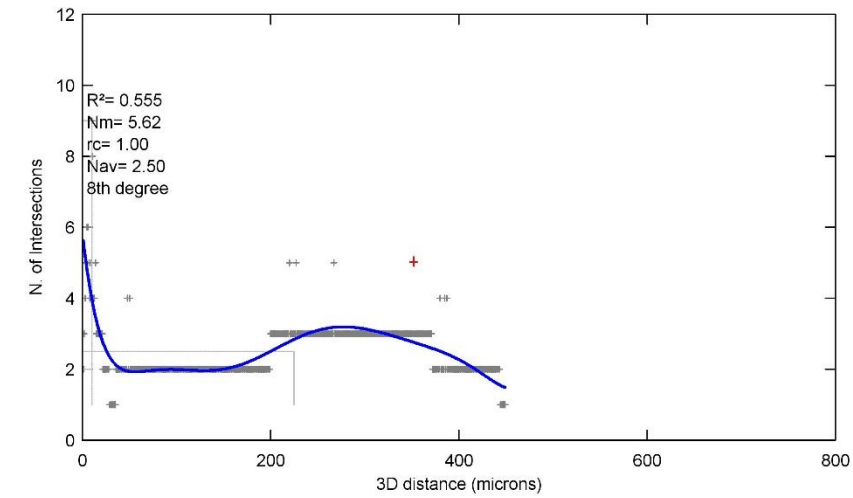

# Neurobiotin

3  
HFF

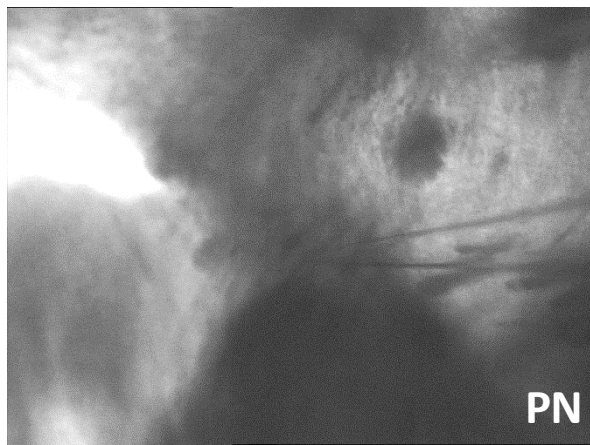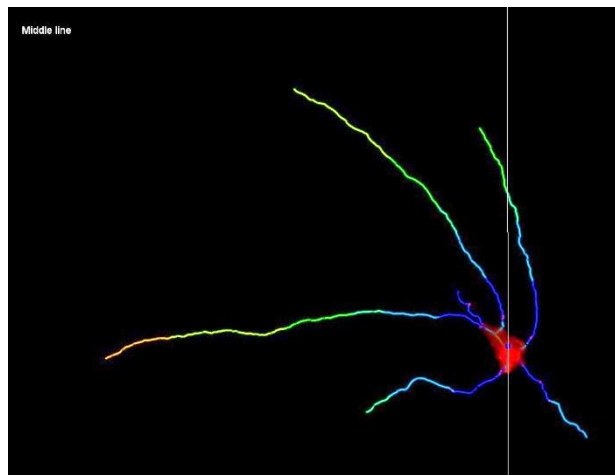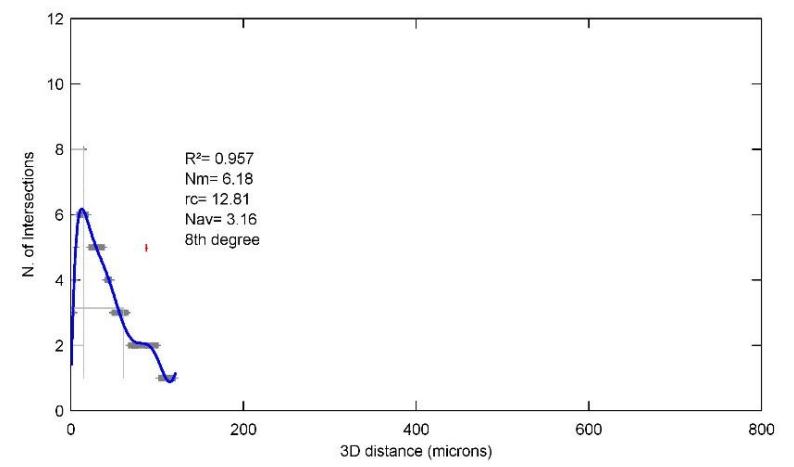

4  
HFF

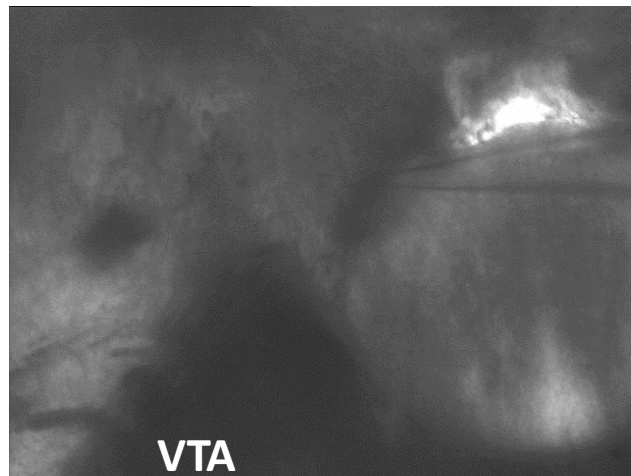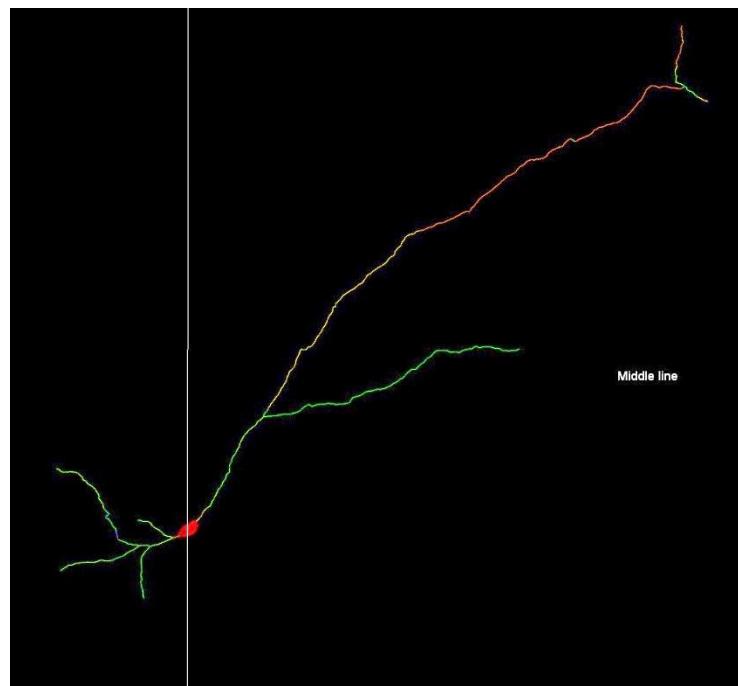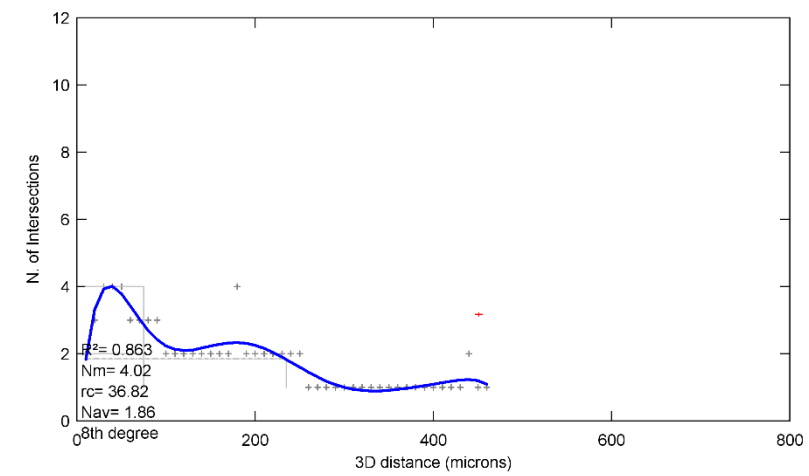

5  
HFF

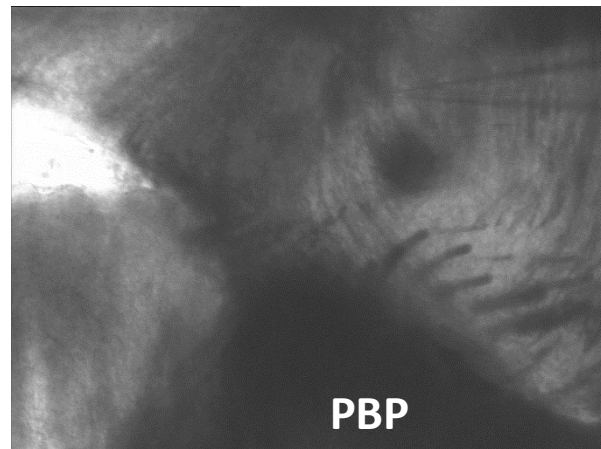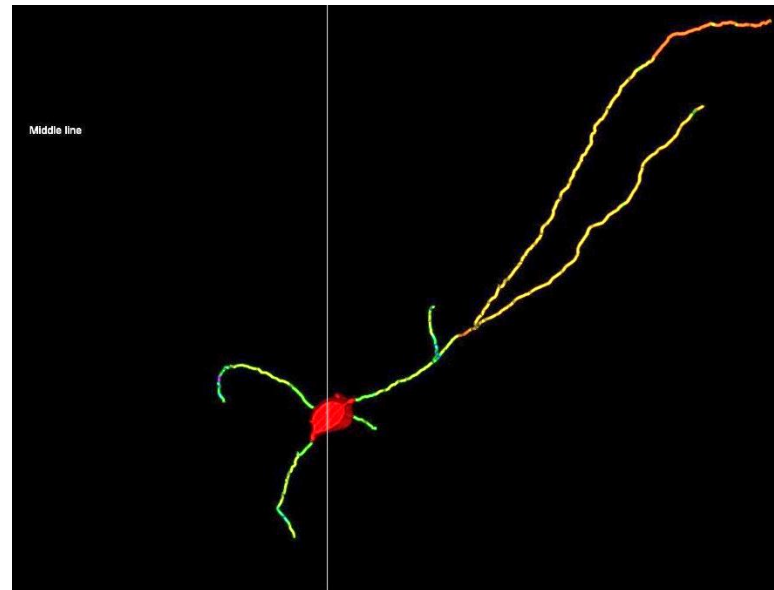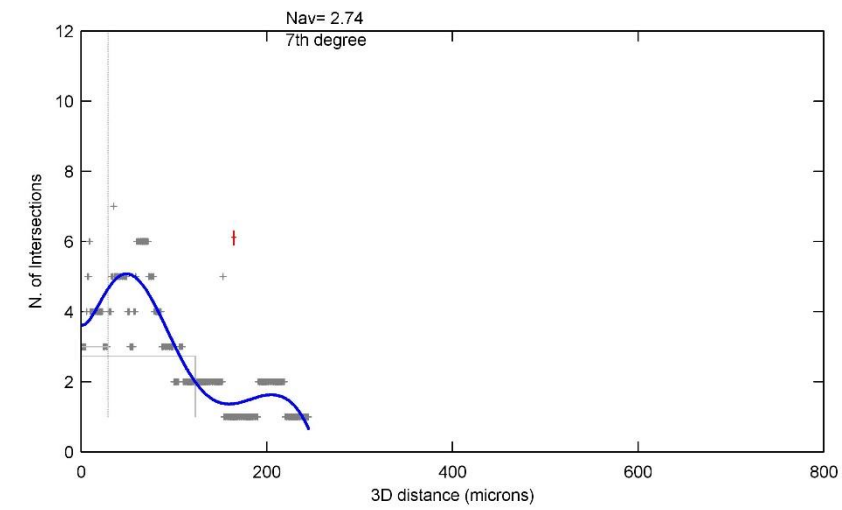

6,7  
HFF

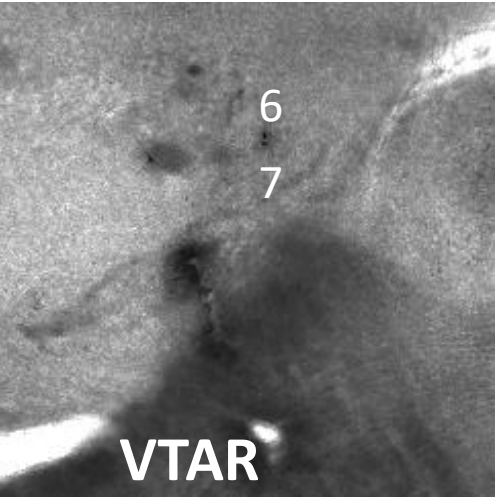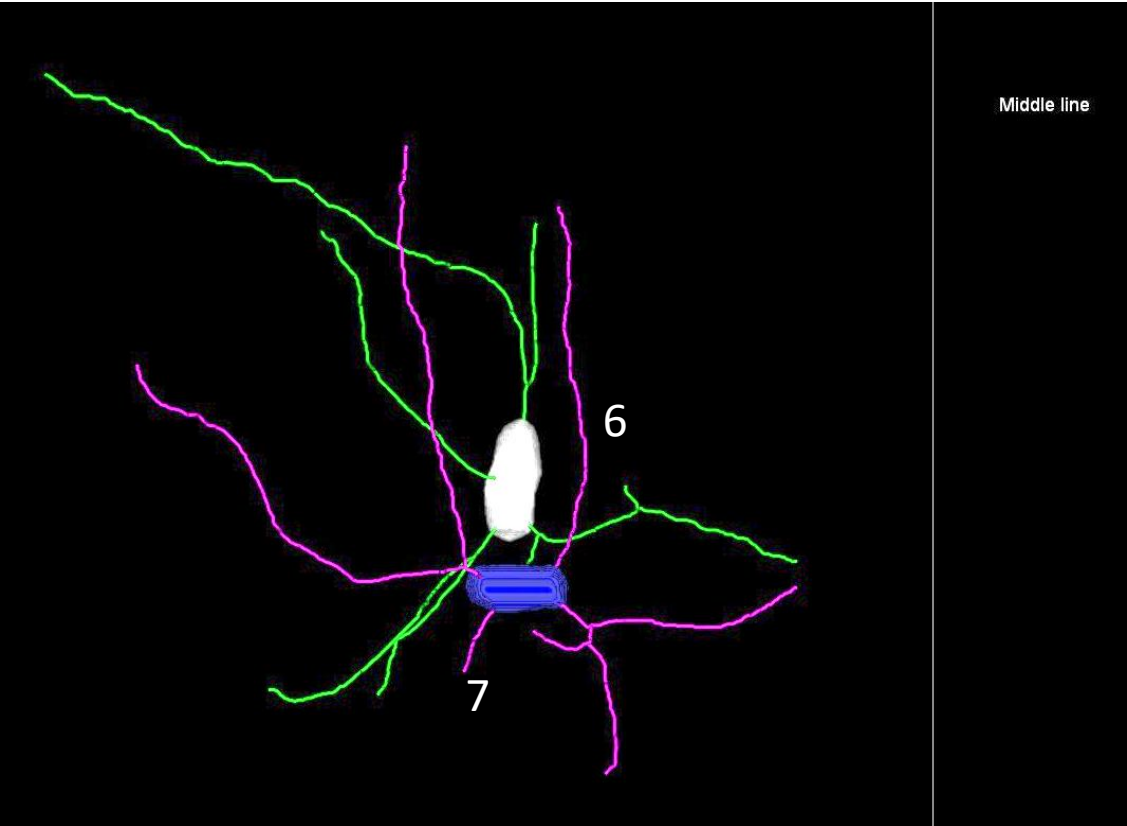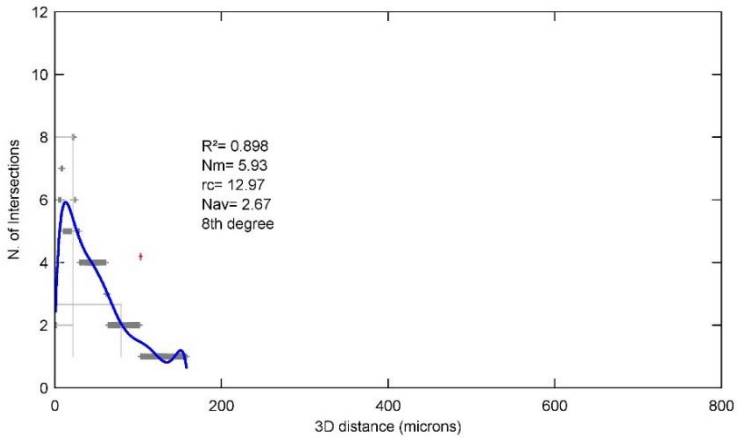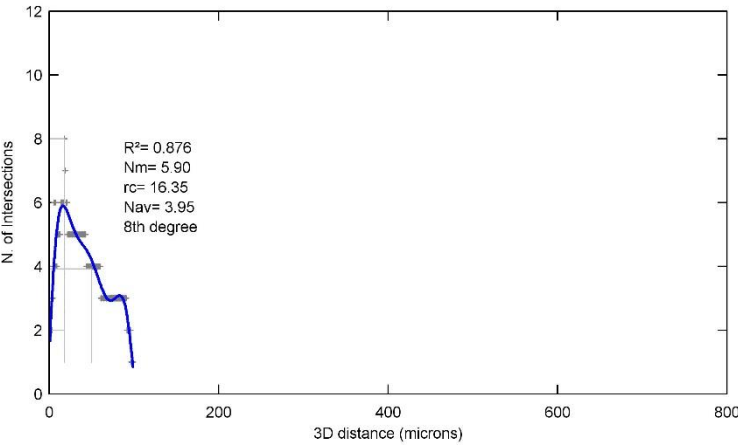

8  
HFF

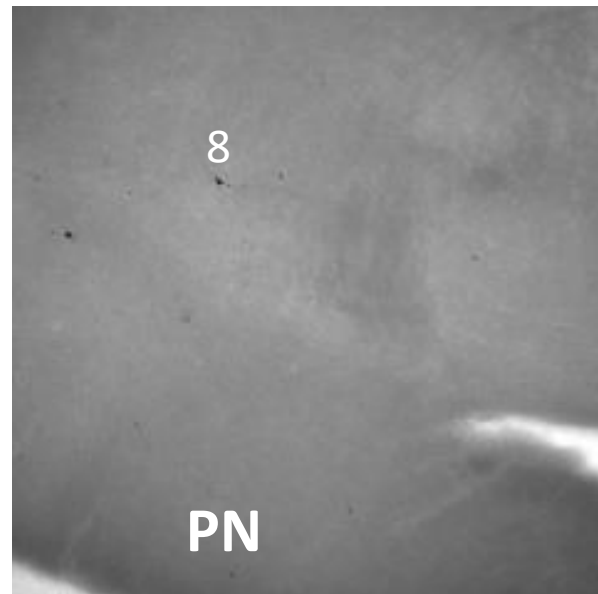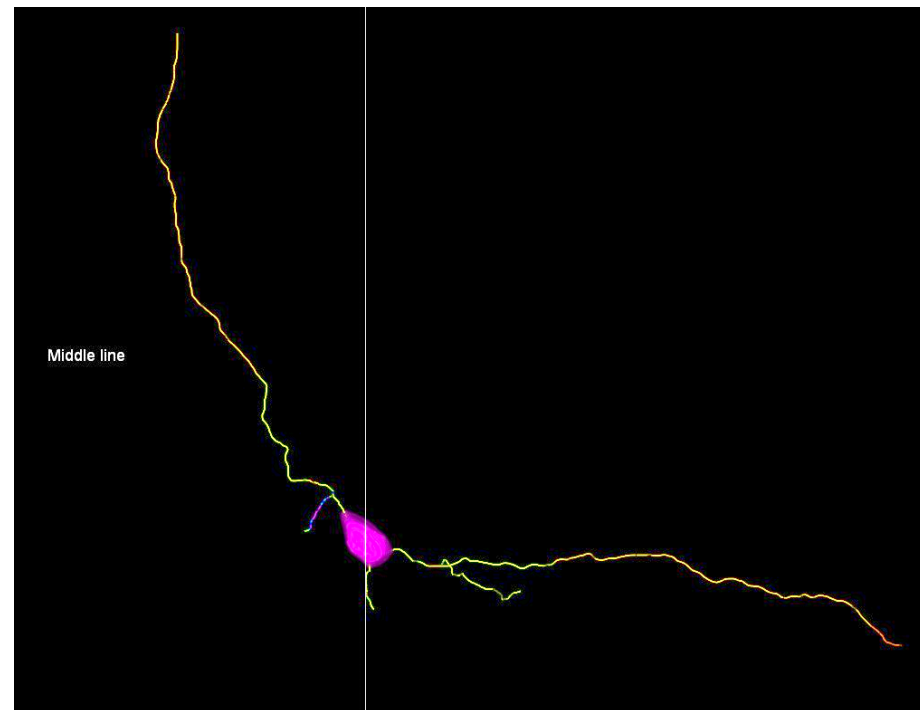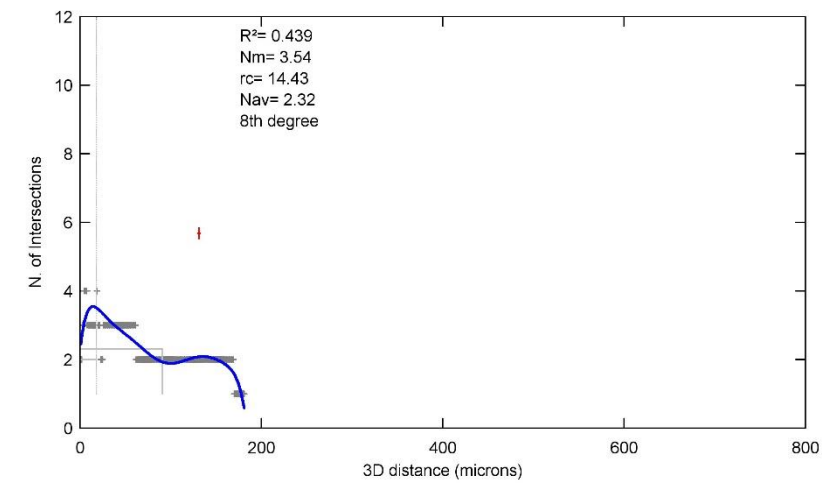

Supplement: Figure 5—source data 2. — This zip archive contains morphological images of all traced neurons grouped according their electrophysiological profiles. Each subtype’s folder contains a PDF file (with the list of neurons, their original location within the VTA, images of the traced morphology and individual Sholl curves) and two subfolders: ‘3D_gif’ – with *.gif files of the listed neurons; and ‘WaveFront_3D_obj’ – with corresponding *.obj files. *.gif files can be opened by any image viewer. *.obj files save information about the 3D model of the neurons and can be opened/reused with any 3D viewer or graphic software. [file elife-59328-fig5-data2.zip › Morphology_Source/HFF/HFF_list.pdf]
