# Supplementary material for: Heterogeneous somatostatin-expressing neuron population in mouse ventral tegmental area
Source: eLife. 2020 Aug 4;9:e59328. doi: 10.7554/eLife.59328 (PMC7440918; doi:10.7554/eLife.59328)
Supplement: Transparent reporting form [file elife-59328-transrepform.pdf]

eLIFE
Westbrook Centre, Milton Road
Cambridge CB4 1YG
UK
P 01223 855340
W elifesciences.org
T @elife

# *eLife's* transparent reporting form

We encourage authors to provide detailed information *within their submission* to facilitate the interpretation and replication of experiments. Authors can upload supporting documentation to indicate the use of appropriate reporting guidelines for health-related research (see EQUATOR Network), life science research (see the BioSharing Information Resource), or the ARRIVE guidelines for reporting work involving animal research. Where applicable, authors should refer to any relevant reporting standards documents in this form.

If you have any questions, please consult our Journal Policies and/or contact us: editorial@elifesciences.org.

**Sample-size estimation**

- You should state whether an appropriate sample size was computed when the study was being designed
- You should state the statistical method of sample size computation and any required assumptions
- If no explicit power analysis was used, you should describe how you decided what sample (replicate) size (number) to use

Please outline where this information can be found within the submission (e.g., sections or figure legends), or explain why this information doesn't apply to your submission:

Sample size (n=400) for firing pattern analysis was defined heuristically so that the cell subtype with prevalence of 10% would have at least 40 cells in the sample to be reliably identified as a distinct cell subtype. Considerations for sample sizes of other techniques and supporting experiments included minimizing the sacrifice of experimental animals and reasonable time constraints.Detailed information on sample sizes used in each experiment are reported in the text of the corresponding subsection of the Results section.

**Replicates**

- You should report how often each experiment was performed
- You should include a definition of biological versus technical replication
- The data obtained should be provided and sufficient information should be provided to indicate the number of independent biological and/or technical replicates
- If you encountered any outliers, you should describe how these were handled
- Criteria for exclusion/inclusion of data should be clearly stated
- High-throughput sequence data should be uploaded before submission, with a private link for reviewers provided (these are available from both GEO and ArrayExpress)

Please outline where this information can be found within the submission (e.g., sections or figure legends), or explain why this information doesn't apply to your submission:

All three cell subtypes, identified by a clustering algorithm in juvenile animals (P17-P23) were also found to be present in samples (n=92) taken independently from adult animals (P55-P90), confirming reproducibility of the main finding. This is stated in the text of the last paragraph of "Patch-clamp recordings revealed three distinct electrophysiological subtypes of Sst neurons in the VTA area" subsection of the Results section.

eLife Sciences Publications, Ltd is a limited liability non-profit non-stock corporation incorporated in the State of Delaware, USA, with company number 5030732, and is registered in the UK with company number FC030576 and branch number BR015634 at the address Westbrook Centre, Milton Road Cambridge CB4 1YG, UK | March 2019

**Statistical reporting**

- Statistical analysis methods should be described and justified
- Raw data should be presented in figures whenever informative to do so (typically when N per group is less than 10)
- For each experiment, you should identify the statistical tests used, exact values of N, definitions of center, methods of multiple test correction, and dispersion and precision measures (e.g., mean, median, SD, SEM, confidence intervals; and, for the major substantive results, a measure of effect size (e.g., Pearson's r, Cohen's d)
- Report exact p-values wherever possible alongside the summary statistics and 95% confidence intervals. These should be reported for all key questions and not only when the p-value is less than 0.05.

Please outline where this information can be found within the submission (e.g., sections or figure legends), or explain why this information doesn't apply to your submission:

```
Descriptive statistics summarizing the quantitative properties of the studied cells are pre-
sented in the main text of the results section and always include sample size, mean and stan-
dard error of the mean.

Clustering algorithm, model selection and cross-validation procedures are described in detail
in subsection "Clustering using electrophysiological features" of the Methods section.
```

(For large datasets, or papers with a very large number of statistical tests, you may upload a single table file with tests, Ns, etc., with reference to sections in the manuscript.)

**Group allocation**

- Indicate how samples were allocated into experimental groups (in the case of clinical studies, please specify allocation to treatment method); if randomization was used, please also state if restricted randomization was applied
- Indicate if masking was used during group allocation, data collection and/or data analysis

Please outline where this information can be found within the submission (e.g., sections or figure legends), or explain why this information doesn't apply to your submission:

Due to the fact that the main finding comprised identifying distinct cell subtypes with data-driven methods a priori group allocation was not applied. Individual cells were assigned to their respective groups using a clustering algorithm followed by cross-validation procedure described in the Methods section (see Clustering using electrophysiological features).

**Additional data files ("source data")**

- We encourage you to upload relevant additional data files, such as numerical data that are represented as a graph in a figure, or as a summary table
- Where provided, these should be in the most useful format, and they can be uploaded as "Source data" files linked to a main figure or table
- Include model definition files including the full list of parameters used
- Include code used for data analysis (e.g., R, MatLab)
- Avoid stating that data files are "available upon request"

Please indicate the figures or tables for which source data files have been provided:

Features extracted from the electrophysiological data (specified in Methods Table within Material and Methods section)) and the code used to reproduce the results shown in Figures 3 and Supplementary Figures S2-S3 can be downloaded from: https://version.aalto.fi/gitlab/zubarei1/clustering-for-nagaeva-et.-al.-sst-vta.
PatchSeq scRNA-seq raw data and expression matrix used for Figure 6, Supplementary Fig. S6 and S10 have been deposited in the ArrayExpress database at EMBL-EBI (www.ebi.ac.uk/arrayexpress) under accession number E-MTAB-8780.
